# StoatyDive: Evaluation and classification of peak profiles for sequencing data

**DOI:** 10.1093/gigascience/giab045

**Published:** 2021-06-18

**Authors:** Florian Heyl, Rolf Backofen

**Affiliations:** Bioinformatics Group, Department of Computer Science, University of Freiburg, Georges-Köhler-Allee 106, 79110 Freiburg, Germany; Bioinformatics Group, Department of Computer Science, University of Freiburg, Georges-Köhler-Allee 106, 79110 Freiburg, Germany; Signalling Research Centres BIOSS and CIBSS, University of Freiburg, Schaenzlestr. 18, 79104 Freiburg, Germany

**Keywords:** CLIP-Seq, data analysis, peak shape clustering, RNA, protein

## Abstract

**Background:**

The prediction of binding sites (peak-calling) is a common task in the data analysis of methods such as cross-linking immunoprecipitation in combination with high-throughput sequencing (CLIP-Seq). The predicted binding sites are often further analyzed to predict sequence motifs or structure patterns. When looking at a typical result of such high-throughput experiments, the obtained peak profiles differ largely on a genomic level. Thus, a tool is missing that evaluates and classifies the predicted peaks on the basis of their shapes. We hereby present StoatyDive, a tool that can be used to filter for specific peak profile shapes of sequencing data such as CLIP.

**Findings:**

With StoatyDive we are able to classify peak profile shapes from CLIP-seq data of the histone stem-loop-binding protein (SLBP). We compare the results to existing tools and show that StoatyDive finds more distinct peak shape clusters for CLIP data. Furthermore, we present StoatyDive’s capabilities as a quality control tool and as a filter to pick different shapes based on biological or technical questions for other CLIP data from different RNA binding proteins with different biological functions and numbers of RNA recognition motifs. We finally show that proteins involved in splicing, such as RBM22 and U2AF1, have potentially sharper-shaped peaks than other RNA binding proteins.

**Conclusion:**

StoatyDive finally fills the demand for a peak shape clustering tool for CLIP-Seq data that fine-tunes downstream analysis steps such as structure or sequence motif predictions and that acts as a quality control.

## Findings

### Background

The biological function of a protein is determined by its interaction partners and the mode of interaction. Studying these interactions broadens our horizon about the cellular mechanisms such as alternative splicing and post-transcriptional regulation. Cross-linking immunoprecipitation in combination with high-throughput sequencing (CLIP-Seq) fathoms these interactions. CLIP-Seq investigates all interactions between an RNA binding protein (RBP) and its target RNAs [1]. CLIP-Seq thus scrutinizes the post-transcriptional regulation by RBPs. Prediction of binding regions (peak-calling) is a crucial step in the data analysis of methods such as CLIP-Seq. Before the peak analysis there is typically no evaluation and classification of the peak characteristics. Yet, the obtained peak set might have different peak profiles that are worth filtering to refine a downstream analysis. The different peak shapes are the result of several biological and technical problems.

Many RBPs have several binding domains with different binding affinities, and are often part of protein complexes, leading to an intricate binding pattern. As described in a review by Jankowsky and Harris [2], there are specific and unspecific binders. Examples of unspecific binders are often RBPs that need to bind many RNAs such as messenger RNA (mRNA) export factors [3]. Another example of common unspecific binders are RNA helicases. However, even more specific RBPs bind RNAs in a large range of affinities, indicating that different binding sites vary in their binding specificity. While many factors, such as the affinity of an RBP for the binding site and the concentration of the protein and RNA, influence the binding specificity, it is likely that these factors are manifested in the CLIP binding profile landscape. At this point, however, no tool exists that can be used to study this possibility in more detail.

In addition, technical biases might change the peak profile landscape. Binding artifacts might be introduced during read library preparation. Protocol biases, e.g., PAR-CLIP biases that are introduced by endonuclease and photoactivatable nucleosides [4], might also affect the binding site predictions. In addition, the peak caller itself might generate specific peak profiles and false-positive results, which the user might not want to have in their data.

**Figure 1:**
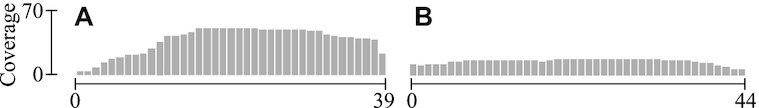
We show 2 significant peaks of a CLIP experiment for the protein SLBP (ENCSR483NOP, replicate 2). One can see peaks with drastically different peak profiles, pointing towards more specific (A) or unspecific (B) binding. Current analysis of CLIP binding sites is typically based on manual inspection of a few peaks. Thus a general tool is missing that allows peak profiles to be filtered, clustered, and quantified, therefore refining downstream analysis tasks for data such as CLIP. StoatyDive assists in finding and distinguishing peaks like (A) and (B).

This leads to many questions in the data analysis of binding sites that currently cannot be answered adequately. Examples are: Does my protein of interest bind generally specifically (Fig. 1A) or unspecifically (Fig. 1B)? Does my RBP of interest have >1 binding motif? Does my experiment have any quality issues, meaning, do my reads come from unspecific bindings because of library preparation artifacts? Does my protocol generate biases? Do I have false-positive reults in the set of predicted peaks from my peak caller of choice?

We hereby present StoatyDive, a tool to evaluate and classify peak profiles to help answer the aforementioned questions. StoatyDive uses the whole peak profile, as well as predefined features, to do a peak shape clustering for sequencing data. In this article, we test StoatyDive on CLIP data of the eCLIP protocol from the histone stem-loop-binding protein (SLBP) from the study by Van Nostrand et al. [5]. SLBP has been reported to be a histone mRNA export and translation factor [6]. StoatyDive delivers several plots and a table to assess the different binding profiles of a protein. The tool assists in the selection of specific and unspecific binding sites and in finding similar shaped peak profiles. Thus, we try to refine the obtained peaks of the SLBP data to find more specific sites of SLBP. It also helps as a quality assessment to validate a CLIP-Seq or any other binding experiment. Later in the article, we use StoatyDive to investigate the peak profile landscape of different RBPs with different biological functions and different numbers of RNA recognition motifs (RRM). StoatyDive comes with some test data and a quick installation guide.

### Data preparation of SLBP and analysis

We used eCLIP data of the protein SLBP (ENCSR483NOP; GSE91802 [5]). The data comprised 2 CLIP replicates and a size-matched input control from immortalized myelogenous leukemia cells (K562). We processed the data with the snakemake pipeline SalamiSnake [7] (v0.0.1) for eCLIP data. SLBP has been reported to be cytoplasmic but to be present also in the nucleus [6]. Thus, we mapped the reads against the human genome (version hg38) with STAR [8], also taking the transcriptome into account. We predicted potential binding sites of SLBP with PureCLIP [9], which we ran for each CLIP replicate separately, taking the input control into account. We extended the predicted binding regions by 20 nucleotides left and right because PureCLIP often underestimates the binding region. We further fused the predicted peaks from each CLIP replicate with BEDTools [10] to get a robust set of predicted binding sites, which resulted in 899 robust peaks. We executed StoatyDive (v1.1.0 with umap v0.2.5.0) with length normalization, a penalty for broader plateaus, and peak profile smoothing. The complete call was as follows: StoatyDive.py -a peaks.bed -b reads.bam -c hg38.chrom.sizes.txt --peak_correction --scale_max 10 --border_penalty --sm.

### Peak profile landscape reveals variability of binding sites

**Figure 2:**
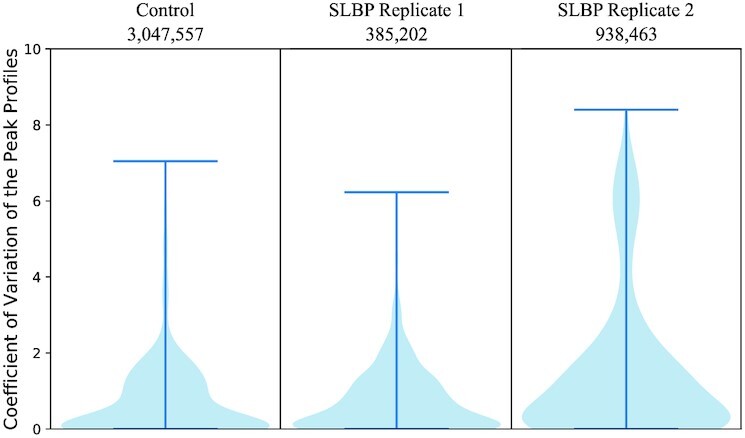
StoatyDive generates a coefficient of variation (CV) distribution to evaluate the peak profile shapes, which can be used as a quality control. The CV distribution of the peak profiles of the input control and Replicate 1 of the SLBP CLIP-Seq experiment are quite similar. In contrast, the CV distribution of Replicate 2 is different. The number of uniquely mapped reads is listed below the sample name.

The user obtains from StoatyDive a distribution of the coefficient of variation (CV), calculated for each peak, to get a broad overview of the peak profile landscape of their experiment (see Methods). Broader peaks tend to have a CV ≈0. Although the CV distributions of the input control and Replicate 1 of the SLBP data differed significantly (1-sided Wilcoxon test *P*-value = 0.03), both contained a lot of regions with a CV ≈0 (Fig. 2, both with a mean CV of 0.47). In contrast, the CV distribution of Replicate 2 was distinct (P-value < 0.05 to input control and Replicate 1) because it had more peaks with a higher CV (mean CV of 1.41) and thus more specific binding events (e.g., Fig. 1A, CV ≈ 5.3). Yet, some potential binding sites were more unspecific with a CV ≈0 (e.g., Fig. 1B, CV ≈ 0.0003).

The CV distribution of the input control was expected because an ideal control experiment should contain no real or not enriched binding events; i.e., the value of all CVs is expected to be very small and close to 0 (see Supplementary Fig. S1). However, the CV distribution of Replicate 1 was more similar to the control experiment than to Replicate 2. A CLIP experiment should result in a peak set with enriched regions and thus more specific peaks. The distribution of Replicate 1 indicates some degree of variability in the binding events. However, we have to stress that this does not necessarily depict a poor quality of Replicates 1 and 2. For a downstream analysis, e.g., the prediction of sequence motifs, it is worth investigating why the CV distribution of Replicate 1 was very different from that of Replicate 2. A sequence motif prediction depends on the selected binding sites; thus, StoatyDive’s inspection allows the user to assess the binding sites using the CV distributions. This helps to appraise whether SLBP has different binding mechanisms, which we further investigated and discussed in section ”Information from peak profile shapes.”

We checked the robustness of the CV distribution and split the second replicate into 2 pseudo-replicates. We randomly selected 50%, with and without replacement, of the reads for Pseudo-replicate 1 and the other half for Pseudo-replicate 2. Both scenarios gave us similar results. Without replacement, Replicate 1 had a mean CV of ≈1.05 and Replicate 2 a value of ≈1.03, with a *P*-value of 2-sided Wilcoxon test of 0.768. With replacement, Replicate 1 had a mean CV of ≈1.05 and Replicate 2 a value of ≈1.02, with a *P*-value of the Wilcoxon test of 0.7546. For example, the sharp peak in Fig. 1A had a CV in both scenarios of ≈5.2 and ≈5.3 for Pseudo-replicate 1, and ≈4.8 and ≈5.4 for Pseudo-replicate 2. The peak Fig. 1A was in the peak list on position 47 and 42 for Pseudo-replicate 1, and 52 and 45 for Pseudo-replicate 2. The broad peak Fig. 1B had a CV ≈0.001 (position 662) and ≈0.001 (position 658) for Replicate 1, and ≈0.001 (position 667) and ≈0.001 (position 655) for Replicate 2. Each peak retained its sharp or broad shape despite the random sampling. Thus, the overall trend of the CV distribution and the peak position in the list were robust to including random noise.

Checking the CV distributions of other CLIP-Seq datasets such as TAF15, TARDBP, and HNRNPA1 (Supplementary Fig. S1), which we analyze further in a later section, we saw that the CV distributions also had differences between the replicates (2-sided Wilcoxon test *P*-value <0.05). However, this does not mean a low quality of the data and just highlights that it is important to do replicates in order to quantify biological and technical variance as noted in a previous CLIP study [11].

**Figure 3:**
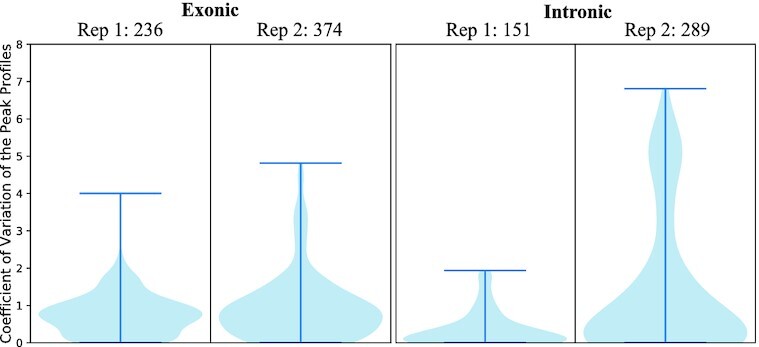
Coefficient of variation (CV) distributions of exonic and intronic peaks reveal a difference in the profile shapes. The CV distributions of the peak profiles of Replicates 1 and 2 are more similar in exonic regions than in intronic ones. The number of peaks is listed next to the sample names. The ratio of exonic and intronic peaks is ∼1.6 for Replicate 1 and ∼1.3 for Replicate 2.

To investigate further differences between the 2 replicates, we split the peak set into peaks overlapping with exons and introns (see Fig. 3). SLBP is a translation and transport factor, which is present in the cytoplasm as well as the nucleus [6,12]. Therefore, the replicates could have different binding events, where one replicate might have more events in cytoplasm and the other more in the nucleus. Replicate 2 had 136 exonic and 136 intronic peaks more than replicate 1. Notably, we can see a CV difference when comparing the intronic peaks of the 2 replicates, with a mean CV of 0.23 for Replicate 1 and 1.26 for Replicate 2 (1-sided Wilcoxon test *P*-value <0.05). The exonic peaks on the other hand were more similar (mean CV = 0.48 and 0.90, respectively), but the CV distributions were still significantly different (1-sided Wilcoxon test *P*-value <0.05). Therefore, StoatyDive showed a variability of binding events (intronic vs exonic) between the 2 replicates.

### The 7 different peak shapes in the SLBP data

**Figure 4:**
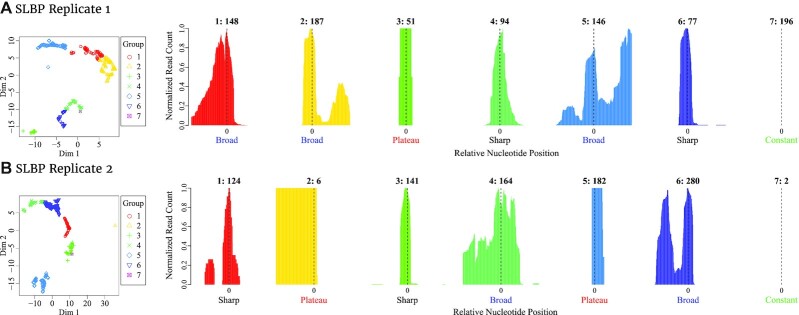
Results of the peak profile clustering with StoatyDive (procedure described in the Methods). We applied StoatyDive to the SLBP data [5]. StoatyDive found 7 different peak profile shapes in the data of Replicate 1 (A1–7) and Replicate 2 (B1–7) of SLBP. We present 1 example profile for each cluster with the number of peaks on top. For Replicates 1 and 2 we could separate between very thin and specific mountains such as Fig. 1A and very broad profiles like Fig. 1B. We also found peaks shaped like plateaus, such as 3A, and constant peaks, e.g., 7A. The profiles also vary slightly between groups. For example, 6B has >1 spiky mountain in contrast to 1B.

For a more detailed analysis, we classified the peaks of Replicates 1 and 2 with the help of StoatyDive (Fig. 4). The procedure is described in the Methods. StoatyDive found 7 distinguishable peak profiles for both Replicates 1 and 2. We looked more closely at the profiles of Replicate 2 (Fig. 4B). Clusters 2 and 5, which are set apart clearly from clusters 1, 3, 4, and 6, are characterized by plateau-shaped profiles. The other groups had profiles with mountain-like shapes with peaks tending to become broader and fuzzier on the order of clusters 3, 1, 6, and 4. To return to our initial examples (Fig. 1), peak profile Fig. 1A was classified by StoatyDive as a small, centered mountain (Fig. 4B3), whereas peak profile Fig. 1B was classified as a very broad profile (Fig. 4B4).

It is noteworthy that constant profiles (Fig. 4) represent a constant read coverage throughout the whole peak. Because of the max-min normalization of the profile (see Methods), the value becomes 0, i.e., the profile is not empty. In contrast, peaks shaped like plateaus do not have a constant value because their coverage changes at a few positions. Furthermore, the number of clusters depends on the optimization of StoatyDive but can also be defined by the user.

The great distance between clusters 2 and 5 is the result of the difference between the profile borders (cf. Fig. 4B2 and B5). Where cluster 2 had profiles with lots of values in the left or right side of the peak profile, cluster 5 occupies the center of the peak profile.

SLBP has been reported as an mRNA export and translation factor [6]. Thus, it is worth investigating whether peaks like Fig. 1A are more informative for a translation factor than peaks like Fig. 1b. That is to say, Fig. 1A might be more suited for sequence and structure predictions than peak Fig. 1B. Therefore, we perform a deeper inspection of groups 1, 3, 4, and 6 in the next section of the article.

We also checked the uniqueness of the shapes by analyzing the peaks based on the reads from the size-matched input control (see Supplementary Fig. S2). StoatyDive had just identified 4 different clusters, encompassing mountain-shaped peaks, as well as plateaus, and constant peaks. It was to be expected that similar shapes would be found in the control because the biggest challenge for peak-calling is the identification of enriched sites with different shapes between control and CLIP data [13,14]. In a future version of StoatyDive, we will include a mode to check peak shapes between samples to see whether we could improve peak-calling results with a peak shape comparison.

### Information from peak profile shapes

We made the assumption that Replicate 1 might have more unspecific and fewer distinguishable profiles than Replicate 2 based on the different CV distributions (Fig. 2). Thus, we counted the number of peaks in each cluster for Replicates 1 and 2 (Table 1). From our robust 899 peaks, in Replicate 1 we had $\approx 19\%$ peaks with a sharp mountain shape (Fig. 4A4 and A6), $\approx 53\%$ with a broader mountain shape (Fig. 4A1, A2, and A5), $\approx 6\%$ peaks with plateaus (Fig. 4A3), and $\approx 22\%$ constant shaped peaks (Fig. 4A7). Replicate 2, on the other hand, had $\approx 29\%$ sharply mountain-shaped peak profiles (see Fig. 4B1 and B3), so 94 more than Replicate 1. This corroborated the assumption that Replicate 1 had broader and more unspecific sites. Thus, Replicate 2 had only $\approx 49\%$ broad peak profiles (Fig. 4B4 and B6) and only 2 constant peak profiles (Fig. 4B7). Yet, Replicate 2 had $\approx 21\%$ peaks with plateaus (Fig. 4B2 and B5).

**Table 1:** Number of peaks of SLBP for different shape groups

Replicate	Total	Sharp	Broad	Plateau	Constant
	**Number of peaks**				
1	899	171	481	51	196
2	899	265	444	188	2
	Peak summits in histone mRNAs				
1	116	22	86	6	2
2	118	42	71	5	0

We further investigated the biological function of different peak profiles of Replicate 2. Because SLBP targets histone mRNAs [6], we intersected known annotated mRNAs of histones with the peaks of the different profile clusters (Table 1). From the 899 peaks, only $\approx 13\%$ of Replicate 2 overlapped with mRNAs of histones. Yet, of these 118 peaks almost all came from groups 1, 3, 4, and 6. These groups were either spiky, or broader mountain-shaped peak profiles. For example, we found a sharper peak located on RNU7-1 RNA (U7 small nuclear 1) that contains a stem loop that might be potentially targeted by SLBP [15]. The peak got a CV of 3.9 and was classified into the peak profile cluster 3 of Replicate 2. Only 5 peaks intersected with histone mRNAs that had a profile shaped like a plateau (Fig. 4B5). This endorsed the assumption that peak profiles shaped like plateaus were less informative. The observation also suggests that broader profiles were still informative because some of them overlapped with histone mRNA.

Next, we used MEME-ChIP (MEME Suite - Motif-based sequence analysis tools, RRID:SCR_001783) [16] to search for sequence motifs associated with the different peak shape groups of the second replicate of SLBP. We found 2 significantly enriched motifs associated with the plateau peaks and 3 motifs associated with the sharper peaks (Table 2). Yet, both the plateaus and the sharper peaks had 2 similar sequence motifs. Both motifs (G)GCUCUUU(U) and (CA)GAGCCA(C) were more highly enriched in the sharper-shaped peaks. On the other hand, we found >10 enriched motifs for the broader-shaped peaks. The motifs of that peak set were very different from the motifs of the plateaus and sharper-shaped peaks. Even the first 3 significantly enriched motifs had more noise and consequently were less enriched than the motifs of the other 2 peak shape groups. The E-values of all motifs were also >1,000 times higher for broader peaks than for sharper peaks. Peaks shaped like plateaus were slightly more significant than broader peaks.

**Table 2:** First 3 MEME-ChIP motifs for the different peak shape groups of SLBP with the E-value, the portion of sequences that have the motif, and the number of peaks that have the motif and also intersect with histone mRNAs

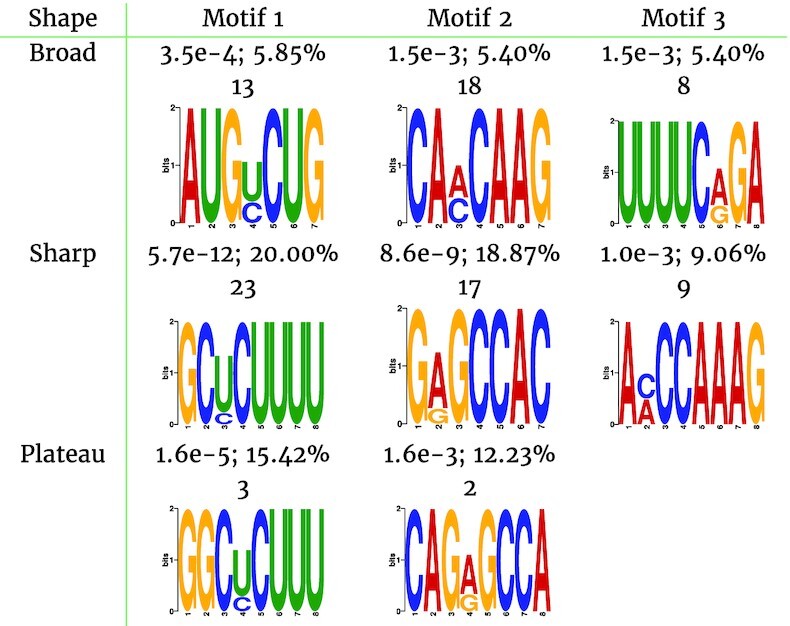

On further inspection of those peak motifs in histone mRNAs, we found that the 3 motifs of the sharper shape peaks covered 10 more histone mRNAs (49 in total) than the broader-shaped peaks (39 in total). This endorsed the observation that broader peaks encompassed more noise. For example, the second motif of the broad peaks CA(A/C)CAAG came close to the third sequence motif, with the sequence A(C/A)CCAAAG, of the sharper shape peak group. The second motif had the highest occurrence in histone mRNAs for the broader peaks. Thus, the broader peak set includes true binding sites but with some higher additional noise. Furthermore, we already showed that the plateau group might also hold some peaks that are true binding sites (Table 1), which was confirmed by the similar sequence motifs to the sharper peak set. We could confirm that the 2 sequence motifs that are present in plateaus were also present in histone mRNAs (Table 1). All in all, just the sequence motif analysis showed how different the outcome of subsequent tasks can be for different peak shape groups. Yet, we cannot confirm the biological truth behind those motifs because it requires further experiments to verify them.

### Optimizing StoatyDive with the data of SLBP

We investigated the peaks of the second replicate of SLBP further and took the CLIPper peaks (ENCFF127WAK) from the study by Van Nostrand et al. [5]. We wanted to check the specificity and sensitivity of StoatyDive for different CV cut-offs and peak sizes (see Supplementary Table S1). PureCLIP does not give an FC or *P*-value, so it was only possible to calculate those features with the CLIPper peaks. Because SLBP binds mainly histone mRNAs [6], we defined peaks in histones with a log_2_ fold change (LFC) ≥1 and a *P*-value <0.05 as true-positive results. A true-negative result was a non-significant peak in a region that did not overlap with a histone. We investigated only peaks where we could calculate a CV and used StoatyDive with a peak size of 30 (median), 40 (Q3), 70 (Q3 + 1.5 × IQR), and a maximum peak length of 201 nucleotides. The peak sizes were chosen on the basis of the length of all peaks (see Supplementary Fig. S4). The Matthews correlation coefficient (MCC) is more informative in case of imbalanced datasets, which was the case of the SLBP data. The true-negative rate (TNR) was always at 1.0 because the peak set had no peaks in histones that were not significant (no false-positive results). We investigated the cluster with the highest (main cluster) and second highest (second cluster) number of peaks in histones. Looking solely at the clustering, we achieved the highest true-positive rate (TPR) (0.69) and MCC (0.66) with a peak length of 70, higher than does the set of all CLIPper peaks with a TPR of 0.48 and MCC of 0.57. This was achieved by the second cluster, which pointed out that the cluster had to be carefully chosen in order to remove some noise in the data (artifacts). The cluster had also the highest enrichment, with a mean LFC of 3.4 (median *P*-value <0.05), higher than do all CLIPper peaks, with a value of 1.9 (median *P*-value of 0.038). Furthermore, the same peak length of 70 nucleotides achieved the highest TPR (0.61) and MCC (0.68) when we filtered the peaks on the basis of a CV threshold of 0.2. However, the enrichment was not as good as with the clustering by StoatyDive, where we achieved a mean LFC of 3.4 in the second cluster that was higher than the resulting mean LFC of 1.6 (median *P*-value of 0.108) with the CV cut-off of 0.2. Based on these different sets, we observed that the CV was sometimes lower in the main or second cluster. Together with the previous results, we concluded that broad and sharp peaks play an equally important role for SLBP.

### Comparison to existing tools

**Figure 5:**
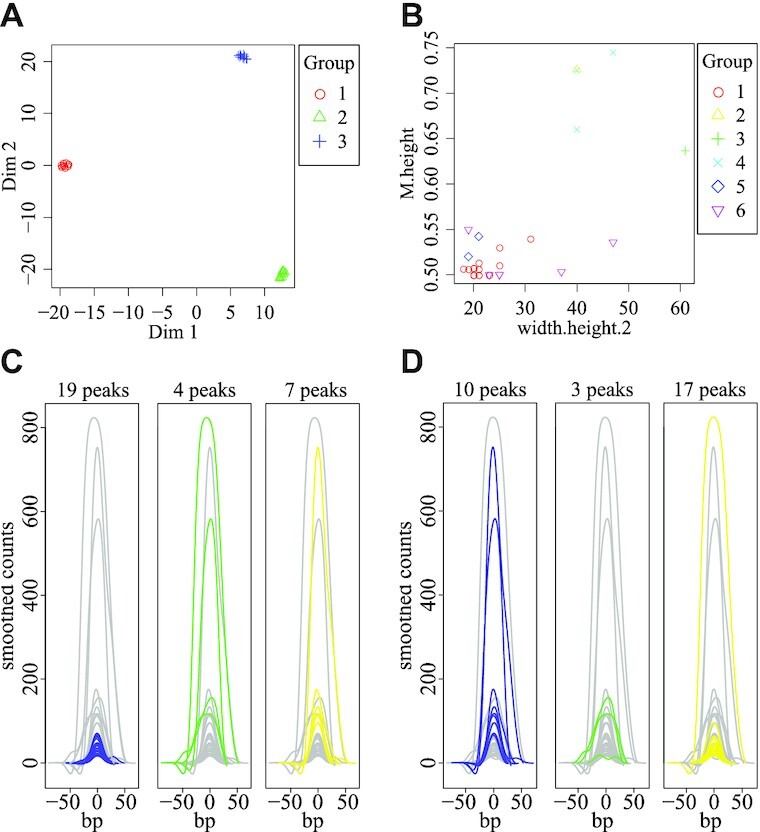
Peak shape clustering with StoatyDive, SIC-ChIP [18], and FunChIP [17] on a set of selected peaks from 3 different peak shapes of the second replicate of SLBP. (A) StoatyDive successfully identified the 3 distinct peak shape groups with 10 peaks each, but a few peaks were sorted incorrectly (ACC = 0.87). (B) From the 5 shape indices of SIC-ChIP (see Supplementary Fig. S5), we picked 1 scatter plot (B) with the highest explained variance (*w*_*h*/2_ vs *M*/*h*) to show the clustering of SIC-ChIP. FunChIP was only able to identify 3 distinct clusters (ACC = 0.6). The (C) smoothed and (D) smoothed and scaled profiles were clustered mostly on the intensity of the summit. The colored lines show the profiles of the related cluster, whereas the grey lines correspond to all other profiles.

To further validate StoatyDive, we applied 2 other peak shape clustering tools, namely, FunChIP ([17]; version 0.99.4) and SIC-ChIP ([18]; current release), to the second replicate of SLBP. To have a better ground truth, we took 10 peaks of 3 different peak shape groups (broad, sharp, and plateau) to define a test set with 3 distinct peak shapes from real CLIP-Seq data (in total 30 peaks). We defined peaks as sharp (CV >1.0) and broad (CV <0.05) based on the calculated CV of StoatyDive. We selected peaks shaped like plateaus by inspecting them in a genome browser. We strictly used the output of the tested tools. Both tools were designed and tested for ChIP data. A peak shape clustering had not thus far been done for CLIP data and a specific tool for that data type did not exist to our knowledge. We applied SIC-ChIP with *N* = 10 and toll = 10 (the default parameter set resulted in errors) and ran FunChIP according to the manual in Bioconductor with the smoothing parameter lambda = 10^3^. StoatyDive classified most peaks correctly into the 3 peak shape groups (Fig. 5A, accuracy [ACC] = 0.87). It sorted 4 peaks incorrectly that came from the broad and sharp peak groups. Sharp and broad peaks are harder to cluster, and a second factor such as the CV helps to give a final assessment regarding the shape of the peak. SIC-ChIP identified up to 6 different peak shapes (Fig. 5B), whereas FunChIP found 3 (Fig. 5C and D, ACC = 0.6). Furthermore, SIC-ChIP as well as FunChIP had clusters that are mixed and not as well separated as with StoatyDive. SIC-ChIP’s predefined shape indices were not enough to separate the peak shape profiles properly, as shown for 1 scatter plot (Fig. 5B) with the highest explained variance (cluster separation). In turn, FunChIP performed slightly better, finding profiles with different summit intensities. However, for the smoothed (Fig. 5C) as well as the smoothed and scaled profiles (Fig. 5D) the clusters included a lot of profiles with different shapes. For example, cluster 2 and cluster 3 of the smoothed profiles seemed very similar. Thus, FunChIP’s approach to use the whole profile without any predefined features or dimensional reduction was also not enough to separate the peak shapes in the same way as with StoatyDive.

### Investigation of eCLIP protein profiles

We further investigated the peak shapes of several proteins from the study of Van Nostrand et al. [5], namely, CPSF6, CSTF2T, EWSR1, LARP7, RBM22, SAFB2, SLBP, SLTM, TAF15, TRA2A, U2AF1, HNRNPA1, IGF2BP1, IGF2BP2, NONO, SRSF1, TARDBP, HNRNPM, U2AF2, and PTBP1. We took the robust peaks (peak-calling, IDR, signal normalization) and the bam files, which were used for the peak-calling, from each protein from the ENCODE database. We chose the data from the eCLIP experiment on K562 and focused on proteins for which the biological and molecular function and the number of RRMs are clearly listed on UniProt. Thus, we wanted to investigate whether the number of RRMs or the function of the protein by any means affected the shape of the peak profiles and consequently led to more or fewer broader peaks. We therefore took the files from ENCODE and merged both bam files (replicates) for the coverage. We then used StoatyDive with --peak_correction --scale_max 10 --border_penalty --sm --peak_length 77 -k 3. All peaks were therefore extended or shrunk to a length of 77 nucleotides. This was based on the observation that the third quartile of all peaks from all proteins was 77 nucleotides long (see Supplementary Fig. S4). In addition, StoatyDive achieved for the SLBP data a better TPR and MCC with a peak size of 70 and a CV threshold of 0.2 (see Supplementary Table S1). The results of SLBP showed that it may be wise to combine the clustering and the CV threshold to assess the profile landscape of other proteins. We therefore defined a peak as sharp if it had a CV >0.2 and it fell into a cluster that was generally sharper. A cluster was declared as sharp if the median CV of the cluster was bigger than the median CV of the whole peak set. All other peaks were classified as broad.

**Figure 6:**
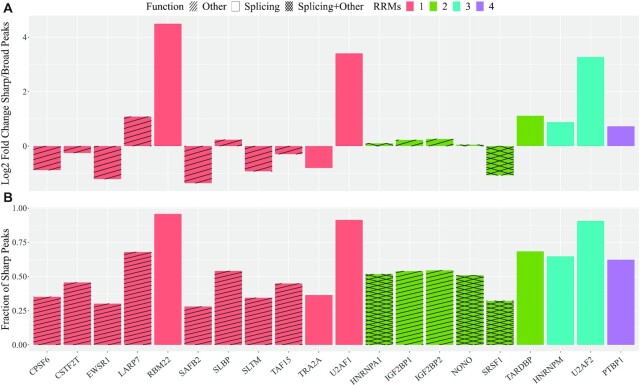
(A) Log_2_ fold change of the number of sharp peaks vs the number of broad peaks; (B) fraction of sharp peaks. UniProt lists the proteins CPSF6, CSTF2T, EWSR1, LARP7, RBM22, SAFB2, SLBP, SLTM, TAF15, TRA2A, and U2AF1 with 1 RRM and the proteins HNRNPA1, IGF2BP1, IGF2BP2, NONO, SRSF1, TARDBP, HNRNPM, U2AF2, and PTBP1 with ≥2 RRMs. Proteins with >1 RRM tend to have sharper-shaped peaks but also equally have broader peaks. The proteins RBM22, U2AF1, TARDBP, HNRNPM, U2AF2, and PTBP1 had a higher number of sharper peaks and all of them are involved in RNA splicing (colored bars). Other proteins with different functions (stripes) often have less sharp peaks.

The proteins LARP7, RBM22, SLBP, U2AF1, IGF2BP2, NONO, TARDBP, HNRNPM, U2AF2, and PTBP1 had a higher number of sharper-shaped peaks, whereas the rest of the proteins had a higher number of broader-shaped peaks relative to the other shape (Fig. 6A). Protein IGF2BP1 was almost half sharp and half broad peaks. UniProt lists the proteins CPSF6, CSTF2T, EWSR1, LARP7, RBM22, SAFB2, SLBP, SLTM, TAF15, TRA2A, and U2AF1 with 1 RRM and the proteins HNRNPA1, IGF2BP1, IGF2BP2, NONO, SRSF1, TARDBP, HNRNPM, U2AF2, and PTBP1 with ≥2 RRMs.

We could observe a trend between the number of RRMs and the number of sharper-shaped peaks (Fig. 6A). From 11 proteins with 1 RRM just 4 had sharper peaks than broader-shaped peaks and 8 of 9 proteins with ≥2 RRMs had sharper-shaped peaks. Furthermore, the proteins HNRNPM, U2AF2, and PTBP1 all have >2 RRMs (3, 3, and 4, respectively); thus it might be possible that an increasing number of RRMs results in sharper peaks. Figure 6B shows more clearly that proteins with >1 RRM tend to have sharper peaks (2-sided Wilcoxon test *P*-value ≈0.046). It is possible that RNA-protein interactions become more specific with >1 RRM and so the separation between more specific and unspecific binding sites is stricter.

Another observation was that shapes of splicing factors (RBM22, U2AF1, TARDBP, HNRNPM, U2AF2, and PTBP1) tend to be sharper than broader (Fig. 6A). Yet, proteins, such as SRSF1, had broader peaks and are also involved in splicing. On the other hand, proteins not involved in splicing such as EWSR1, SAFB2, SLTM, and TAF15 clearly showed more broader-shaped peaks. The proteins SLBP, HNRNPA1, IGF2BP1, IGF2BP2, and NONO almost had an equal number of sharper or broader-shaped peaks, and all these proteins have multiple functions, contributing to ≥2 biological processes, such as transport and translation in the case of SLBP [6]. SRSF1 is also a multi-functional protein. Perhaps that is the reason why it has broader peaks even though it is a splicing factor.

We also checked whether our result that RBPs involved in splicing have sharper peaks can have technical reasons. In this case, a splicing-related protein could have more peaks that are split over 2 exons, which are detected by the peak caller as 2 separate but sharp peaks (split peak). So we investigated the number of peaks that fall into introns (90% overlap) for the proteins that are involved in the splicing process. We used BEDTools set to a strict overlap (intersect -u -s -f 0.9) to investigate potential split peaks. The proteins PTBP1 (≈85%), RBM22 (≈62%), TARDBP (≈79%), and HNRNPM (≈87%) had >50% of peaks in introns, which deflected the assumption of split peaks. However, the proteins U2AF1 (≈17%) and U2AF2 (≈15%) had more peaks in exon regions, where the possibility of split peaks might still occur. It is important to note that this does not mean that the aforementioned proteins bind generally more introns or exons. Because there exists no tool that can correct for split peaks, a further analysis for these proteins was required. We checked for potential split peaks by extending the peaks that fall completely into exons by 5 nucleotides to each side. Next, we intersected those extended peaks with introns to see whether they are close to the exon boundaries. We found for U2AF1 only 27 peaks (0.72%) and for U2AF2 5 peaks (0.40%) that are potential split peaks, again deflecting the assumption of a technical artifact in the peak set of splicing factors. Thus, the sharpness of the peaks of U2AF1 and U2AF2 was potentially not the result of the peak-calling.

### Potential implications

StoatyDive is a powerful tool that can evaluate and classify peak profiles. It can be used in any sequencing data analysis that involves the prediction of binding sites such as CLIP-Seq, or ChIP-Seq. Within this work, we provided an example for SLBP to show the usability of StoatyDive. First, it is possible to assess the quality of an experiment such as CLIP. The CV is just 1 quality factor, and we recommend testing other features as well, such as the read coverage correlation. Second, StoatyDive assists in the evaluation of the binding specificity of the protein. The normalized CV distribution produced by StoatyDive provides valuable information for the user. A protein that binds very specifically will have a distribution concentrated around a normalized CV of 1. A protein with a lot of unspecific bindings will have a normalized CV distribution ≈0. Third, StoatyDive helps to filter for specific and unspecific binding sites to investigate whether the protein has multiple protein domains that have different binding mechanisms. A finer distinction can be made with the classification mode of StoatyDive. This helps to identify peak profiles with a specific shape and filter them on the basis of the corresponding biological question and function of the protein. Fourth, the results of StoatyDive can be used to validate a peak caller (e.g., PureCLIP); i.e., one can assess how many false-positive results are in the peak sets based on the shape. Use of a different peak caller might result in disparate peak sets and consequently different peak profile shapes.

We could show with StoatyDive that proteins with a higher number of RRMs tend to have sharper binding profiles. However, we have not taken any other RNA binding domains into account apart from RRMs and we would need to investigate more proteins to be confident about this trend. However, we could demonstrate that splicing factors tend to have more sharper peaks in comparison to proteins with other functions.

StoatyDive is a very powerful, well-documented, and easy-to-apply tool that refines binding site detection in data analysis such as CLIP-Seq. Nevertheless, StoatyDive is a very general tool. In the future it is worth investigating whether StoatyDive can be used with different types of peak-calling outputs and data types of sequencing data (e.g., ChIP-Seq, ATAC-Seq, Ribo-Seq). It serves as a quality control and filtering step to select specific binding profiles, which therefore allows improvement of other binding site prediction tools such as DeepBind [19], or any other subsequent analysis tasks, to increase the accuracy for the prediction.

## Methods

### Peak correction, extension, and coverage calculation

**Figure 7:**
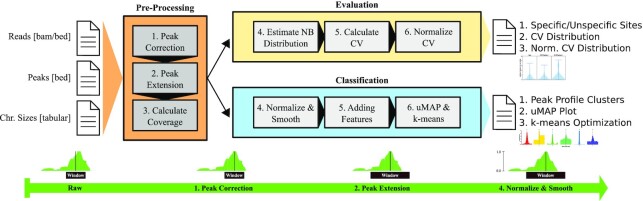
Overview of the StoatyDive pipeline. It consist of 2 major modules, namely, the evaluation and the classification of peak profiles. The user has to provide reads (or events), peaks, and a chromosome size file. StoatyDive then shifts the peaks to their correct center (peak correction), extends the peaks to a common length (maximal peak length of peak set or user-defined value), and calculates the coverage with BEDTools [10]. The peak correction can be turned off. In the evaluation, StoatyDive then estimates the read coverage as a negative binomial. From the hyperparameters it calculates the coefficient of variation (CV) and normalizes it (Equations 1 and 2). The normalized CV can then be used to divide the peaks into specific and unspecific sites. Furthermore, the CV distribution acts as a quality control between control and signal experiments. In the classification, StoatyDive first normalizes the peak profiles to remove the intensity as a feature. Then it smooths the profiles to support the data assumptions of uMAP [20] and to remove some noise. After that, it adds curve-specific features to the data. The higher dimension of the data is then reduced with uMAP. StoatyDive then clusters the new data with *k*-means [21]. The user then obtains several plots and a table to investigate the different peak profile clusters.

StoatyDive was implemented in Python (≥3.6) and R (≥3.4.4). The tool needs 3 files: the predicted binding regions of a peak-calling algorithm in bed6 format, a bam or bed file that was used for the peak-calling (experiment or control), and a tabular file of the chromosome size of the reference genome (Fig. 7).

First, StoatyDive checks whether a peak profile needs to be centered (peak correction). In the default mode, the profiles are centered by a convolution with a standard normal distribution. The maximum value of the convolution gives the nucleotide shift of the peak profile to center the peaks. So the window with the peak length is shifted to the center of the peak (Fig. 7 Step 1). With this approach we retain the context and take care of 2 problems. First, peak callers often produce peaks that are not correctly centered. Second, dimensionality reduction methods, such as uniform manifold approximation and projection for dimension reduction (uMAP [20]), are not translation invariant. Thus, 2 profiles with the same shape but in a different relative genomic position might end up in different locations in the new dimensional space.

After the peak correction, StoatyDive extends the peaks by default to the maximal peak length of the given peak set (Fig. 7 Step 2). This removes the peak length as a potential feature for the evaluation and classification. StoatyDive then calculates the read coverage (Fig. 7 Step 3) for each position inside a peak with the help of BEDTools (BEDTools, RRID:SCR_006646) [10].

### Evaluation of peak profiles

With the results of BEDTools, StoatyDive evaluates every peak *i* from the total set of *k* peaks. StoatyDive will estimate the read count for every peak as a negative binomial *X_i_* ∼ NB(*r_i_*, *p_i_*) with the hyperparameters *r_i_* (number of hits) and *p_i_* (probability of a hit). It then calculates the CV for every peak. A simple estimation of the variance is not enough because the profile depends on the read coverage. Thus, to be able to compare each peak profile we have to normalize for the expected number of reads to adjust the variance. So the CV for each peak,

(1) \begin{eqnarray*}
\mathrm{CV}_i = \sqrt{\frac{1-p_i}{r_i}}, \end{eqnarray*}

is calculated with the estimated hyperparameters. In the last step, StoatyDive normalizes the CV score by the maximum and minimum of all scores,

(2) \begin{eqnarray*}
\mathrm{CV}_i^{\prime } = \frac{\mathrm{CV}_i - \text{min}(\forall \mathrm{CV})}{\text{max}(\forall \mathrm{CV}) - \text{min}(\forall \mathrm{CV})}. \end{eqnarray*}

At the end, our defined CV score will be in the range CV_*i*_ = [0, ∞] and the normalized score, in the range $\mathrm{CV}_i^{\prime } = [0, 1]$, with a $\mathrm{CV}_i^{\prime }=0$ for a more unspecific binding and $\mathrm{CV}_i^{\prime }=1$ for a more specific one.

### Classification of peak profiles

StoatyDive classifies the peak profiles in an unsupervised manner using uMAP [20] and *k*-means clustering [21]. Yet before clustering, StoatyDive processes the peak profiles. First, the profiles are normalized on the basis of the individual maximum and minimum read count because we are only interested in the shape of the profiles and not in the absolute read counts (Fig. 7 Step 4). So assuming each peak *X_i_* has *x*_1_, *x*_2_, *x_j_*..., *x_n_* nucleotides, we normalized the peaks by $x_j = [x_j - \text{min}(X_i)]/[\text{max}(X_i) - \text{min}(X_i)]$. Second, the peak profiles are smoothed (Fig. 7 Step 4) with a spline regression [22]. The step reduces the noise for each profile and distributes the data more uniformly on the current manifold. The latter is important because it is the data assumption of uMAP. StoatyDive further adds curve-specific features to the processed peak profiles, including the number of maximal values, the area under the curve, and the arc length. StoatyDive applies uMAP to the final data with 5,000 epochs, 2 components (dim = 2), a minimum distance of 0.01, and a size of the local neighborhood of 5. The original and high-dimensional profiles are often hindered by the curse of dimensionality, which would lead to a higher number of individual clusters. The dimensional reduction was optimized with some test data comprising 4 different sets of distributions: a uniform distribution, a linear distribution, a unimodal Gaussian distribution, and a bimodal Gaussian distribution. Subsequently, StoatyDive applies *k*-means clustering to the new data with 100 initializations, and maximal 10,000 iterations. The number of clusters *k* is found by convergence of the total within-cluster sum of squares and checked with the Akaike information criterion [23]. We also tested other dimensionality reduction methods (see Supplementary Fig. S6) such as principal component analysis (PCA), a self-organizing map (SOM), and t-distributed stochastic neighbor embedding (t-SNE). However, none of them came close to the results of uMAP.

### Output of StoatyDive

For the peak evaluation, StoatyDive generates a plot of the CV (Equation 1) and normalized CV (Equation 2) distribution (Fig. 7). The user receives a first impression of the binding specificity of the protein of interest from the CV distribution. An unspecific binder has a CV distribution ≈0. A more specific binder has a CV distribution >0. The CV distribution can also be used as a quality control to compare control and signal experiments. A quality breach might have occurred if the distributions of the control and signal experiment look almost identical. A control experiment should normally have a CV distribution ≈0, with only a very few binding sites showing higher CVs. A CLIP experiment, on the other hand, should contain more peaks with higher CVs and thus have a CV distribution that significantly differs in comparison to the control (Wilcoxon *P*-value <0.05, see Supplementary Fig. S1).

The normalized CV distribution helps to evaluate the peaks based on the individual experiments. An empirical threshold is set at a CV of 0.2 (Equation 1), below which binding sites are deemed unspecific. The user can change the threshold. Keep in mind, the threshold for the normalized CV is relative in accordance with the individual experiment.

For the peak classification, StoatyDive generates a plot of the *k*-means optimization and a plot of the dimensional reduction with uMAP, which can be used to readjust the number of *k* clusters if this is necessary. The user also receives a set of example peak profiles and smoothed peak profiles of each cluster, which can be used to investigate the identified shapes. For a general trend, StoatyDive delivers average profiles for each cluster.

The final output of StoatyDive is a CV-sorted table of the whole peak set, from the highest to the lowest CV. Each peak is labeled with 0, for more specific binding sites, and 1, for more unspecific sites. The table also lists for each peak the cluster number (group number) of the peak profile shape.

### Important options of StoatyDive

The peak correction (Fig. 7 Step 1) can be turned off. The user can also change the translocation scheme of the peak profiles to shift them based on the maximal value (summit). The maximum translocation scheme is useful for nucleotide-specific events such as truncation events in the case of iCLIP data [24]. StoatyDive also has the option for a different CV score that penalizes peaks within broad plateaus. StoatyDive then adjusts the CV score of peaks that are covering a small appendage of a read stack. Furthermore, the user can provide a maximal score to StoatyDive to normalize the CV distribution (Equation 2). This option helps to compare the CV distribution between experiments in accordance with their disparate peak sizes and total amount of reads. StoatyDive also has a threshold for the normalized CV score to divide the peaks into more specific and more unspecific binding sites, which the user can change.

StoatyDive has 2 major parameters for the peak profile classification (Fig. 7 Step 6). First, the user can adjust the maximal amount of potential peak clusters identified by the *k*-means clustering. Yet, the final number of peak clusters will be optimized by StoatyDive. The parameter is an upper bound. However, the user has the option to force StoatyDive to use *k* specific clusters. The smoothing (Fig. 7 Step 4) of the peak profiles can also be adjusted by the user. The default was optimized with different test sets. Increasing the parameter (greater than default) might underfit the smoothing and thus lead to fewer peak clusters. A lower value (less than default) might overfit and so lead to more clusters. The smoothing can also be turned off, but it is recommended to turn it on.

## Availability of Supporting Source Code and Requirements

Project name: StoatyDive

Project home page: https://github.com/BackofenLab/StoatyDive

Conda: https://anaconda.org/bioconda/stoatydive

Operating system(s): Unix

biotools:StoatyDive

License: GPLv3

RRID:SCR_018796

## Data Availability

StoatyDive provides a small dataset for a test run, which can be found in the github repository. The whole eCLIP dataset used in this article, such as SLBP or RBFOX2, is listed in the supplementary material of the study by Van Nostrand et al. [5] and in the *GigaScience* Database [25].

## Additional Files

**Supplementary Figure S1**: CV distributions of all other proteins analyzed for Fig. 6 with 2-sided Wilcoxon test *P*-value, the number of uniquely mapped reads, and the mean CV for each replicate. The 2 replicates quite often have different CV distributions. Furthermore, we report a plot for the mean CV for the CLIP data in comparison to the size-matched input control of each protein. The control data tend to have a CV close to 0. The CV distributions between CLIP and control data always have a 2-sided Wilcoxon test *P*-value <0.05.

**Supplementary Figure S2**: We applied StoatyDive to the size-matched input control of the SLBP data [5]. StoatyDive found 4 different peak profile shapes, broad (cluster 1), plateau (cluster 2), sharp (cluster 3), and constant (cluster 4). The supplements also include the average profiles for Replicates 1 and 2 to show the overall trend of the clusters.

**Supplementary Figure S3**: Peak lengths of the peak set (ENCFF127WAK) for the second replicate of SLBP and peak lengths of all other proteins of the eCLIP data from the study by Van Nostrand et al. [5].

**Supplementary Figure S4**: All scatter plots from SIC-ChIP [18] for the artificial SLBP data.

**Supplementary Figure S5**: We tested different dimensional reduction methods such as PCA, SOM, and t-SNE on the CLIP data of SLBP. The PCA has no clear clusters for Replicate 2, which is similar for t-SNE on Replicates 1 and 2. Using an optimized SOM delivers a feature layer with a very high activated hidden unit for Replicate 2. It is hard to see any distinct clusters from the counts (activation) of each hidden unit. uMAP can clearly separate the data into more defined clusters. Furthermore, it is much easier to interpret the results of uMAP, whereas an artificial neural network, such as a SOM, generates a feature layer (hidden layer) that is hard to explain.

**Supplementary Table S1**. Mean CV, variance of the CV, mean log_2_ fold change (LFC) enrichment between the control and CLIP experiment, median *P*-value, true-positive rate (TPR), true-negative rate (TNR), accuracy (ACC), and Matthews correlation coefficient (MCC) for the analyzed peaks (all peaks) of Replicate 2 (ENCFF127WAK) from the study by Van Nostrand et al. [5]. Features are listed for the peak shape cluster with the highest number of peaks in histones (main cluster) and second highest number (second cluster), and for the peaks with a CV smaller or bigger than a threshold of 0.2, 0.5, and 0.8, using different peak lengths (30, 40, 70, and maximum peak length of 201 nucleotides). We achieved the best TPR, ACC, and MCC with a peak length of 70 and with a CV cut-off of 0.2.

## Abbreviations

ACC: accuracy; CLIP-Seq: cross-linking immunoprecipitation in combination with high-throughput sequencing; CV: coefficient of variation; IQR: interquartile range; LFC: log_2_ fold change; MCC: Matthews correlation coefficient; mRNA: messenger RNA; PCA: principal component analysis; RBP: RNA-binding protein; RRM: RNA recognition motif; SOM: self-organizing map; TPR: true-positive rate; TNR: true-negative rate; t-SNE: t-distributed stochastic neighbor embedding.

## Competing Interests

The authors declare that they have no competing interests.

## Funding

This study was funded by the Deutsche Forschungsgemeinschaft (DFG, German Research Foundation) grant 322977937/GRK2344 2017 MeInBio – BioInMe Research Training Group, and Germany’s Excellence Strategy (CIBSS - EXC-2189 - Project ID 390939984). The article processing charge was funded by the University of Freiburg in the funding programme Open Access Publishing.

## Authors' Contributions

F.H. performed the computational analysis and tool developement. R.B. initialized the project and supervised the research. Both authors wrote the manuscript. Both authors read and approved the final manuscript.

## Supplementary Material

giab045_GIGA-D-20-00218_Original_Submission

giab045_GIGA-D-20-00218_Revision_1

giab045_GIGA-D-20-00218_Revision_2

giab045_GIGA-D-20-00218_Revision_3

giab045_GIGA-D-20-00218_Revision_4

giab045_Response_to_Reviewer_Comments_Original_Submission

giab045_Response_to_Reviewer_Comments_Revision_1

giab045_Response_to_Reviewer_Comments_Revision_2

giab045_Response_to_Reviewer_Comments_Revision_3

giab045_Reviewer_1_Report_Original_Submission Eric Van Nostrand -- 8/22/2020 Reviewed

giab045_Reviewer_1_Report_Revision_1 Eric Van Nostrand -- 12/15/2020 Reviewed

giab045_Reviewer_1_Report_Revision_2 Eric Van Nostrand -- 3/16/2021 Reviewed

giab045_Reviewer_2_Report_Original_Submission Nejc Haberman -- 8/25/2020 Reviewed

giab045_Reviewer_2_Report_Revision_1 Nejc Haberman -- 12/15/2020 Reviewed

giab045_Reviewer_3_Report_Original_Submission William Lai -- 8/28/2020 Reviewed

giab045_Reviewer_3_Report_Revision_1 William Lai -- 12/16/2020 Reviewed

giab045_Supplemental_Files
