# Supplementary material for: StoatyDive: Evaluation and classification of peak profiles for sequencing data
Source: Gigascience. 2021 Jun 18;10(6):giab045. doi: 10.1093/gigascience/giab045 (PMC8212874; doi:10.1093/gigascience/giab045)
Supplement: giab045_GIGA-D-20-00218_Original_Submission [file giab045_giga-d-20-00218_original_submission.pdf]

GigaScience

StoatyDive: Evaluation and Classification of Peak Profiles for Sequencing Data

--Manuscript Draft--

**Manuscript Number:**	GIGA-D-20-00218
**Full Title:**	StoatyDive: Evaluation and Classification of Peak Profiles for Sequencing Data
**Article Type:**	Technical Note
**Funding Information:**	Deutsche Forschungsgemeinschaft (322977937/GRK2344 MeInBio) — Mr. Florian Heyl<br>Deutsche Forschungsgemeinschaft (390939984 EXC-2189) — Prof. Dr. Rolf Backofen
**Abstract:**	Background<br><br>The prediction of binding sites (peak calling) is a common task in the data analysis of methods such as crosslinking immunoprecipitation in combination with high-throughput sequencing (CLIP-Seq). The predicted binding sites are often further analyzed to predict sequence motifs or structure patterns. When looking at a typical result of such a high-throughput experiments, the obtained peak profiles differ largely on a genomic level. Thus, a tool is missing that evaluates and classifies the predicted peaks based on their shapes. We hereby present StoatyDive, a tool that can be used to filter for specific peak profile shapes of sequencing data such as CLIP.<br>Findings<br><br>With StoatyDive we are able to classify peak profile shapes from CLIP-seq data of the histone stem-loop-binding protein (SLBP). We compare the results to existing tools and show that StoatyDive finds more distinct peak shape clusters for CLIP data. Furthermore, we present StoatyDive's capabilities as a quality control tool and as a filter to pick different shapes based on biological or technical questions for other CLIP data from different RNA binding proteins with different biological functions and number of RNA recognition motifs. We finally show that proteins involved in splicing, such as RBM22 and U2AF1, have potentially more sharper shaped peaks than other RNA binding proteins.<br>Conclusion<br><br>StoatyDive finally fills the demand for a peak shape clustering tool for CLIP-Seq data that fine tunes downstream analysis steps such as structure or sequence motif predictions and that acts as a quality control.
**Corresponding Author:**	Florian Heyl<br><br>GERMANY
**Corresponding Author Secondary Information:**	
**Corresponding Author's Institution:**	
**Corresponding Author's Secondary Institution:**	
**First Author:**	Florian Heyl
**First Author Secondary Information:**	
**Order of Authors:**	Florian Heyl<br>Rolf Backofen
**Order of Authors Secondary Information:**	
**Additional Information:**	
**Question**	**Response**
Are you submitting this manuscript to a	No

Powered by Editorial Manager® and ProduXion Manager® from Aries Systems Corporation

special series or article collection?	
**Experimental design and statistics**<br><br>Full details of the experimental design and statistical methods used should be given in the Methods section, as detailed in our Minimum Standards Reporting Checklist. Information essential to interpreting the data presented should be made available in the figure legends.<br><br>Have you included all the information requested in your manuscript?	Yes
**Resources**<br><br>A description of all resources used, including antibodies, cell lines, animals and software tools, with enough information to allow them to be uniquely identified, should be included in the Methods section. Authors are strongly encouraged to cite Research Resource Identifiers (RRIDs) for antibodies, model organisms and tools, where possible.<br><br>Have you included the information requested as detailed in our Minimum Standards Reporting Checklist?	Yes
**Availability of data and materials**<br><br>All datasets and code on which the conclusions of the paper rely must be either included in your submission or deposited in publicly available repositories (where available and ethically appropriate), referencing such data using a unique identifier in the references and in the "Availability of Data and Materials" section of your manuscript.<br><br>Have you have met the above requirement as detailed in our Minimum Standards Reporting Checklist?	Yes

Powered by Editorial Manager® and ProduXion Manager® from Aries Systems Corporation

Powered by Editorial Manager® and ProduXion Manager® from Aries Systems Corporation

Manuscript Click here to access/download;Manuscript;main.pdf

OXFORD (GIGA)^n SCIENCE

GigaScience, 2017, 1–9

doi: xx.xxxx/xxxx
Manuscript in Preparation
Technical Note

TECHNICAL NOTE

StoatyDive: Evaluation and Classification of Peak Profiles for Sequencing Data

Florian Heyl[1,*] and Rolf Backofen[1,2,*]

[1]Bioinformatics Group, Department of Computer Science, University of Freiburg, Georges-Köhler-Allee 106, 79110 Freiburg, Germany and [2]Signalling Research Centres BIOSS and CIBSS, University of Freiburg, Schaenzlestr. 18, 79104 Freiburg, Germany

[*]heylf@informatik.uni-freiburg.de, backofen@informatik.uni-freiburg.de

Abstract

Background The prediction of binding sites (peak calling) is a common task in the data analysis of methods such as crosslinking immunoprecipitation in combination with high-throughput sequencing (CLIP-Seq). The predicted binding sites are often further analyzed to predict sequence motifs or structure patterns. When looking at a typical result of such a high-throughput experiments, the obtained peak profiles differ largely on a genomic level. Thus, a tool is missing that evaluates and classifies the predicted peaks based on their shapes. We hereby present StoatyDive, a tool that can be used to filter for specific peak profile shapes of sequencing data such as CLIP.
Findings With StoatyDive we are able to classify peak profile shapes from CLIP-seq data of the histone stem-loop-binding protein (SLBP). We compare the results to existing tools and show that StoatyDive finds more distinct peak shape clusters for CLIP data. Furthermore, we present StoatyDive's capabilities as a quality control tool and as a filter to pick different shapes based on biological or technical questions for other CLIP data from different RNA binding proteins with different biological functions and number of RNA recognition motifs. We finally show that proteins involved in splicing, such as RBM22 and U2AF1, have potentially more sharper shaped peaks than other RNA binding proteins.
Conclusion StoatyDive finally fills the demand for a peak shape clustering tool for CLIP-Seq data that fine tunes downstream analysis steps such as structure or sequence motif predictions and that acts as a quality control.

Key words: CLIP-Seq; Data Analysis; Peak Shape Clustering; RNA; Protein

Findings

Background

The biological function of a protein is determined by its interaction partners and the mode of interaction. Studying these interactions broadens our horizon about the cellular mechanisms such as alternative splicing and post-transcriptional regulation. Crosslinking immunoprecipitation in combination with high-throughput sequencing (CLIP-Seq) fathoms these interactions. CLIP-Seq investigates all interactions between an RNA binding protein (RBP) and its target RNAs [1]. CLIP-Seq thus scrutinizes the post-transcriptional regulation by RBPs. Prediction of binding regions (peak calling) is a crucial step in the data analysis of methods such as CLIP-Seq. Before the peak analysis there is typically no evaluation and classification of the peak characteristics. Yet, the obtained peak set might have different peak profiles that are worth to filter to refine a downstream analysis. The different peak shapes are the result of several biological and technical problems.

Many RBPs have several binding domains with different binding affinities, and are often part of protein complexes, leading to an intricate binding pattern. As described in a review by Jankowsky, Eckhard and Harris, Michael E [2], there are specific and unspecific binders. Examples for unspecific binders are often RBPs that need to bind many RNAs such as mRNA export factors [3]. Another example of common unspecific binders are RNA helicases. However, even more specific

Compiled on: July 23, 2020.
Draft manuscript prepared by the author.

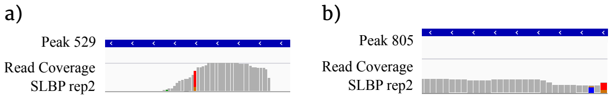

Figure 1. Example of different peak shapes. Looking at two peaks of a CLIP experiment for the protein SLBP, one can see peaks with drastically different peak profiles, pointing towards more specific (a) or unspecific (b) binding. Current analysis of CLIP binding sites is typically based on manual inspection of a few peaks. Thus a general tool is missing that allows to filter, cluster and quantify peak profiles and therefore refine downstream analysis tasks for data such as CLIP. StoatyDive assists to find and distinguish peaks like (a) and (b).

RBPs bind RNAs in large range of affinities, indicating that different binding sites vary in their binding specificity. While many factors, such as the affinity of an RBP for the binding site and the concentration of the protein and RNA, influence the binding specificity, it is likely that these factors are manifested in the CLIP binding profile landscape. At this point, however, no tool exists that can be used to study this possibility in more detail.

In addition, technical biases might change the peak profile landscape. Errors during the read library preparation might introduce unspecific bindings. Protocol biases, for example, PAR-CLIP biases that are introduced by endonuclease and photoactivatable nucleosides [4], might also affect the binding site predictions. On top, the peakcaller itself might generate specific peak profiles and false positives, which the user might not want to have in their data.

This leads to many questions in the data analysis of binding sites that can currently not be answered adequately. Examples are: Does my protein of interest bind generally more specific (Figure 1a) or more unspecific (Figure 1b)? Does my RBP of interest have more than one binding site? Does my experiment have some quality issues, meaning, does my reads come from unspecific bindings because of library preparation errors? Does my protocol generate biases? Do I have false positives in the set of predicted peaks from my peakcaller of choice?

We hereby present StoatyDive, a tool to evaluate and classify peak profiles to help to answer the aforementioned questions. StoatyDive uses the whole peak profiles as well as predefined features to do a peak shape clustering for sequencing data. In this paper, we will test StoatyDive on CLIP data of the eCLIP protocol from the histone stem-loop-binding protein (SLBP) from the study by Van Nostrand et al. [5]. SLBP has been reported to be a histone mRNA export and translation factor [6]. StoatyDive delivers several plots and a table to assess the different binding profiles of a protein. The tool assists to select specific and unspecific binding sites and to find similar shaped peak profiles. Thus, we try to refine the obtained peaks of the SLBP data to find more specific sites of SLBP. It also helps as a quality assessment to validate a CLIP-Seq or any other binding experiment. Later in the paper, we use StoatyDive to investigate the peak profile landscape of different RBPs with different biological functions and different number of RNA recognition motifs (RRM). StoatyDive comes with some test data and a quick installation guide.

Data Preparation of SLBP and Analysis

We used eCLIP data of the histone stem-loop-binding protein (SLBP; ENCSR483NOP; GSE91802; Van Nostrand et al. 5). The data comprised two CLIP replicates and one size-matched input control from immortalised myelogenous leukemia cells (K562). We processed the data with the snakemake pipeline SalamiSnake (https://github.com/BackofenLab/SalamiSnake, v0.0.1) for eCLIP data. SLBP has been reported to be cytoplasmic and be present in the nucleus [6]. Thus, we mapped the reads against hg38 genome with STAR [10], but also taking the transcriptome into account. We predicted potential binding sites of SLBP with PureCLIP [11], which we ran for each CLIP replicate separately, taking the input control into account. We extended the predicted binding regions by 20 nucleotides left and right because PureCLIP often underestimates the binding region. We further fused the predicted peaks from each CLIP replicate with bedtools [7] to get a robust set of predicted binding sites. We executed StoatyDive (v1.1.0 with umap v0.2.5.0) with the length normalization, the penalty for broader plateaus, and the peak profile smoothing. The complete call was: *StoatyDive.py -a peaks.bed -b reads.bam -c hg38.chrom.sizes.txt --peak_correction --scale_max 10 --border_penalty --sm*.

CV Results Reveal Low Reproducibility of Binding Sites

Both the CV distribution of the input control and replicate one of the SLBP data contained a lot of regions with a CV close to zero (Figure 2). In contrast, the CV distribution of replicate two was different since it had more peaks with a higher CV and thus more specific bindings such as peak 529 (Figure 1a, $CV \approx 5.3$). Yet, some potential binding sites were more unspecific with a CV closer to zero such as peak 805 (Figure 1b, $CV \approx 0.006$).

The CV distribution of the input control was expected, because ideally the control experiment contains no real binding events. But, the CV distribution of replicate one did not match the assumptions. The distribution was an indicator for a low reproducibility of the binding sites. This was also striking when we compared the distribution of replicate one and two. However, we have to stress out that we are unsure if this is the result of the specificity of SLBP or the different quality of replicate one and two. For a downstream analysis, for example the prediction of sequence motifs, it is worth to either exclude replicate one or to investigate, why the CV distribution of replicate one was very different to replicate two. Thus given the inspection of StoatyDive, the user can now decide to investigate the unspecific binding sites of replicate one and compare them with the input control or replicate two. This helps to assess if SLBP might have protein domains that bind to RNA in an unspecific manner. The user can also test if the unspecific peak 805 might be a false positive of PureCLIP.

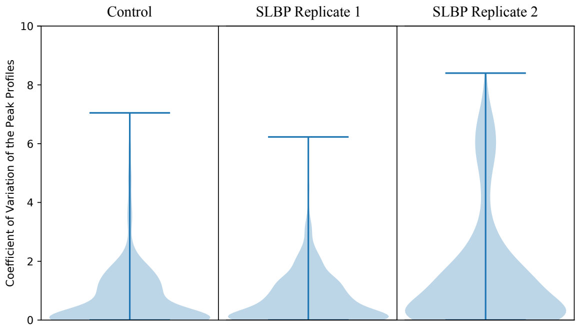

Figure 2. Evaluating the peak profile shapes is a quality control. (a) The distribution of the coefficient of variation of the peak profiles of the input control and replicate one of the SLBP CLIP-Seq experiment are very different to the distribution of replicate two. The user can subsequently inspect with StoatyDive more specific, sharp profiles (Figure 1a) and more unspecific, broader profiles (Figure 1b). Perhaps only one of the replicates is informative for specific patterns.

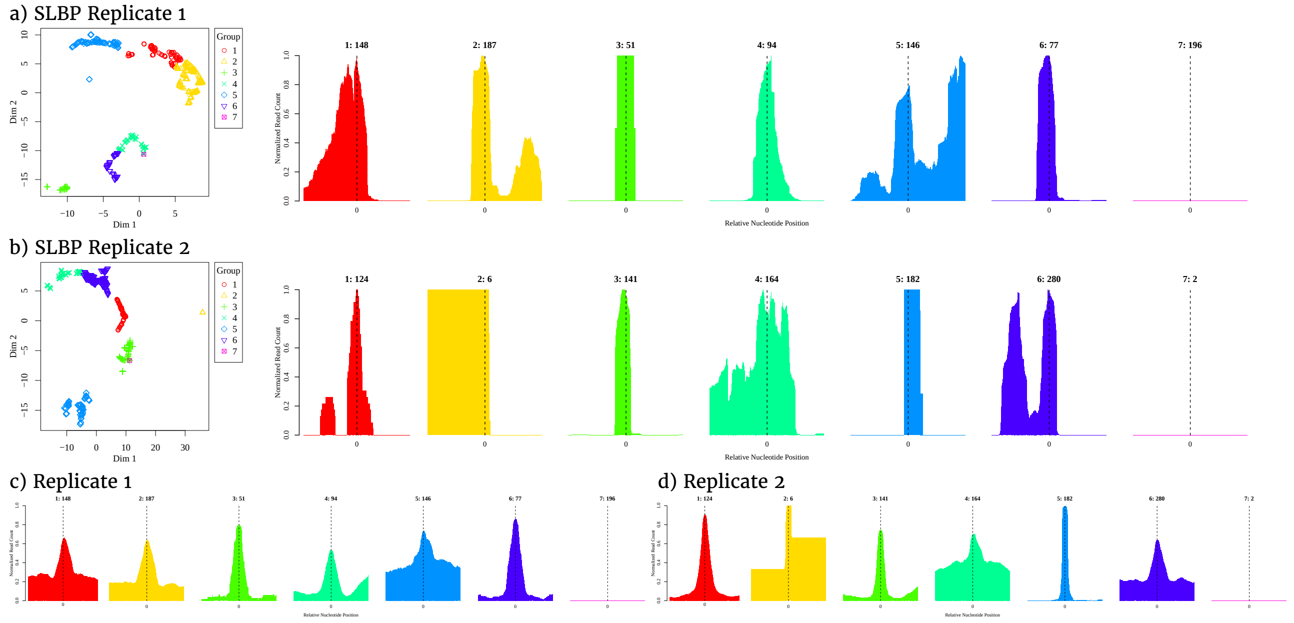

Figure 3. We applied StoatyDive to the SLBP data [5]. StoatyDive has found seven different peak profile shapes in the data of replicate one a1–7 and replicate two b1–7 of SLBP. We present one example profile for each cluster with the number of peaks in the title. The first plot shows the peak profiles in the lower dimension, when we applied uMAP. For replicate one, shape one was a broad peak profile. Shape two was a thin, spiky mountain, but with additional peaks surrounding it. Shape three was a flat plateau. Shape four was a mountain broader than shape six but thinner than shape one. Shape five was a very broad profile (cordillera). Shape six was a very thin and specific mountain. Shape seven was a constant profile. For replicate two, shape one was a small steep mountain with some small peaks surrounding it. Shape two was a very broad plateau. Shape three was a very thin and specific mountain such as peak 529 (Figure 1a). Shape four was a very broad profile (cordillera) such as peak 805 (Figure 1b). Shape five was a clear, unvarying plateau. Shape six was a broad profile with two or more clear peaks in close proximity. Shape seven was a constant profile. Average profiles for replicate (c) one and (d) two represented the overall trend of the peak shape clusters.

Seven Different Peak Shapes in the SLBP Data

For a more detailed analysis, we classified the peaks of replicate one and two with the help of StoatyDive (Figure 3). StoatyDive has found seven distinguishable peak profiles for replicate one and two. Because we assumed more unspecific sites in replicate one, we looked deeper into the profiles of replicate two (Figure 3b). Cluster two and five were clearly disparate. Groups one, three, four and six were much closer together than group two and five. Especially cluster four and six were similar. All four groups had profiles with mountain-like shapes. In contrast, cluster two and five are plateau shaped profiles. The mountains in the profiles became more broader and fuzzier from cluster three to one and then to six and four. The average, centered profiles (Figure 3c and d) reflected the trend of the ensemble of the individual profiles in each cluster (Figure 3 b1–7). To return to our initial examples (Figure 1), peak profile 529 was classified by StoatyDive as a small, centered mountain (Figure 3 b3), whereas peak profile 805 was a very broad profile (Figure 3 b4).

It is to mention that the number of clusters depend on the optimization of StoatyDive, or the user defined value. Other proteins, experimental conditions, and methods might have different peak profile groups. Even in our example it is worth to investigate if cluster four and six of replicate two can be separated more distinctly. At this point, one could run StoatyDive again, but only with the peaks of cluster four and six.

The high distance between cluster two and five might have been the result of the difference between the profile borders (compare Figure 3 b2 and b5). Where cluster two had profiles with lots of values in the left or right side of the peak profile, cluster five has occupied the center of the peak profile.

A broader and fuzzier profile might not necessarily mean that it was an unspecific site. Perhaps some of them were just a collection of several specific peak profiles that were merged together. This could have happened because of the peakcaller model or the peak correction and extension of StoatyDive. It is worth to take these profiles and reduce the extension, and in addition run a peak deconvolution.

It is important to note that peaks shaped like plateaus might be false positives. These peaks most likely corresponded to PCR duplicates that were not real binding sites. We have removed PCR duplicates during the preprocessing of the read library, but some duplicates might be still in the data. Sequencing errors in the unique molecular identifiers (UMI) are a common reason.

SLBP has been reported as an mRNA export and translation factor [6]. Thus, it is worth to investigate if peaks like 529 are more informative for a translation factor than peaks like 805. That is to say, 529 might be more suited for sequence and structure predictions than peak 805. Therefore, a deeper inspection of group one, three, four and six seems promising.

Information from Peak Profile Shapes

We made the assumption that replicate one might have more unspecific and less distinguishable profiles than replicate two based on the different CV distributions (Figure 2). Thus, we counted the number of peaks in each cluster for replicate one and two (Table 1). From 899 peaks, in replicate one we had $\approx$ 19% peaks being a sharp mountain shape (Figure 3 a4 and a6), $\approx$ 53% being a broader mountain (Figure 3 a1, a2 and a5), $\approx$ 6% peaks with plateaus (Figure 3 a3), and $\approx$ 22% constant shaped peaks (Figure 3 a7). Replicate two, on the other hand, had $\approx$ 29% sharp mountain shaped peak profiles (see Figure 3 b1 and b3), so 94 more than replicate one. This corroborated the assumption that replicate one had more broader and unspecific sites. Thus, replicate two had only $\approx$ 49% broad peak profiles (Figure 3 b4 and b6), and only 2 constant peak profiles (Figure 3 b7). Yet, replicate two had $\approx$ 21% peaks with plateaus (Figure 3 b2 and b5).

We further investigated the biological function of differ-

Table 1. Number of peaks of SLBP for different shape groups.

Replicate	Total	Sharp	Broad	Plateau	Constant
1	899	171	481	51	196
2	899	265	444	188	2
	Peak Summits in Histone mRNAs				
1	116	22	86	6	2
2	118	42	71	5	0

ent peak profiles of replicate two. Since SLBP targets histone mRNA [6], we intersected known annotated mRNAs of histones (hg38, Ensembl) with the summit of the different peak profiles (Table 1). From the 899 peaks, only ≈ 13% of replicate two overlapped with mRNAs of histones. Yet, of these 118 peaks almost all came from group one, three, four, and six. These groups were either spiky, or broader mountain shaped peak profiles. Only 5 peaks intersected with histone mRNAs that had a profile shaped like a plateau (Figure 3 b5). This endorsed the assumption that peak profiles shaped like plateaus were mainly PCR duplicates that were less informative. The observation also suggested that broader profiles were still informative, because some of them overlapped with histone mRNAs. We looked further and found sharper peaks located on stem loops targeted by SLBP such as RNU7-1 RNA (U7 small nuclear 1) or RNU6-2 RNA (U6 small nuclear 2), which got a CV of 3.9 (3.2, respectively) and was classified into the peak profile cluster 3 (cluster 1, respectively) of replicate two.

Consequently, we used MEME-ChIP [12] to analyze the sequence motifs of the different peak shape groups of the second replicate of SLBP. We found for the plateau peaks only two significantly enriched motifs (Table 2), whereas the sharper peaks had three. Yet, both the plateaus and the sharper peaks had two similar sequences motifs. Both motifs *(G)GCUCUUU(U)* and *(CA)GAGCCA(C)* were higher enriched in the sharper shaped peaks, which advocated the assumption. On the other hand, we found more than ten enriched motifs for the broader shaped peaks. The motifs of that peak set were very different to the motifs of the plateaus and sharper shaped peaks. Even the first three significantly enriched motifs had more noise and consequently were less enriched than the motifs of the other two peak shape groups. The E-values of all motifs were also more than a thousand times higher for broader peaks than for sharper peaks. Peaks shaped like plateaus were slightly more significant than broader peaks.

On further inspection of those peak motifs in histone mRNAs, we found that the three motifs of the sharper shape peaks covered ten more histone mRNAs (49 in total) than the broader shaped peaks (39 in total). This endorsed the observation that broader peaks encompassed more noise. For example, the second motif of the broad peaks *CA(A/C)CAAG* came close to the third sequence motif, with the sequence *A(C/A)CCAAAG*, of the sharper shape peak group. The second motif had the highest occurrence in histone mRNAs for the broader peaks. Thus, the broader peak set might include true binding sites but with some higher additional noise. Furthermore, we already showed that the plateau group might also hold some peaks that are true binding sites (Table 1), which was confirmed by the similar sequence motifs to the sharper peak set. We could confirm that the two sequence motifs that are present in plateaus were also present in histone mRNAs (Table 1). All in all, the sequence motifs analysis showed how different the outcome of subsequent tasks can be for different peak shape groups.

We investigated also the sequence motifs of sharp and broad peak profiles of the protein RBFOX2 (eCLIP data from the study by Van Nostrand et al. [5]), because it has the conserved sequence motif *TGCATG*, which is enriched in the RBPFOX2's binding sites [13, 14, 15]. We have found the conserved motif only in sharp peaks (Table 3), but the broader profiles also had an enriched motif. It is worth to investigate if RBFOX2 has some unspecific binding preferences with that specific motif. The results for RBFOX2 again demonstrated that different peak shape groups result in different sequence motifs.

Table 2. First three MEME-ChIP motifs for the different peak shape groups of SLBP with the E-value, the portion of sequences that have the motif, and the number of peaks that have the motif and which also intersect with histone mRNAs.

Shape	Motif 1	Motif 2	Motif 3
Broad	3.5e-4; 5.85%<br>13<br>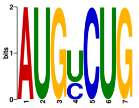	1.5e-3; 5.40%<br>18<br>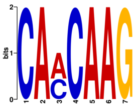	1.5e-3; 5.40%<br>8<br>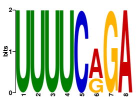
Sharp	5.7e-12; 20.00%<br>23<br>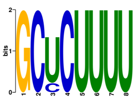	8.6e-9; 18.87%<br>17<br>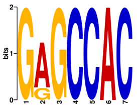	1.0e-3; 9.06%<br>9<br>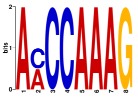
Plateau	1.6e-5; 15.42%<br>3<br>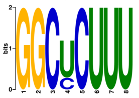	1.6e-3; 12.23%<br>2<br>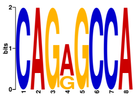	

Table 3. First three MEME-ChIP motifs for the different peak shape groups of RBFOX2 with the E-value.

Shape	Motif 1	Motif 2	Motif 3
Broad	2.5e-002<br>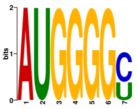		
Sharp	2.8e-027<br>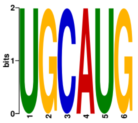	4.6e-003<br>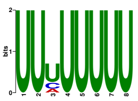	5.2e-003<br>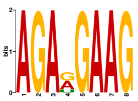

Comparison to Existing Tools

To further validate StoatyDive, we applied two other peak shape clustering tools, namely FunChIP (Parodi et al. 17; version 0.99.4) and SIC-ChIP (Cremona et al. 16; current release), to the second replicate of SLBP. To have a better ground truth, we took ten peaks of three different peak shape groups (broad, sharp, and plateau) to define a test set with three distinct peak shapes from real CLIP-Seq data (in total 30 peaks). We strictly used the output of the tested tools. Both tools were designed and tested for ChIP data. A peak shape clustering was so far not done for CLIP data and a specific tool for that data type did not exist to the best of our knowledge. We applied SIC-ChIP with $N = 10$ and $toll = 50$ and ran FunChIP according to the manual in Bioconductor with the smoothing parameter $lambda = 10^3$. StoatyDive classified all peaks correctly into the three peak shape groups (Figure 4a). SIC-ChIP identified up to six different peak shapes (Figure 4b), whereas FunChIP found three (Figure 4c and d). Furthermore, SIC-ChIP as well as FunChIP had clusters that are mixed and not as well separated as with StoatyDive. SIC-ChIP's predefined shape indices

were not enough to separate the peak shape profiles properly, as shown for one scatter plot (Figure 4b) with the highest explained variance (cluster separation). In turn, FunChIP performed slightly better, finding profiles with different summit intensities. However, for the smoothed (Figure 4c) as well as the smoothed and scaled profiles (Figure 4d) the clusters included a lot of profiles with different shapes. For example, cluster two and cluster three of the smoothed profiles seemed very similar. Thus, FunChIP's approach to use the whole profile without any predefined features or dimensional reduction was also not enough to separate the peak shapes in the same way as with StoatyDive.

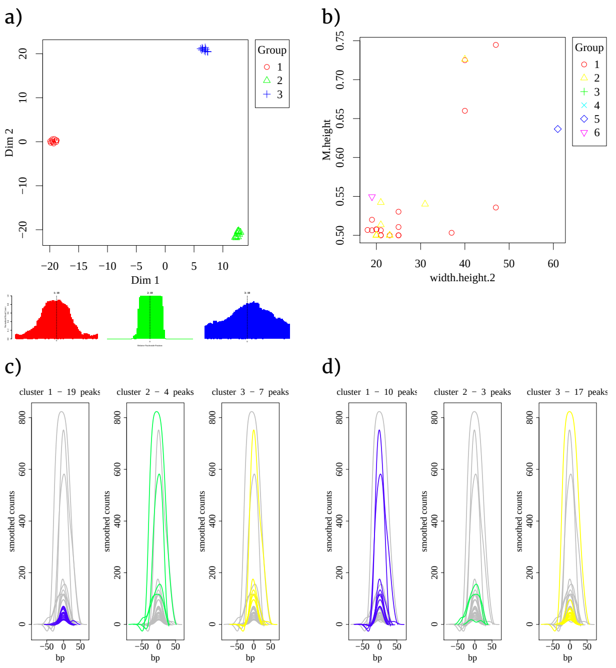

Figure 4. Peak shape clustering with StoatyDive, SIC-ChIP [16] and FunChIP [17] on a set of selected peaks from three different peak shapes of the second replicate of SLBP. (a) StoatyDive successfully identified the three distinct peak shape groups with ten peaks each. (b) From the five shape indices of SIC-ChIP, we picked one scatter plot (b) with the highest explained variance ($w_{h/2}$ vs $\frac{M}{h}$) to show the clustering of SIC-ChIP. The tool found six clusters, which overlapped. The five indices were not enough to differentiate the data. FunChIP was only able to identify three distinct clusters. The (c) smoothed and (d) smoothed and scaled profiles were clustered mostly on the intensity of the summit. Different shapes (broader or sharper peaks) were hard to distinguish.

Investigation of eCLIP Protein Profiles

We further investigated the peak shapes of several proteins from the study of Van Nostrand et al. [5], namely: CPSF6, CSTF2T, EWSR1, LARP7, RBM22, SAFB2, SLBP, SLTM, TAF15, TRA2A, U2AF1, HNRNPA1, IGF2BP1, IGF2BP2, NONO, SRSF1, TARDBP, HNRNPM, U2AF2, PTBP1. We took the robust peaks (peak calling, IDR, signal normalization) and the bam files, which were used for the peak calling, from each protein from the ENCODE database. We chose the data from the eCLIP experiment on K562 and focused on proteins where the biological and molecular function and the number of RRMs are clearly listed on UniProt. Thus, we wanted to investigate if the number of RRMs or the function of the protein by any means affected the shape of the peak profiles and consequently led to more or less broader peaks. We therefore took the files from ENCODE and merged both bam files (replicates) for the coverage. We then deconvoluted the peak profiles that had a length over 100 nucleotides, because StoatyDive depends on the peak length for the peak correction and longer peaks might disturb the overall peak profile set. Thus, simply taking the maximal peak length of the peak set might result in the extension of all other peaks to an inappropriate length. That is to say, some profiles (e.g., peaks with 300 nucleotides) might be an ensemble of several small overlapping peaks, which can be broken apart with a peak deconvolution. We then used StoatyDive with *--peak_correction --scale_max 10 --border_penalty --sm --peak_length 77 -k 3*. All peaks were therefore extended or shrank to a length of 77 nucleotides. This was based on the observation that the third quartile of all peaks (from all proteins) was 77 nucleotides long.

The proteins LARP7, RBM22, SLBP, U2AF1, IGF2BP2, NONO, TARDBP, HNRNPM, U2AF2 and PTBP1 had a higher number of sharper shaped peaks, whereas the rest of the proteins had a higher number of broader shaped peaks. Protein IGF2BP1 was almost half sharp and half broad peaks. UniProt lists the proteins CPSF6, CSTF2T, EWSR1, LARP7, RBM22, SAFB2, SLBP, SLTM, TAF15, TRA2A, U2AF1 with one RRM and the proteins HNRNPA1, IGF2BP1, IGF2BP2, NONO, SRSF1, TARDBP, HNRNPM, U2AF2, PTBP1 with at least two RRMs (Figure 5).

A clear correlation between the number of RRMs and the number of sharper shaped peaks was not observable. Yet, from eleven proteins with one RRM just four had more sharper peaks than broader shaped peaks. However, five out of nine proteins with at least two RRMs had sharper shaped peaks. Furthermore, the proteins HNRNPM, U2AF2, and PTBP1 have all more than two RRMs (3, 3, and 4, respectively), which led to the assumption that an increasing number of RRMs results in more sharper peaks. A possible hypothesis is that the interaction of RNA-protein becomes more specific with an increase number of RRMs. At this point, we had not taken any other RNA binding domains into account apart from RRMs.

Another observation was that the proteins RBM22, U2AF1, TARDBP, HNRNPM, U2AF2, and PTBP1 are involved in the splicing process and all these proteins had more sharper shaped peaks. Yet, the proteins TRA2A, and SRSF1 had more broader peaks and are also involved in splicing. On the other hand, proteins not involved in splicing such as EWSR1, SAFB2, SLTM, and TAF15 clearly showed more broader shaped peaks. The proteins SLBP, HNRNPA1, IGF2BP1, IGF2BP2, and NONO almost had an equal number of sharper or broader shaped peaks, and all these proteins have multiple functions, contributing to at least two biological processes such as transport and translation in the case of SLBP [6].

A combination of both factors, the number of RRMs and the biological function was not observable. The protein SRSF1 had more than one RRM such as TARDBP and both proteins were involved in the splicing process. Nevertheless, different factors on a biological and even technical level might still play a role for the peak shapes. For example, the number of broader and sharper shaped peaks of the protein SLBP was different from the previous results (Table 1). This can be the effect of a different peakcaller (the study of Van Nostrand et al. [5] used CLIPper) or the different data processing steps. We also checked whether our result that RBPs involved in splicing have sharper peaks can have technical reasons. In this case, a splicing related protein could have more peaks that are split over two exons, which are detected by the peakcaller as two separate but sharp peaks (split peak). So we investigated the number of peaks that fall into introns (90% overlap) for the proteins that are involved in the splicing process. The proteins PTBP1 ($\approx$ 85%), RBM22 ($\approx$ 62%), TARDBP ($\approx$ 79%), and HNRNPM ($\approx$ 87%) had more than 50% of peaks in introns, which deflected the assumption of split peaks. However, the proteins U2AF1 ($\approx$ 17%), and U2AF2 ($\approx$ 15%) had more peaks in exon regions, where the possibility of split peaks might still occur.

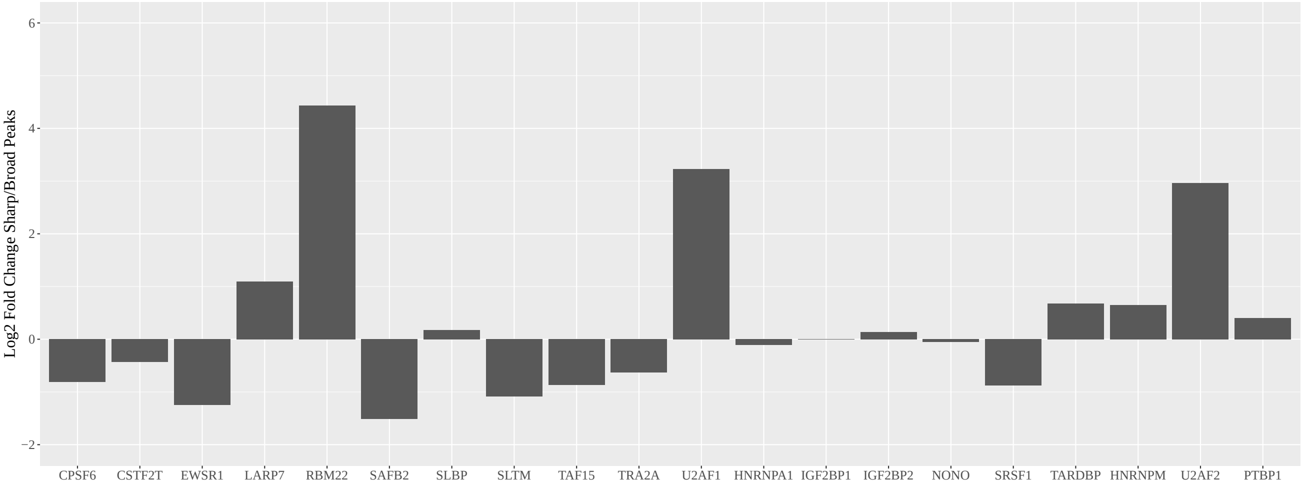

Figure 5. Log2 fold change of the number of sharp peaks versus the number of broad peaks. UniProt lists the proteins CPSF6, CSTF2T, EWSR1, LARP7, RBM22, SAFB2, SLBP, SLTM, TAF15, TRA2A, and U2AF1 with one RRM and the proteins HNRNPA1, IGF2BP1, IGF2BP2, NONO, SRSF1, TARDBP, HNRNPM, U2AF2, and PTBP1 with at least two RRMs. A clear correlation between the profile shapes and the number of RRMs was not observable. However, the proteins RBM22, SLBP, U2AF1, TARDBP, HNRNPM, U2AF2, and PTBP1 had a higher number of sharper peaks and all of them are involved in RNA splicing. Yet, proteins like SRSF1 had more broader peaks and are also involved in splicing.

As there exist no tool that can correct for split peaks, a further analysis for these proteins was required. We checked for potential split peaks by extending the peaks that fall completely into exons by five nucleotides to each side. Next, we intersected those extended peaks with introns to see if they are close to the exon boundaries. We found for U2AF1 only 27 peaks (0.72%) and for U2AF2 5 peaks (0.40%) that are potential split peaks, again deflecting the assumption of a technical artefact in the peak set of splicing factors. Thus, the sharpness of the peaks of U2AF1 and U2AF2 was potentially not the result of the peak calling.

Potential Implications

StoatyDive is a powerful tool that can evaluate and classify peak profiles. It can be used in any sequencing data analysis that involves the prediction of binding sites such as CLIP-Seq, or ChIP-Seq. Within this work, we provided an example for SLBP to show the usability of StoatyDive. First, it is possible to assess the quality of an experiment such as CLIP. Second, StoatyDive assists to evaluate the binding specificity of the protein. The normalized CV distribution produced by StoatyDive provides valuable information for the user. A protein that binds very specific will have a distribution concentrated around a normalized CV of one. A protein with a lot of unspecific bindings will have a normalized CV distribution around a value of zero. Third, StoatyDive helps to filter for specific and unspecific binding sites to investigate if the protein has multiple protein domains that have different binding mechanisms. A finer distinction can be made with the classification mode of StoatyDive. This helps to identify peak profiles with a specific shape and filter them based on the corresponding biological question and function of the protein. For example, a transcription factor might have more specific bindings (more spiky mountains), than a protein complex or a helicase (more broader mountains). Fourth, the results of StoatyDive can be used to validate a peakcaller (e.g., PureCLIP), that is to say, one can assess how many false positives are in the peak sets based on the shape. Different peakcaller might result in disparate peak sets and consequently different peak profile shapes.

StoatyDive is a very powerful, well documented, and easy to apply tool that refines the binding site detection in the data analysis such as CLIP-Seq. Nevertheless, StoatyDive is a very general tool. In the future it is worth to investigate, if StoatyDive can be used with different types of peak calling outputs and data types of sequencing data (e.g., ChIP-Seq, ATAC-Seq, Ribo-Seq, and others). It serves as a quality control and filtering step to select specific binding profiles, which therefore allows to improve other binding site prediction tools such as DeepBind [18], or any other subsequent analysis tasks, to increase the accuracy for the prediction.

Methods

Peak Correction, Extension and Coverage Calculation

StoatyDive was implemented in python (>= 3.6) and R (>= 3.4.4). The tool needs three files: the predicted binding regions of a peak calling algorithm in bed6 format, a bam or bed file that was used for the peak calling (experiment or control), and a tabular file of the chromosome size of the reference genome (Figure 6).

First, StoatyDive checks if a peak profile needs to be centered (peak correction). In the default mode, the profiles are centered by a convolution with a standard normal distribution. The maximum value of the convolution gives the nucleotide shift of the peak profile to center the peaks. So the window with the peak length is shifted to the center of the peak (Figure 6 step 1). With this approach we retain the context and take care of two problems. First, peakcallers often produce peaks that are not correctly centered. Second, dimensionality reduction methods, such as uniform manifold approximation and projection for dimension reduction (uMAP; McInnes et al. 8), are not translation invariant. Thus, two profiles with the same shape but in a different relative genomic position might end up in different locations in the new dimensional space.

After the peak correction, StoatyDive extents the peaks by default to the maximal peak length of the given peak set (Figure 6 step 2). This removes the peak length as a potential feature for the evaluation and classification. StoatyDive then calculates the read coverage (Figure 6 step 3) for each position inside a peak with the help of bedtools [7].

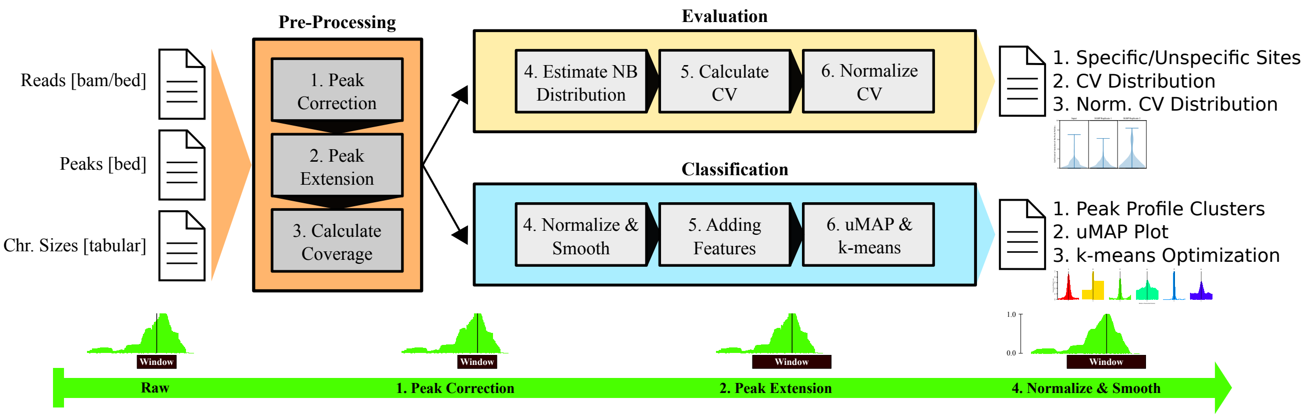

Figure 6. Overview of the StoatyDive pipeline. It consist of two major modules, namely the evaluation and the classification of peak profiles. The user has to provide reads (or events), peaks and a chromosome size file. StoatyDive then shifts the peaks to their correct center (peak correction), extents the peaks to a common length (maximal peak length of peak set or user defined value), and calculates the coverage with bedtools [7]. The peak correction can be turned off. In the evaluation, StoatyDive then estimates the read coverage as a negative binomial. From the hyperparameters it calculates the coefficient of variation (CV) and normalizes it (Equations 1 and 2). The normalized CV can then be used to divide the peaks into specific and unspecific sites. Furthermore, the CV distribution acts as a quality control between control and signal experiments. In the classification, StoatyDive first normalizes the peak profiles to remove the intensity as a feature. Then it smooths the profiles to support the data assumptions of uMAP [8] and to remove some noise. After that, it adds curve specific features to the data. The higher dimension of the data is then reduced with uMAP. StoatyDive then clusters the new data with k-means [9]. The user then obtains several plots and a table to investigate the different peak profile clusters.

Evaluation of Peak Profiles

With the results of bedtools, StoatyDive evaluates every peak i from the total set of k peaks. StoatyDive will estimate the read count for every peak as a negative binomial $X_i \sim \mathrm{NB}(r_i, p_i)$ with the hyperparameters r_i (number of hits) and p_i (probability of a hit). It then calculates the coefficient of variation (CV) for every peak. A simple estimation of the variance is not enough because the profile depends on the read coverage. Thus, to be able to compare each peak profile we have to normalize for the expected number of reads to adjust the variance. So the CV for each peak,

$$CV_i = \sqrt{\frac{1 - p_i}{r_i}}, \tag{1}$$

is calculated with the estimated hyperparameters. In the last step, StoatyDive normalizes the CV score by the max and min of all scores,

$$CV_i' = \frac{CV_i - \min(\forall CV)}{\max(\forall CV) - \min(\forall CV)}. \tag{2}$$

At the end, our defined CV score will range from $CV_i = [0, \infty]$ and the normalized score from $CV_i' = [0, 1]$, with a $CV_i' = 0$ for a more unspecific binding and $CV_i' = 1$ for a more specific one.

Classification of Peak Profiles

StoatyDive classifies the peak profiles in an unsupervised manner using uMAP [8] and k-means clustering [9]. Yet before clustering, StoatyDive processes the peak profiles. First, the profiles are normalized based on the individual maximum and minimum read count, since we are only interested in the shape of the profiles and not in the absolute read counts (Figure 6 step 4). So assuming each peak X_i has $x_1, x_2, x_j \ldots, x_n$ nucleotides, we normalized the peaks by $x_j = \frac{x_j - \min(X_i)}{\max(X_i) - \min(X_i)}$. Second, the peak profiles are smoothed (Figure 6 step 4) with a spline regression [19]. The step reduces the noise for each profile and distributes the data more uniformly on the current manifold. The latter is important since it is the data assumption of uMAP. StoatyDive further adds curve specific features to the processed peak profiles including: the number of maximal values, the area under the curve, and the arc length. StoatyDive applies uMAP to the final data with 5,000 epochs, 2 components (dim = 2), a minimum distance of 0.01 and a size of the local neighborhood of 5. The original and high dimensional profiles often suffer from the curse of dimensionality which would lead to a higher number of individual clusters. The dimensional reduction was optimized with some test data comprising four different sets of distributions: a uniform distribution, a linear distribution, an unimodal Gaussian distribution, and a bimodal Gaussian distribution. Subsequently, StoatyDive applies k-means clustering to the new data with 100 initializations, and maximal 10,000 iterations. The number of clusters k is found by convergence of the total within-cluster sum of squares and checked with the Akaike information criterion (AIC; Akaike 20). We also tested other dimensionality reduction methods (Figure 7) such as principal component analysis (PCA), a self-organizing map (SOM), and t-Distributed Stochastic Neighbor Embedding (t-SNE). However, none of them came close to the results of uMAP.

Output of StoatyDive

For the peak evaluation, StoatyDive generates a plot of the CV (Equation 1) and normalized CV (Equation 2) distribution (Figure 6). The user receives a first impression of the binding specificity of the protein of interest from the CV distribution. An unspecific binder has a CV distribution close to zero. A more specific binder has a CV distribution equal to or higher than one. The CV distribution can also be used as a quality control to compare control and signal experiments. A quality breach might have occurred if the distributions of the control and signal experiment almost look identical. A control experiment should normally have a CV distribution close to zero, with only a very few binding sites showing higher CVs.

The normalized CV distribution helps to evaluate the peaks based on the individual experiments. An empirical threshold is set at a normalized CV of 0.5 (Equation 2). Binding sites with a CV < 0.5 are more unspecific than binding sites that have a normalized CV >= 0.5. The user can change the threshold. Keep in mind, the threshold for the normalized CV is relative in accordance to the individual experiment.

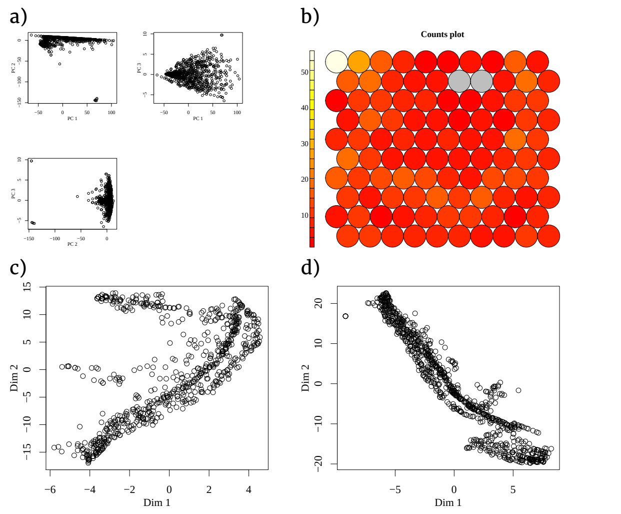

Figure 7. We tested different dimensional reduction methods such as (a) PCA, (b) SOM, and (c, d) t-SNE on the CLIP data of SLBP. The PCA has no clear clusters for replicate two, which is similar for t-SNE on replicate (c) one and (d) two. Using an optimized SOM delivers a feature layer with a very high activated hidden unit for replicate two. It is hard to see any distinct clusters from the counts (activation) of each hidden unit. uMAP can clearly separate the data into more defined clusters. Furthermore it is much easier to interpret the results of uMAP, whereas an artificial neural network, such as a SOM, generates a feature layer (hidden layer) that is hard to explain.

For the peak classification, StoatyDive generates a plot of the k-means optimization and a plot of the dimensional reduction with uMAP, which can be used to readjust the number of *k* clusters if this is necessary. The user also receives a set of example peak profiles and smoothed peak profiles of each cluster, which can be used to investigate the identified shapes. For a general trend, StoatyDive delivers average profiles for each cluster.

The final output of StoatyDive is a CV sorted table of the whole peak set, from the highest to the lowest CV. Each peak is labeled with 0, for more specific binding sites, and 1, for more unspecific sites. The table also lists for each peak the cluster number (group number) of the peak profile shape.

Important Options of StoatyDive

The peak correction (Figure 6 step 1) can be turned off. The user can also change the translocation scheme of the peak profiles to shift them based on the maximal value (summit). The maximum translocation scheme is useful for nucleotide specific events such as truncation events in the case of iCLIP data [21]. StoatyDive has also the option for a different CV score that penalizes peaks within broad plateaus. StoatyDive then adjusts the CV score of peaks that are covering a small appendage of a read stack. Furthermore, the user can provide a maximal score to StoatyDive to normalize the CV distribution (Equation 2). This option helps to compare the CV distribution between experiments in accordance to their disparate peak sizes and total amount of reads. StoatyDive also has a threshold for the normalized CV score to divide the peaks into more specific and more unspecific binding sites, which the user can change.

StoatyDive has two major parameters for the peak profile classification (Figure 6 step 6). First, the user can adjust the maximal amount of potential peak clusters identified by the k-means clustering. Yet, the final number of peak clusters will be optimized by StoatyDive. The parameter is an upper bound. However, the user has the option to force StoatyDive to use *k* specific clusters. The smoothing (Figure 6 step 4) of the peak profiles can also be adjusted by the user. The default was optimized with different test sets. Increasing the parameter (> default) might underfit the smoothing and thus lead to fewer peak clusters. A lower value (< default) might overfit and so lead to more clusters. The smoothing can also be turned off, but it is recommended to turn it on.

Availability of Supporting Source Code and Requirements

Project name: StoatyDive
Project home page: https://github.com/BackofenLab/StoatyDive
Conda: https://anaconda.org/bioconda/stoatydive
Operating system(s): Unix
biotools:StoatyDive
SciCrunch.org: SCR_018796

Availability of Supporting Data and Materials

StoatyDive provides a small dataset for a test run, which can be found in the github repository. The whole eCLIP data used in this paper, such as SLBP or RBFOX2, is listed in the supplementary of the study by Nostrand et al. [5].

Declarations

List of abbreviations

CLIP-Seq: Crosslinking immunoprecipitation in combination with high-throughput sequencing; CV: Coefficient of variation; PCA: Principal component analysis; RBP: RNA-binding proteins; RRM: RNA recognition motifs; SOM: Self-organizing map; t-SNE: t-Distributed Stochastic Neighbor Embedding.

Ethical Approval

Not applicable

Consent for Publication

Not applicable

Competing Interests

The authors declare that they have no competing interests.

Funding

This study was funded by the Deutsche Forschungsgemeinschaft (DFG, German Research Foundation) grant 322977937/GRK2344 2017 MeInBio - BioInMe Research Training Group, and Germany's Excellence Strategy (CIBSS - EXC-2189 - Project ID 390939984).

Author's Contributions

F.H. performed the computational analysis and tool developement. R.B. initialized the project, and supervised the research. F.H. and R.B. wrote the manuscript. All authors read and approved the final manuscript.

Acknowledgements

We are grateful to Gokcen Eraslan for his support.

References

1. Lee FC, Ule J. Advances in CLIP technologies for studies of protein–RNA interactions. Molecular cell 2018;69(3):354–369.
2. Jankowsky E, Harris ME. Specificity and nonspecificity in RNA–protein interactions. Nature reviews Molecular cell biology 2015;16(9):533–544.
3. Müller-McNicoll M, Neugebauer KM. How cells get the message: dynamic assembly and function of mRNA–protein complexes. Nature Reviews Genetics 2013;14(4):275.
4. Corcoran DL, Georgiev S, Mukherjee N, Gottwein E, Skalsky RL, Keene JD, et al. PARalyzer: definition of RNA binding sites from PAR-CLIP short-read sequence data. Genome biology 2011;12(8):R79.
5. Van Nostrand EL, Pratt GA, Shishkin AA, Gelboin-Burkhart C, Fang MY, Sundararaman B, et al. Robust transcriptome-wide discovery of RNA-binding protein binding sites with enhanced CLIP (eCLIP). Nature methods 2016;13(6):508.
6. Sullivan KD, Mullen TE, Marzluff WF, Wagner EJ. Knockdown of SLBP results in nuclear retention of histone mRNA. Rna 2009;15(3):459–472.
7. Quinlan AR, Hall IM. BEDTools: a flexible suite of utilities for comparing genomic features. Bioinformatics 2010;26(6):841–842.
8. McInnes L, Healy J, Melville J. Umap: Uniform manifold approximation and projection for dimension reduction. arXiv preprint arXiv:180203426 2018;.
9. Hartigan JA, Wong MA. Algorithm AS 136: A k-means clustering algorithm. Journal of the Royal Statistical Society Series C (Applied Statistics) 1979;28(1):100–108.
10. Dobin A, Davis CA, Schlesinger F, Drenkow J, Zaleski C, Jha S, et al. STAR: ultrafast universal RNA-seq aligner. Bioinformatics 2013;29(1):15–21.
11. Krakau S, Richard H, Marsico A. PureCLIP: capturing target-specific protein–RNA interaction footprints from single-nucleotide CLIP-seq data. Genome biology 2017;18(1):240.
12. Bailey TL, Boden M, Buske FA, Frith M, Grant CE, Clementi L, et al. MEME SUITE: tools for motif discovery and searching. Nucleic acids research 2009;37(suppl_2):W202–W208.
13. Lovci MT, Ghanem D, Marr H, Arnold J, Gee S, Parra M, et al. Rbfox proteins regulate alternative mRNA splicing through evolutionarily conserved RNA bridges. Nature structural & molecular biology 2013;20:1434.
14. Yeo GW, Coufal NG, Liang TY, Peng GE, Fu XD, Gage FH. An RNA code for the FOX2 splicing regulator revealed by mapping RNA-protein interactions in stem cells. Nature Structural and Molecular Biology 2009;16(2):130.
15. Singh RK, Xia Z, Bland CS, Kalsotra A, Scavuzzo MA, Curk T, et al. Rbfox2-Coordinated Alternative Splicing of Mef2d and Rock2 Controls Myoblast Fusion during Myogenesis. Molecular cell 2014;55(4):592–603.
16. Cremona MA, Sangalli LM, Vantini S, Dellino GI, Pelicci PG, Secchi P, et al. Peak shape clustering reveals biological insights. BMC bioinformatics 2015;16(1):349.
17. Parodi AC, Sangalli LM, Vantini S, Amati B, Secchi P, Morelli MJ. FunChIP: an R/Bioconductor package for functional classification of ChIP-seq shapes. Bioinformatics 2017;33(16):2570–2572.
18. Alipanahi B, Delong A, Weirauch MT, Frey BJ. Predicting the sequence specificities of DNA-and RNA-binding proteins by deep learning. Nature biotechnology 2015;33(8):831.
19. Green PJ, Silverman BW. Nonparametric regression and generalized linear models: a roughness penalty approach. Chapman and Hall/CRC; 1993.
20. Akaike H. Information theory and an extension of the maximum likelihood principle. In: Selected papers of hirotugu akaike Springer; 1998.p. 199–213.
21. Huppertz I, Attig J, D'Ambrogio A, Easton LE, Sibley CR, Sugimoto Y, et al. iCLIP: Protein–RNA interactions at nucleotide resolution. Methods 2014;65(3):274–287.

Manuscript tex

Click here to access/download
Supplementary Material
main.tex

bib

Click here to access/download
Supplementary Material
paper-refs.bib

style

Click here to access/download
Supplementary Material
oup-contemporary.cls
