# Supplementary material for: StoatyDive: Evaluation and classification of peak profiles for sequencing data
Source: Gigascience. 2021 Jun 18;10(6):giab045. doi: 10.1093/gigascience/giab045 (PMC8212874; doi:10.1093/gigascience/giab045)
Supplement: giab045_GIGA-D-20-00218_Revision_1 [file giab045_giga-d-20-00218_revision_1.pdf]

GigaScience

StoatyDive: Evaluation and Classification of Peak Profiles for Sequencing Data

--Manuscript Draft--

**Manuscript Number:**	GIGA-D-20-00218R1
**Full Title:**	StoatyDive: Evaluation and Classification of Peak Profiles for Sequencing Data
**Article Type:**	Technical Note
**Funding Information:**	Deutsche Forschungsgemeinschaft (322977937/GRK2344 MeInBio) — Mr. Florian Heyl<br>Deutsche Forschungsgemeinschaft (390939984 EXC-2189) — Prof. Dr. Rolf Backofen

Abstract:

Background

The prediction of binding sites (peak calling) is a common task in the data analysis of methods such as crosslinking immunoprecipitation in combination with high-throughput sequencing (CLIP-Seq). The predicted binding sites are often further analyzed to predict sequence motifs or structure patterns. When looking at a typical result of such a high-throughput experiments, the obtained peak profiles differ largely on a genomic level. Thus, a tool is missing that evaluates and classifies the predicted peaks based on their shapes. We hereby present StoatyDive, a tool that can be used to filter for specific peak profile shapes of sequencing data such as CLIP.

Findings

With StoatyDive we are able to classify peak profile shapes from CLIP-seq data of the histone stem-loop-binding protein (SLBP). We compare the results to existing tools and show that StoatyDive finds more distinct peak shape clusters for CLIP data. Furthermore, we present StoatyDive's capabilities as a quality control tool and as a filter to pick different shapes based on biological or technical questions for other CLIP data from different RNA binding proteins with different biological functions and number of RNA recognition motifs. We finally show that proteins involved in splicing, such as RBM22 and U2AF1, have potentially more sharper shaped peaks than other RNA binding proteins.

Conclusion

StoatyDive finally fills the demand for a peak shape clustering tool for CLIP-Seq data that fine tunes downstream analysis steps such as structure or sequence motif predictions and that acts as a quality control.

**Corresponding Author:**	Florian Heyl<br><br>GERMANY
**Corresponding Author Secondary Information:**	
**Corresponding Author's Institution:**	
**Corresponding Author's Secondary Institution:**	
**First Author:**	Florian Heyl
**First Author Secondary Information:**	
**Order of Authors:**	Florian Heyl<br>Rolf Backofen
**Order of Authors Secondary Information:**	

**Response to Reviewers:**	\section{Reviewer 1}<br><br>Major comments:

Powered by Editorial Manager® and ProduXion Manager® from Aries Systems Corporation

1)I find that there's an imbalance where the most important findings are stated very briefly (sometimes without figures), whereas significant text and figure space is devoted to more descriptive analyses that lack a clear conclusion.

For example, to me the most important finding from the method is that the use of different peak shapes enables separation of real versus artifact peaks, which is aided by the authors use of SLBP (which has a well-characterized specific role in binding a specific hairpin structure in histone 3' UTRs), However, this result is stated in Table 1 without statistical analysis (enrichment, significance, etc) and a couple sentences of text. The manuscript would be stronger with additional analysis and description here (what is the sensitivity/specificity tradeoff in using the shape group / CV cutoffs described here? How do those change with different cutoffs?)

\begin{answer}
Thank you very much for your suggestion as it contributed a lot to the paper.

PureCLIP already filters for significant peaks and does not give any enrichments (no fold changes or p-values). Thus all peaks of SLBP that are mentioned in table 1 are significant peaks. So it was not possible to define sensitivity/specificity or other features for table 1.

We made the effort and applied the peak shape clustering on the CLIPper peaks for replicate 2 (all peaks). Therefore, we add the new Supplements 3 in the paper and a whole new section called "Optimizing StoatyDive with the data of SLBP", where we go deeper about StoatyDive based on different clusters, CV cutoffs and peak sizes.

As a short summary, we show that the best CV cutoff is at 0.2 and that the cluster has to be chosen carefully in order to reduce some noise. We also show, that we can remove some noise from the initial CLIPper peak set. Furthermore, we demonstrate that the best peak length for StoatyDive is around 70 nucleotides.

The section includes more findings, and we would invite you to read the new section.

In addition we included also more statistical analysis to clarify our findings. So we state for the different CV distributions between the replicates and the control of the SLBP data: "Although the CV distributions of the input control and replicate 1 of the SLBP data differed significantly (one-sided Wilcoxon test p-value = \$0.03\$), both contained a lot of regions with a CV close to zero (Figure 2, both with a mean CV of 0.47). In contrast, the CV distribution of replicate 2 was distinct (p-value \$< 0.05\$ to input control and replicate 1) since it had more peaks with a higher CV (mean CV of \$1.41\$) and thus more specific binding events (e.g., Figure 1a, \$CV \approx 5.3\$)."

We also calculated the ACC for the tool comparison: "StoatyDive classified most peaks correctly into the three peak shape groups (Figure 5a, ACC = 0.87)." and later "SIC-ChIP identified up to six different peak shapes (Figure 5b), whereas FunChIP found three (Figure 5c and d, ACC = 0.6)."
\end{answer}

In contrast, two paragraphs are devoted to a fairly superficial motif analysis - it's not clear from the text which motifs the authors believe are real and which are false-positives, which makes it descriptive but unclear what the conclusion is (other than that they are 'different'). Although this does not appear to be discussed in the text, it seems that the GCUCUUU motif matches the known SLBP hairpin (e.g. Fig 2 from Tan et al, PMID 23329046), which would again suggest that the sharp and plateau classes are real whereas the broad class motifs are artifacts; this should be more clear.

\begin{answer}
So far we do not believe any of the motifs are true or false. We initially started to analyse SLBP and tried to show that different sequence motifs can be found with different peak shapes. SLBP is still a good candidate to show, that different peak shapes are either artifacts or real binding sites. Yet, it is important that the user checks the biological correctness of those clusters also for other proteins. We made a statement in the paper to clarify this issue by noting:

"Yet, we cannot confirm the biological truth behind those motifs as it requires further experiments to verify them."
\end{answer}

2)The authors use the ENCODE data, but then re-perform peak calling with PureCLIP to perform their analyses. This isn't necessarily a problem; however, in the context of the major point of the manuscript being discussion of peak shapes, I find it perplexing that this is done with no further discussion, as one of the main differences between PureCLIP and the CLIPper algorithm (which was used in the peak calls provided by ENCODE) is that CLIPper explicitly uses peak shape as a feature to perform spline-fitting as part of the cluster identification process (Lovci et al. PMID 24213538). I can thus see an argument for using a peak caller that does not incorporate this feature in order to enable downstream analyses comparing peak shape; but I do think there should be some ultimate discussion of whether the classification and clustering described here provides additional power over the spline-fitting used in the initial CLIPper calls.

\begin{answer}
We showed and analyzed also the CLIPper peaks (peaks which are significant and not significant) of SLBP in the paper. We demonstrated that also CLIPper's results could benefit from StoatyDive. Furthermore, the intention behind StoatyDive is an analysis independent of the peak caller. Of course it can be used to remove potential artifacts of the data processing and peak calling, but it is also worth to investigate a set of significant peaks with StoatyDive. You can cluster those peaks in different groups, which might reflect different biological properties. That to say, peak shape clustering comes after the peak calling and is a refinement or selection step for a deeper analysis of the peak set.
\end{answer}

Specific comments:

The authors conclude a hypothesis that "peak profiles shaped like plateaus were mainly PCR duplicates that were less informative." - this is a conclusion that is not supported by data, but is both confusing (the eCLIP datasets contain unique molecular identifiers and thus shouldn't contain significant PCR duplicates) and also is easy to test from the data used (those peaks should have higher mutation rates or PCR duplication rates among pre-PCR-duplicate-removed reads than other peaks).

\begin{answer}
We removed this statement as we saw that those peaks are indeed not PCR duplicates and basically a distinctive shape of the data.
\end{answer}

I'm confused by the sentence "We looked further and found sharper peaks located on stem loops targeted by SLBP such as RNU7-1 RNA ..." - do the authors believe these are real or artifact peaks? Either way there should be some discussion of why (and why they are specifically called out here)

\begin{answer}
RNU7-1 is a potential target of SLBP. We have removed the other stem loop protein from the paper because we could not find any literature to verify it as a target of SLBP. The other stem loop was an assumption based on our own past findings.

The paper now states: "For example, we found a sharper peak located on RNU7-1 RNA (U7 small nuclear 1) that contains a stem loop that might be potentially targeted by SLBP [citation]. The peak got a CV of 3.9 and was classified into the peak profile cluster 3 of replicate 2."
\end{answer}

Many figures need editing to be more visually clear. In addition to fonts (Fig. 3 and 4 in particular have multiple panels that illegibly small at standard resolution), the figures would be significantly clearer if additional labels are provided to enhance flow through the analyses in the paper. For example - Table 1 refers to separation of the 7 classes in Fig. 3c/d into 4 shape 'groups'; it would be helpful to have those labeled in Fig 3 as

well.

\begin{answer}
We included your recommendations and changed the font as well as layouts for all figures. Therefore we invite you to check the new figures. We hope we could improve the readability and intuitive understanding.
\end{answer}

I think it needs to be clearer exactly what the analysis being done in the last section (of other RBPs) - after multiple reads I'm still a bit confused how the earlier analysis (with unsupervised clustering and manual annotation of classes) connects with the last analysis (with a hard cutoff of 'sharp' vs 'broad' peaks). The text in this section (Fig 7) refers to 'broader' versus 'sharper' shaped peaks; however I'm not clear on what defines those terms (the methods text suggests that for standard analysis the authors set a CV cutoff of 0.5 to define 'specific' versus 'unspecific'; I'm assuming but not clear that it's that and not manual annotation of unsupervised clustering classes as was done for SLBP?)

\begin{answer}
It is true, we have not done a manual classification into \textbf{broad} and \textbf{sharp} as we have done for the data of SLBP. SLBP was a detailed example and finer analysis, whereas the last part was done more automatically. We stayed very conservative and looked at the CV distribution of each protein to find a good cutoff for broad vs sharp. It is to note that the CV distribution depends on the size of the peaks and the protein being analyzed. The initial cutoff of 0.5, was done on initial observations. Now, we know that a very conservative cutoff is at 0.2. We state in the paper in section "Investigation of eCLIP Protein Profiles":

"All peaks were therefore extended or shrunk to a length of 77 nucleotides. This was based on the observation that the third quartile of all peaks from all proteins was 77 nucleotides long (see Supplementary Figure 4). In addition, StoatyDive achieved for the SLBP data a better TPR and MCC with a peak size of 70 and a CV threshold of 0.2 (see Supplementary Figure 3). The results of SLBP showed that it may be wise to combine the clustering and the CV threshold to assess the profile landscape of other proteins. We therefore defined a peak as sharp if it had a CV > 0.2 and it fell into a cluster that was generally sharper. A cluster was declared as sharp if the median CV of the cluster was bigger than the median CV of the whole peak set. All other peaks were classified as broad."
\end{answer}

\section{Reviewer 2}

1. comment: I am surprised that the authors focused their analyses on the SLBP protein and not on the more well studied RBP.

\begin{answer}
It is true that there is no information about a sequence or structure motif for SLBP, but it is well known (see reviewer 1), that SLBP binds distinctively histone mRNAs at the 3' UTR region. Thus, it was interesting to see if we can find differences in peak shapes and show that we can differentiate between specific and unspecific binding with StoatyDive. Furthermore, it was interesting to see if we can find other distinct binding shapes for SLBP that might reflect different biological mechanics since the protein has two functions in eukaryotic cells, serving as transport and translation factor. SLBP is one of the proteins that can be used to optimize CLIP-Seq data analysis and is therefore a good candidate for StoatyDive.
\end{answer}

However, the authors did find interesting observations for the SLBP protein by analysing eCLIP samples in K562 cell lines, including low reproducibility between the replicates, which haven't been fully investigated in this manuscript. I think it would be important to address these observation in a more systematic manner:
*Have you checked how much the Coefficient of Variation (CV) changes between other

replicates in other eCLIP samples, including eCLIP HepG2 samples? It would also be important to include CV results for all other samples that you analysed in this study (RBM22, U2AF2, PTBP1…). Do other eCLIP samples also show similar differences between the replicates or is the SLBP protein one of the exceptions?

\begin{answer}
We included the CV distributions of the other proteins for the K562 cell line in the supplementary of the paper (Supplementary Figure 1) and made a short statement in the section "Peak Profile Landscape Reveals Low Reproducibility of Binding Sites", where we state: "Checking the CV distributions of other CLIP-Seq datasets such as TAF15, TARDBP, and HNRNPA1, which we will analyze further in a later section, (Supplementary Figure 1), we saw that the CV distributions also had differences between the replicates (two-sided Wilcoxon test p-value < 0.05). However, this does not mean a low quality of the data and just highlights that it is important to do replicates in order to quantify biological and technical variance as noted in a previous CLIP study [citation]."

We think that this is already enough for a demonstration that the CV might vary between replicates. An even deeper analysis including the HepG2 cell line is beyond the scope of the paper.
\end{answer}

Is this variation between replicates more specific for the eCLIP protocol or is it also common in other CLIP-seq methods such as iCLIP, irCLIP or PAR-CLIP?

\begin{answer}
A variation between replicates can of course happen (see AC Jungkamp et al. (2011), In vivo and transcriptome-wide identification of RNA binding protein target sites, and X. Chen et al. (2015), Statistical issues in binding site identification through CLIP-seq). This can come from different sources: execution of the experiment, changes in lab staff, technical variances, biological variances and more. It is hard to figure out what the actual reason was, especially since we used data that we did not generated in our group. It is beyond the scope of this paper to investigate other CLIP protocols, since we wanted to check the power of StoatyDive. However, we would analyze this in the future, when we will update StoatyDive and give it even more power.
\end{answer}

*How many peaks identified in replicate one overlap with replicate two?

\begin{answer}
Replicate one had 659 peaks and replicate two 935. The overlap is the mentioned 899 peaks. We made a robust peak detection (intersecting and merging the two peak sets) to define a robust set of peaks. So we use these 899 peaks to evaluate both replicate one and replicate two. Replicate two has perhaps more peaks, but some of those peaks can be merge into a bigger peak region that is covered by one peak of replicate one.

From those peaks, we have 236 exonic and 151 intronic peaks for replicate one, and 374 exonic and 289 intronic peaks for replicate two. So replicate two has for both 136 exonic and 136 intronic peaks more. So there is a difference in the number of peaks between replicate one and two but the difference between exonic and intronic is the same.
\end{answer}

*Is the library size similar between the replicates and have you done any more detailed quality controls between the replicates (number of uniquely mapped reads, sequencing errors etc.)? Maybe a poor quality or low coverage could explain the difference.

\begin{answer}
We checked the raw data quality and there are no quality breeches, except the high duplication level and adapters you usual see in CLIP-data.

Furthermore, the supplementary data table by Van Nostrand et al. (2016, Robust transcriptome-wide discovery of RNA-binding protein binding sites with enhanced CLIP

(eCLIP)) state for the aligned files:

\textbf{Replicate 1}\\
\# Input read: 13,553,232\\
\# Uniquely mapped read: 3,558,228\\
\# Usable read: 2,664,051\\
\% usable (out of uniquely mapped): 0.748701601\\ \\

\textbf{Replicate 2}\\
\# Input read: 15,493,437 \\
\# Uniquely mapped read: 3,354,237 \\
\# Usable read: 2,520,409 \\
\% usable (out of uniquely mapped): 0.751410529

So the library sizes are almost the same.
\end{answer}

*Would it be possible that one replicate has more cytoplasmic binding sites than the other? One way of testing this would be to check how many peaks that you identify are in intronic regions vs exonic regions, for each replicate separately. Also, how does the CVs and shape profile of the peaks change between exonic and intronic peaks? Maybe this sample has a higher binding specificity in the nucleus than in cytoplasm or vice versa.

\begin{answer}
Thank you very much for this interesting question and idea.

Indeed there was a big difference for exonic and intronic peaks. We dedicated a new chapter in the paper, where we state: "To investigate further differences between the two replicates, we split the peak set into peaks overlapping with exons and introns (see Figure~3). SLBP is a translation and transport factor, which is present in the cytoplasm as well as nucleus [citations]. Therefore, the replicates could have different binding events, where one replicate might have more events in cytoplasm and the other more in the nucleus. For replicate 1 we got 236 exonic and 151 intronic peaks for replicate 1, and 374 exonic and 289 intronic peaks for replicate 2. So replicate 2 had 136 exonic and 136 intronic peaks more. Notably, we can see a huge CV difference when comparing the intronic peaks of the two replicates, with a mean CV of 0.23 for replicate 1 and 1.26 for replicate 2 (one-sided Wilcoxon test P-value < 0.05). The exonic peaks on the other hand were more similar (mean CV = 0.48 and 0.90, respectively), but still the CV distributions were significantly different (one-sided Wilcoxon test P-value < 0.05). It is not clear why this difference appeared, but our observations stress out how important it is to perform an experiment with replicates."
\end{answer}

2. comment: "It is important to note that peaks shaped like plateaus might be false positives. These peaks most likely corresponded to PCR duplicates that were not real binding sites. We have removed PCR duplicates during the pre-processing of the read library, but some duplicates might still be in the data. Sequencing errors in the unique molecular identifiers (UMI) are a common reason."
*Are there any changes in shape profiles between intronic and exonic peaks? To filter out false positive peaks, you could use ENCODE RNA-seq data for the same cell line and analyse peaks and their shapes in expressed transcripts. How many False positives are present in non-expressed transcripts and do they show different motifs and different shapes of their peaks?

\begin{answer}
We removed this statement in the paper as we observed that those peaks are not PCR duplicates and a distinct peak shape.

While your suggestion still pose an interesting question for future research, it goes beyond the scope of this paper and we would do this part with a newer version of StoatyDive. We want to expand StoatyDive with a comparison mode to allow two or more samples to be compared (cluster them together) in their profile shape landscape

Powered by Editorial Manager® and ProduXion Manager® from Aries Systems Corporation

(e.g., control vs CLIP). This would give a better distinction of false positives.
\end{answer}

3. comment: "We investigated also the sequence motifs of sharp and broad peak profiles of the protein RBFOX2 (eCLIP data from the study by Van Nostrand et al. [5]), because it has the conserved sequence motif TGCATG, which is enriched in the RBPFOX2's binding sites [13, 14, 15]. We have found the conserved motif only in sharp peaks (Table 3), but the broader profiles also had an enriched motif. It is worth investigating if RBFOX2 has some unspecific binding preferences with that specific motif. The results for RBFOX2 again demonstrated that different peak shape groups result in different sequence motifs."
*A recent paper by Bridget E. Begg et al. identified secondary binding motifs from their RBFOX2 CLIP experiments (Begg et al. 2020). These motifs are specific for intronic and 3'UTR regions (Bridget E. Begg et al., Figure 3). Could the authors also identify these motifs by separating their peak shape profiles into intronic and 3'UTR region?

\begin{answer}
Thank you very much for this questions, however we have removed this chapter from the paper as it was too short and disrupted the flow of the paper.

We did a short analysis for your question, but there where no interesting results or significant differences of the shapes between exonic, intronic and 3'UTR regions.
\end{answer}

4. comment: "All peaks were therefore extended or shrank to a length of 77 nucleotides. This was based on the observation that the third quartile of all peaks (from all proteins) was 77 nucleotides long."
*The 77 nucleotides window size for all RBPs could be too broad to identify sharp peaks for proteins with specific bindings. Anob M. Chakrabarti et al. has shown in their review (Chakrabarti et al. 2018), that the window size parameters can modify the sensitivity and specificity of the data and it needs to be adapted for each RBP. For example, PTBP1 can have multiple binding sites just next to each other, where the broad window size would detect all the protein binding sites as one large binding site instead of multiple sharp ones. Could the authors demonstrate and comment on at least one example of how the window size can affects peak finding specificity and their shape profiles?

\begin{answer}
It is true that different window sizes affect the results. We did an additional analysis to show the effect of the peak length. We dedicated now a complete new chapter "Optimizing StoatyDive with the data of SLBP", where we go deeper about StoatyDive based on different clusters, CV cutoffs and peak sizes.

As a short summary, we show that the best CV cutoff is at 0.2 and that the cluster has to be chosen carefully in order to reduce some noise. We also show, that we can remove some noise from the initial CLIPper peak set. Furthermore, we demonstrate that the best peak length for StoatyDive is around 70 nucleotides.

The section includes more findings, and we would invite you to read the new section.

A size of 77 nucleotides might be long, yet it is good to discriminate the profiles that you have mentioned. We state later in the paper now:
"All peaks were therefore extended or shrunk to a length of 77 nucleotides. This was based on the observation that the third quartile of all peaks from all proteins was 77 nucleotides long (see Supplementary Figure 4). In addition, StoatyDive achieved for the SLBP data a better TPR and MCC with a peak size of 70 and a CV threshold of 0.2 (see Supplementary Figure 3). The results of SLBP showed that it may be wise to combine the clustering and the CV threshold to assess the profile landscape of other proteins. We therefore defined a peak as sharp if it had a CV > 0.2 and it fell into a cluster that was generally sharper. A cluster was declared as sharp if the median CV of the cluster was bigger than the median CV of the whole peak set. All other peaks were classified as broad."
\end{answer}

Powered by Editorial Manager® and ProduXion Manager® from Aries Systems Corporation

5. comment: "So we investigated the number of peaks that fall into introns (90\% overlap) for the proteins that are involved in the splicing process. The proteins PTBP1 (85\%), RBM22 (62\%), TARDBP (79\%), and HNRNPM (87\%) had more than 50\% of peaks in introns, which detected the assumption of split peaks. However, the proteins U2AF1 (17\%), and U2AF2 (15\%) had more peaks in exon regions, where the possibility of split peaks might still occur. We found for U2AF1 only 27 peaks (0.72\%) and for U2AF2 5 peaks (0.40\%) that are potential split peaks, again defecting the assumption of a technical artefact in the peak set of splicing factors. Thus, the sharpness of the peaks of U2AF1 and U2AF2 was potentially not the result of the peak calling."
* The authors examined RBPs that are involved in the splicing process, including U2AF1 and UAF2, which recognises 3' splice sites and recruits the spliceosome. The authors claim that the majority of the identified peaks from both proteins fall into the exonic region. This statement contrasts with previous studies including my preliminary analysis (see Figure ??? below). The figure shows a normalised coverage of cDNA-starts for the same eCLIP U2AF1 and U2AF2 K562 and HepG2 samples relative to the 3' splice site region, showing a dominant enrichment of the intronic region for both proteins which also agrees with the previous CLIP studies (Zarnack et al. 2013; Sutandy et al. 2018; Briese et al. 2019). The authors should examine their peak analyses in these results further to identify the reason for such a difference.

\begin{answer}
Thank you for your suggestion. It was not our intention to make this claim. We just wanted to investigate a potential shape bias, because of a peak phenomenon that we called "split peaks". We state now in the paper: "We also checked whether our result that RBPs involved in
splicing have sharper peaks can have technical reasons. In this case, a splicing related protein could have more peaks that are split over two exons, which are detected by the peakcaller as two separate but sharp peaks (split peak). So we investigated the number of peaks that fall into introns (90% overlap) for the proteins that are involved in the splicing process. We used bedtools set to a strict overlap (intersect -u -s -f 0.9) to investigate potential split peaks." and later "It is important to note that this does not mean that the aforementioned proteins bind generally more introns or exons."

We checked and assume that our constraint for bedtools with 90% overlap is the reason for this different observation to your findings. When we decrease the overlap, more peaks overlap with introns.
\end{answer}

\section{Reviewer 3 -- finished}

*Figure 2 indicates that replicate 1 of SLBP CLIP-seq is indistinguishable from the negative control from the perspective of the coefficient of variation. However, this replicate is then used throughout the analyses of Figure 3 and Tables 1 and 2. If Replicate 1 did work experimentally, it would contradict the authors' proposal that evaluating the coefficient of variations across peak-profiles (Figure 2) is a valuable method of examining CLIP-seq quality control.

\begin{answer}
Thank you for your question. We state in the paper: "Although the CV distributions of the input control and replicate 1 of the SLBP data differed significantly (one-sided Wilcoxon test p-value = 0.03), both contained a lot of regions with a CV close to zero (Figure 2, both with a mean CV of 0.47). In contrast, the CV distribution of replicate 2 was distinct (p-value < 0.05 to input control and replicate 1) since it had more peaks with a higher CV (mean CV of 1.41) and thus more specific binding events (e.g., Figure 1a, CV ≈ 5.3). Yet, some potential binding sites were more unspecific with a CV closer to zero (e.g., Figure 1b, CV ≈ 0.006)."

Even though the distribution between the input control and replicate one looks quite similar the p-value is < 0.05, thus replicate one is significantly different in the CV distribution in comparison to the input control.

Powered by Editorial Manager® and ProduXion Manager® from Aries Systems Corporation

	Furthermore, the CV distribution is just one quality factor for a CLIP experiment. We state in the paper: "The CV is just one quality factor and we recommend to test other features as well, such as the read coverage correlation."<br>\end{answer}<br><br>*Figure 3 shows the distinct patterns present across two replicates of SLBP CLIP-seq. It is unclear how unique these patterns are to SLBP CLIP-seq or if they are an artifact of CLIP-seq assay. It would be important to show the average tag distribution of the control experiment at sample peaks (separated by distinct peak profile) in order to demonstrate that the discovered peak profiles exist only within sample-targeted CLIP-seq.<br><br>\begin{answer}<br>We included the results of the input control in the paper and state: "We also checked the uniqueness of the shapes by analyzing the peaks based on the reads from the size-matched input control (see Supplementary Figure 2). StoatyDive had just identified four different clusters, encompassing mountain shaped peaks, as well as plateaus, and constant peaks. It was to be expected to find similar shapes in the control, because the biggest challenge for peak calling is the identification of enriched sites with different shapes between control and CLIP data [12, 13]. In a future version of StoatyDive, we will include a mode to check peak shapes between samples to see if we could improve peakcalling results with a peak shape comparison."<br>\end{answer}<br><br>**The underlying RNA sequence of the peaks should also be investigated with regards to whether mappability (or a specific A/U/G/C content) is responsible for producing broad vs narrow peaks. Are broad peaks a function of less mappable reads producing unnatural peaks? It would important to demonstrate that StoatyDive is not just modeling the underlying sequence mappability at the region.<br><br>\begin{answer}<br>We can immediately excluded this because we have used only significant peaks from CLIPper and PureCLIP. PureCLIP searches for significant peaks (combining both CLIP replicates) with an HMM model. CLIPper from the CLIPper pipeline is run on each replicate separately and both peaks sets are then tested for robustness with an irreducible discovery rate (IDR) analysis. That is to say, mappability and low quality of the peaks can be excluded since (a) only significant and robust peaks are used from the peakcalling, and (b) only high-quality (quality score $>$ 20) and unique mappable reads were used. So the comparison of broad and narrow peaks really is just done on the CLIP-Seq enrichment.<br>\end{answer}<br><br><br>\section{Final remark:}<br><br>We thank all reviewers for their comments, critics, ideas and questions. It was very helpful and improved the paper a lot. You will find changes in the paper marked in red. Furthermore we added a lot of new supplements. Please take a look. Thank you very much for your time and effort.
**Additional Information:**	
**Question**	**Response**
Are you submitting this manuscript to a special series or article collection?	No
**Experimental design and statistics**<br><br><br>Full details of the experimental design and statistical methods used should be given in the Methods section, as detailed in our Minimum Standards Reporting Checklist.	Yes

Powered by Editorial Manager® and ProduXion Manager® from Aries Systems Corporation

Information essential to interpreting the data presented should be made available in the figure legends.<br><br>Have you included all the information requested in your manuscript?	
**Resources**<br><br>A description of all resources used, including antibodies, cell lines, animals and software tools, with enough information to allow them to be uniquely identified, should be included in the Methods section. Authors are strongly encouraged to cite Research Resource Identifiers (RRIDs) for antibodies, model organisms and tools, where possible.<br><br>Have you included the information requested as detailed in our Minimum Standards Reporting Checklist?	Yes
**Availability of data and materials**<br><br>All datasets and code on which the conclusions of the paper rely must be either included in your submission or deposited in publicly available repositories (where available and ethically appropriate), referencing such data using a unique identifier in the references and in the “Availability of Data and Materials” section of your manuscript.<br><br>Have you have met the above requirement as detailed in our Minimum Standards Reporting Checklist?	Yes

Manuscript
Click here to access/download;Manuscript;main.pdf

OXFORD (GIGA)n SCIENCE

GigaScience, 2017, 1–10

doi: xx.xxxx/xxxx
Manuscript in Preparation
Technical Note

TECHNICAL NOTE

StoatyDive: Evaluation and Classification of Peak Profiles for Sequencing Data

Florian Heyl[1,*] and Rolf Backofen[1,2,*]

[1]Bioinformatics Group, Department of Computer Science, University of Freiburg, Georges-Köhler-Allee 106, 79110 Freiburg, Germany and [2]Signalling Research Centres BIOSS and CIBSS, University of Freiburg, Schaenzlestr. 18, 79104 Freiburg, Germany

*heylf@informatik.uni-freiburg.de, backofen@informatik.uni-freiburg.de

Abstract

Background The prediction of binding sites (peak-calling) is a common task in the data analysis of methods such as crosslinking immunoprecipitation in combination with high-throughput sequencing (CLIP-Seq). The predicted binding sites are often further analyzed to predict sequence motifs or structure patterns. When looking at a typical result of such a high-throughput experiments, the obtained peak profiles differ largely on a genomic level. Thus, a tool is missing that evaluates and classifies the predicted peaks based on their shapes. We hereby present StoatyDive, a tool that can be used to filter for specific peak profile shapes of sequencing data such as CLIP.
Findings With StoatyDive we are able to classify peak profile shapes from CLIP-seq data of the histone stem-loop-binding protein (SLBP). We compare the results to existing tools and show that StoatyDive finds more distinct peak shape clusters for CLIP data. Furthermore, we present StoatyDive's capabilities as a quality control tool and as a filter to pick different shapes based on biological or technical questions for other CLIP data from different RNA binding proteins with different biological functions and number of RNA recognition motifs. We finally show that proteins involved in splicing, such as RBM22 and U2AF1, have potentially more sharper shaped peaks than other RNA binding proteins.
Conclusion StoatyDive finally fills the demand for a peak shape clustering tool for CLIP-Seq data that fine tunes downstream analysis steps such as structure or sequence motif predictions and that acts as a quality control.

Key words: CLIP-Seq; data analysis; peak shape clustering; RNA; protein

Findings

Background

The biological function of a protein is determined by its interaction partners and the mode of interaction. Studying these interactions broadens our horizon about the cellular mechanisms such as alternative splicing and post-transcriptional regulation. Crosslinking immunoprecipitation in combination with high-throughput sequencing (CLIP-Seq) fathoms these interactions. CLIP-Seq investigates all interactions between an RNA binding protein (RBP) and its target RNAs [1]. CLIP-Seq thus scrutinizes the post-transcriptional regulation by RBPs. Prediction of binding regions (peak-calling) is a crucial step in the data analysis of methods such as CLIP-Seq. Before the peak analysis there is typically no evaluation and classification of the peak characteristics. Yet, the obtained peak set might have different peak profiles that are worth to filter to refine a downstream analysis. The different peak shapes are the result of several biological and technical problems.

Many RBPs have several binding domains with different binding affinities, and are often part of protein complexes, leading to an intricate binding pattern. As described in a review by Jankowsky, Eckhard and Harris, Michael E [2], there are specific and unspecific binders. Examples for unspecific binders are often RBPs that need to bind many RNAs such as mRNA export factors [3]. Another example of common unspecific binders are RNA helicases. However, even more specific

Compiled on: November 26, 2020.
Draft manuscript prepared by the author.

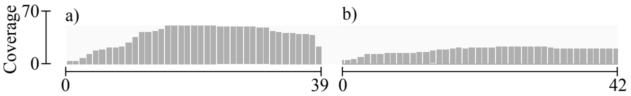

Figure 1. We show 2 significant peaks of a CLIP experiment for the protein SLBP (ENCSR483NOP, replicate 2). One can see peaks with drastically different peak profiles, pointing towards more specific (a) or unspecific (b) binding. Current analysis of CLIP binding sites is typically based on manual inspection of a few peaks. Thus a general tool is missing that allows to filter, cluster and quantify peak profiles and therefore refine downstream analysis tasks for data such as CLIP. StoatyDive assists to find and distinguish peaks like (a) and (b).

RBPs bind RNAs in large range of affinities, indicating that different binding sites vary in their binding specificity. While many factors, such as the affinity of an RBP for the binding site and the concentration of the protein and RNA, influence the binding specificity, it is likely that these factors are manifested in the CLIP binding profile landscape. At this point, however, no tool exists that can be used to study this possibility in more detail.

In addition, technical biases might change the peak profile landscape. Binding artifacts might be introduced during read library preparation. Protocol biases, for example, PAR-CLIP biases that are introduced by endonuclease and photoactivatable nucleosides [4], might also affect the binding site predictions. On top, the peak caller itself might generate specific peak profiles and false positives, which the user might not want to have in their data.

This leads to many questions in the data analysis of binding sites that can currently not be answered adequately. Examples are: Does my protein of interest bind generally more specific (Figure 1a) or more unspecific (Figure 1b)? Does my RBP of interest have more than one binding motif? Does my experiment have any quality issues, meaning, do my reads come from unspecific bindings because of library preparation artifacts? Does my protocol generate biases? Do I have false positives in the set of predicted peaks from my peak caller of choice?

We hereby present StoatyDive, a tool to evaluate and classify peak profiles to help to answer the aforementioned questions. StoatyDive uses the whole peak profiles as well as predefined features to do a peak shape clustering for sequencing data. In this paper, we will test StoatyDive on CLIP data of the eCLIP protocol from the histone stem-loop-binding protein (SLBP) from the study by Van Nostrand et al. [5]. SLBP has been reported to be a histone mRNA export and translation factor [6]. StoatyDive delivers several plots and a table to assess the different binding profiles of a protein. The tool assists to select specific and unspecific binding sites and to find similar shaped peak profiles. Thus, we try to refine the obtained peaks of the SLBP data to find more specific sites of SLBP. It also helps as a quality assessment to validate a CLIP-Seq or any other binding experiment. Later in the paper, we use StoatyDive to investigate the peak profile landscape of different RBPs with different biological functions and different number of RNA recognition motifs (RRM). StoatyDive comes with some test data and a quick installation guide.

Data Preparation of SLBP and Analysis

We used eCLIP data of the histone stem-loop-binding protein (SLBP; ENCSR483NOP; GSE91802; Van Nostrand et al. 5). The data comprised 2 CLIP replicates and a size-matched input control from immortalised myelogenous leukemia cells (K562). We processed the data with the snakemake pipeline SalamiSnake (https://github.com/BackofenLab/SalamiSnake, v0.0.1) for eCLIP data. SLBP has been reported to be cytoplasmic, but to be present also in the nucleus [6]. Thus, we mapped the reads against the human genome (version hg38) with STAR [7], but also taking the transcriptome into account. We predicted potential binding sites of SLBP with PureCLIP [8], which we ran for each CLIP replicate separately, taking the input control into account. We extended the predicted binding regions by 20 nucleotides left and right because PureCLIP often underestimates the binding region. We further fused the predicted peaks from each CLIP replicate with bedtools [9] to get a robust set of predicted binding sites, which resulted in 899 robust peaks. We executed StoatyDive (v1.1.0 with umap v0.2.5.0) with length normalization, a penalty for broader plateaus, and peak profile smoothing. The complete call was: *StoatyDive.py -a peaks.bed -b reads.bam -c hg38.chrom.sizes.txt --peak_correction --scale_max 10 --border_penalty --sm*.

Peak Profile Landscape Reveals Low Reproducibility of Binding Sites

The user obtains from StoatyDive a distribution of the coefficient of variation (CV), calculated for each peak, to get a broad overview of the peak profile landscape of their experiment (see Methods). Broader peaks tend to have a CV ≈ 0. Although the CV distributions of the input control and replicate 1 of the SLBP data differed significantly (one-sided Wilcoxon test P-value = 0.03), both contained a lot of regions with a CV ≈ 0 (Figure 2, both with a mean CV of 0.47). In contrast, the CV distribution of replicate 2 was distinct (P-value < 0.05 to input control and replicate 1) since it had more peaks with a higher CV (mean CV of 1.41) and thus more specific binding events (e.g., Figure 1a, *CV* ≈ 5.3). Yet, some potential binding sites were more unspecific with a CV ≈ 0 (e.g., Figure 1b, *CV* ≈ 0.006).

The CV distribution of the input control was expected, because ideally the control experiment contains no real or not enriched binding events. But, the CV distribution of replicate 1 did not match the assumptions. The library sizes of both replicates were nearly the same with 3.3 – 3.5 million uniquely mapped reads. The distribution was an indicator for a low reproducibility of the binding sites. This was also striking when we compared the distribution of replicate 1 and 2. However, we have to stress out that we are unsure if this is the result of the specificity of SLBP, the different quality of replicate 1 and 2, or a different source, for example, a bad immunoprecipitation. For a downstream analysis, for example the prediction of sequence motifs, it is worth to either exclude replicate 1 or to investigate, why the CV distribution of replicate 1 was very different to replicate 2. Thus given the inspection of StoatyDive, the user can now decide to investigate the binding sites of replicate 1 and compare them with the input control or replicate 2. This helps

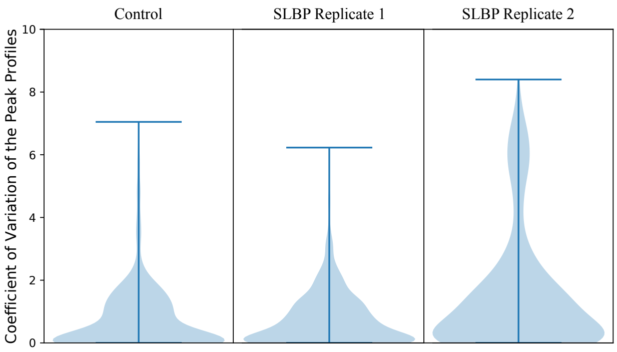

Figure 2. StoatyDive generates a CV distribution to evaluate the peak profile shapes, which can be used as a quality control. The CV distribution of the peak profiles of the input control and replicate 1 of the SLBP CLIP-Seq experiment are quite similar. In contrast, the CV distribution of replicate 2 is different.

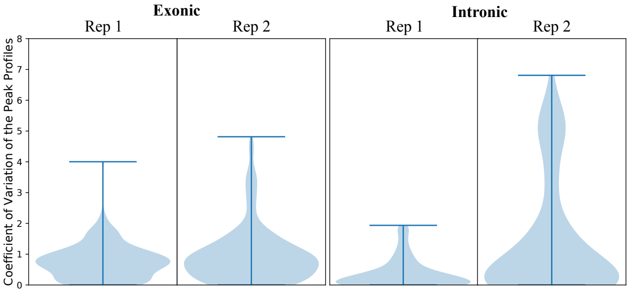

Figure 3. CV distibributions of exonic and intronic peaks reveal a difference in the profile shapes. The CV distributions of the peak profiles of replicate 1 and 2 are more similar in exonic regions than in intronic ones.

to assess if SLBP might have protein domains that bind to RNA in an unspecific manner. The user can also test if the unspecific peak Figure 1b might be a false positive of PureCLIP.

Checking the CV distributions of other CLIP-Seq datasets such as TAF15, TARDBP, and HNRNPA1 (Supplementary Figure 1), which we will analyze further in a later section, we saw that the CV distributions also had differences between the replicates (two-sided Wilcoxon test P-value < 0.05). However, this does not mean a low quality of the data and just highlights that it is important to do replicates in order to quantify biological and technical variance as noted in a previous CLIP study [10].

To investigate further differences between the two replicates, we split the peak set into peaks overlapping with exons and introns (see Figure 3). SLBP is a translation and transport factor, which is present in the cytoplasm as well as nucleus [6, 11]. Therefore, the replicates could have different binding events, where one replicate might have more events in cytoplasm and the other more in the nucleus. For replicate 1 we got 236 exonic and 151 intronic peaks for replicate 1, and 374 exonic and 289 intronic peaks for replicate 2. So replicate 2 had 136 exonic and 136 intronic peaks more. Notably, we can see a huge CV difference when comparing the intronic peaks of the two replicates, with a mean CV of 0.23 for replicate 1 and 1.26 for replicate 2 (one-sided Wilcoxon test P-value < 0.05). The exonic peaks on the other hand were more similar (mean CV = 0.48 and 0.90, respectively), but still the CV distributions were significantly different (one-sided Wilcoxon test P-value < 0.05). It is not clear why this difference appeared, but our observations stress out how important it is to perform an experiment with replicates.

Seven Different Peak Shapes in the SLBP Data

For a more detailed analysis, we classified the peaks of replicate 1 and 2 with the help of StoatyDive (Figure 4). The procedure is mentioned in the methods. StoatyDive has found 7 distinguishable peak profiles for both replicate 1 and 2. We looked more closely at the profiles of replicate 2 (Figure 4b). Cluster 2 and 5, which are set apart clearly from clusters 1, 3, 4, and 6, are characterized by plateau-shaped profiles. The other groups had profiles with mountain-like shapes with peaks tending to become broader and fuzzier in the order of clusters 3, 1, 6, and 4. To return to our initial examples (Figure 1), peak profile Figure 1a was classified by StoatyDive as a small, centered mountain (Figure 4 b3), whereas peak profile Figure 1b was classified as a very broad profile (Figure 4 b4).

It is to mention that constant profiles (Figure 4) represent a constant read coverage throughout the whole peak. Because of the max-min normalization of the profile (see Methods), the value becomes 0, that is to say, the profile is not empty. In contrast, peaks shaped like plateaus have not a constant value since their coverage changes at a few positions. Furthermore, the number of clusters depend on the optimization of StoatyDive, but can also be defined by the user. Other proteins, experimental conditions, and methods might have different peak profile groups. Even in our example it is worth to investigate if cluster 4 and 6 of replicate 2 can be separated more distinctly. To do this, one could run StoatyDive again, using only the peaks of cluster 4 and 6.

The high distance between cluster 2 and 5 might have been the result of the difference between the profile borders (compare Figure 4 b2 and b5). Where cluster 2 had profiles with lots of values in the left or right side of the peak profile, cluster 5 has occupied the center of the peak profile.

A broader and fuzzier profile might not necessarily mean that it was an unspecific site. Perhaps some of them were just a collection of several specific peak profiles that were merged together. This could have happened because of the peak caller model or the peak correction and extension of StoatyDive. It is

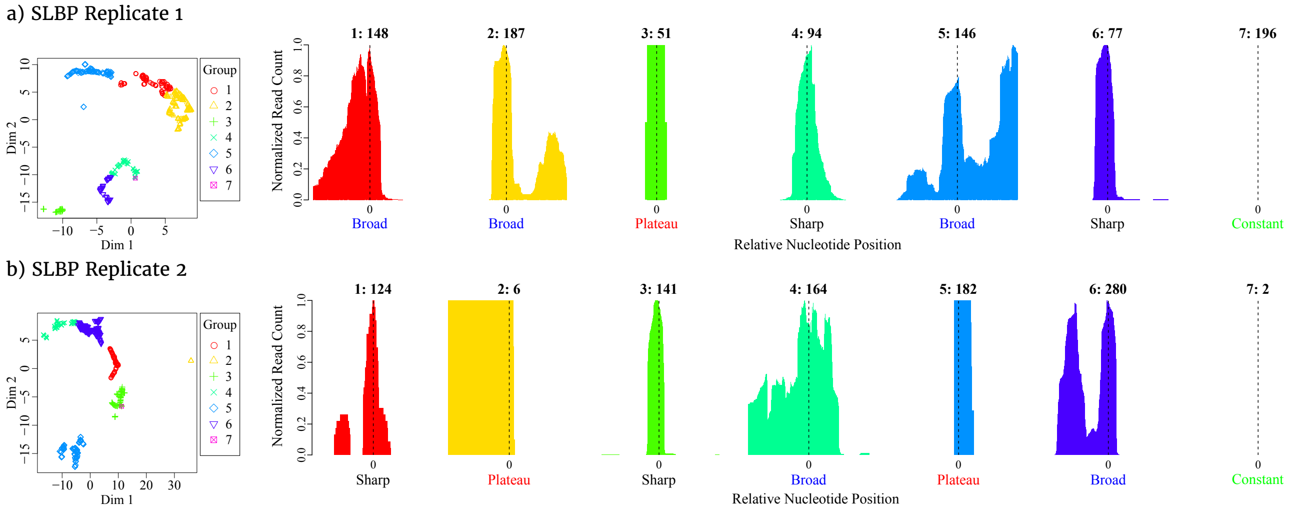

Figure 4. Results of the peak profile clustering with StoatyDive (procedure described in the methods). We applied StoatyDive to the SLBP data [5]. StoatyDive has found 7 different peak profile shapes in the data of replicate 1 (a1–7) and replicate 2 (b1–7) of SLBP. We present one example profile for each cluster with the number of peaks on top. For replicate 1 and 2 we could separate between very thin and specific mountains such as Figure 1a and very broad profiles like Figure 1b. We also found peaks shaped like plateaus, such as 3a, and constant peaks, for example, 7a. The profiles also vary slightly between groups. For example, 6b has more than one spiky mountain in contrast to 1b.

Table 1. Number of peaks of SLBP for different shape groups.

Replicate	Total	Sharp	Broad	Plateau	Constant
1	899	171	481	51	196
2	899	265	444	188	2
	Peak Summits in Histone mRNAs				
1	116	22	86	6	2
2	118	42	71	5	0

worth to take these profiles and reduce the extension, and in addition run a peak deconvolution. It is important to note that peaks shaped like plateaus might be false positives.

SLBP has been reported as an mRNA export and translation factor [6]. Thus, it is worth to investigate if peaks like Figure 1a are more informative for a translation factor than peaks like Figure 1b. That is to say, Figure 1a might be more suited for sequence and structure predictions than peak Figure 1b. Therefore, we will do a deeper inspection of group 1, 3, 4 and 6 later in the paper.

We also checked the uniqueness of the shapes by analyzing the peaks based on the reads from the size-matched input control (see Supplementary Figure 2). StoatyDive had just identified 4 different clusters, encompassing mountain shaped peaks, as well as plateaus, and constant peaks. It was to be expected to find similar shapes in the control, because the biggest challenge for peak-calling is the identification of enriched sites with different shapes between control and CLIP data [12, 13]. In a future version of StoatyDive, we will include a mode to check peak shapes between samples to see if we could improve peak-calling results with a peak shape comparison.

Information from Peak Profile Shapes

We made the assumption that replicate 1 might have more unspecific and less distinguishable profiles than replicate 2 based on the different CV distributions (Figure 2). Thus, we counted the number of peaks in each cluster for replicate 1 and 2 (Table 1). From our robust 899 peaks, in replicate 1 we had $\approx$ 19% peaks being a sharp mountain shape (Figure 4 a4 and a6), $\approx$ 53% being a broader mountain (Figure 4 a1, a2 and a5), $\approx$ 6% peaks with plateaus (Figure 4 a3), and $\approx$ 22% constant shaped peaks (Figure 4 a7). replicate 2, on the other hand, had $\approx$ 29% sharp mountain shaped peak profiles (see Figure 4 b1 and b3), so 94 more than replicate 1. This corroborated the assumption that replicate 1 had more broader and unspecific sites. Thus, replicate 2 had only $\approx$ 49% broad peak profiles (Figure 4 b4 and b6), and only 2 constant peak profiles (Figure 4 b7). Yet, replicate 2 had $\approx$ 21% peaks with plateaus (Figure 4 b2 and b5).

We further investigated the biological function of different peak profiles of replicate 2. Since SLBP targets histone mRNAs [6], we intersected known annotated mRNAs of histones with the peaks of the different profile clusters (Table 1). From the 899 peaks, only $\approx$ 13% of replicate 2 overlapped with mRNAs of histones. Yet, of these 118 peaks almost all came from group 1, 3, 4, and 6. These groups were either spiky, or broader mountain-shaped peak profiles. For example, we found a sharper peak located on RNU7-1 RNA (U7 small nuclear 1) that contains a stem loop that might be potentially targeted by SLBP [14]. The peak got a CV of 3.9 and was classified into the peak profile cluster 3 of replicate 2. Only 5 peaks intersected with histone mRNAs that had a profile shaped like a plateau (Figure 4 b5). This endorsed the assumption that peak profiles shaped like plateaus were less informative. The observation also suggests that broader profiles were still informative, because some of them overlapped with histone mRNA.

Next, we used MEME-ChIP [15] to search for sequence motifs associated with the different peak shape groups of the second replicate of SLBP. We have found 2 significantly enriched motifs associated with the plateau peaks and 3 motifs associated with the sharper peaks (Table 2). Yet, both the plateaus and the sharper peaks had two similar sequences motifs. Both motifs *(G)GCUCUUU(U)* and *(CA)GAGCCA(C)* were higher enriched in the sharper shaped peaks. On the other hand, we found > 10 enriched motifs for the broader shaped peaks. The motifs of that peak set were very different to the motifs of the plateaus and sharper shaped peaks. Even the first 3 significantly enriched motifs had more noise and consequently were less enriched than the motifs of the other 2 peak shape groups. The E-values of all motifs were also > 1000 times higher for broader peaks than for sharper peaks. Peaks shaped like plateaus were slightly more significant than broader peaks.

On further inspection of those peak motifs in histone mRNAs, we found that the 3 motifs of the sharper shape peaks covered 10 more histone mRNAs (49 in total) than the broader shaped peaks (39 in total). This endorsed the observation that broader peaks encompassed more noise. For example, the second motif of the broad peaks *CA(A/C)CAAG* came close to the third sequence motif, with the sequence *A(C/A)CCAAAG*, of the sharper shape peak group. The second motif had the highest occurrence in histone mRNAs for the broader peaks. Thus, the broader peak set might include true binding sites but with some higher additional noise. Furthermore, we already showed that the plateau group might also hold some peaks that are true binding sites (Table 1), which was confirmed by the similar sequence motifs to the sharper peak set. We could confirm that the 2 sequence motifs that are present in plateaus were also present in histone mRNAs (Table 1). All in all, just the sequence motifs analysis showed how different the outcome of subsequent tasks can be for different peak shape groups. Yet, we cannot confirm the biological truth behind those motifs as it requires further experiments to verify them.

Optimizing StoatyDive with the data of SLBP

We investigated the peaks of the second replicate of SLBP further and took the CLIPper peaks (ENCFF127WAK) from the study by Van Nostrand et al. [5]. We wanted to check the specificity and sensitivity of StoatyDive for different CV cutoffs and peak sizes (see Supplementary Table 3). PureCLIP does not give a FC or P-value, so it was only possible to calculate those features with the CLIPper peaks. Since SLBP binds mainly his-

Table 2. First 3 MEME-ChIP motifs for the different peak shape groups of SLBP with the E-value, the portion of sequences that have the motif, and the number of peaks that have the motif and which also intersect with histone mRNAs.

Shape	Motif 1	Motif 2	Motif 3
Broad	3.5e−4; 5.85%<br>13<br>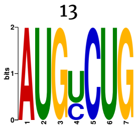	1.5e−3; 5.40%<br>18<br>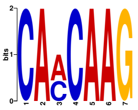	1.5e−3; 5.40%<br>8<br>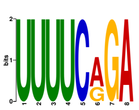
Sharp	5.7e−12; 20.00%<br>23<br>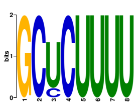	8.6e−9; 18.87%<br>17<br>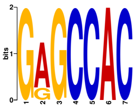	1.0e−3; 9.06%<br>9<br>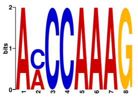
Plateau	1.6e−5; 15.42%<br>3<br>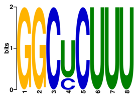	1.6e−3; 12.23%<br>2<br>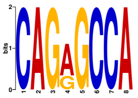	

tone mRNAs [6], we defined peaks in histones with a $\log_2$ fold change (LFC) >= 1 and a P-value < 0.05 as true positives. A true negative was a non-significant peak in a region that did not overlap with a histone. We investigated only peaks where we could calculate a CV and used StoatyDive with a peak size of 30 (median), 40 (Q3), 70 (Q3 + 1.5×IQR) and a maximum peak length of 201 nucleotides. The peak sizes were chosen based on the length of all peaks (see Supplementary Figure 4). The Matthews correlation coefficient (MCC) is more informative in case of imbalanced datasets, which was the case of the SLBP data. The true negative (TNR) was always at 1.0 because the peak set had no peaks in histones that were not significant (no false positives). We investigated the cluster with the highest (Main Cluster) and second highest (Second Cluster) number of peaks in histones. Looking solely at the clustering, we achieved the highest true positive rate (TPR) (0.69) and MCC (0.66) with a peak length of 70 than does the set of all CLIPper peaks with a TPR of 0.48 and MCC of 0.57. This was achieved by the second cluster, which pointed out that the cluster had to be carefully chosen in order to remove some noise in the data (artifacts). The cluster had also the highest enrichment with a mean LFC of 3.4 (median P-value < 0.05) than does all CLIPper peaks with a value of 1.9 (median P-value = 0.038). Furthermore, the same size achieved the highest TPR (0.61) and MCC (0.68) with a CV threshold of 0.2. However, the enrichment was not as good as with the clustering resulting in a mean LFC of 1.6 (median P-value = 0.108). Based on these different sets, we observed that the CV was sometimes lower in the main or second cluster. We concluded together with the previous results that broad and sharp peaks equally play an important role for SLBP.

Comparison to Existing Tools

To further validate StoatyDive, we applied 2 other peak shape clustering tools, namely FunChIP (Parodi et al. 16; version 0.99.4) and SIC-ChIP (Cremona et al. 17; current release), to the second replicate of SLBP. To have a better ground truth, we took 10 peaks of three different peak shape groups (broad, sharp, and plateau) to define a test set with three distinct peak shapes from real CLIP-Seq data (in total 30 peaks). We strictly used the output of the tested tools. Both tools were designed and tested for ChIP data. A peak shape clustering was so far not done for CLIP data and a specific tool for that data type did not exist to the best of our knowledge. We applied SIC-ChIP with $N = 10$ and $toll = 10$ (the default parameter set resulted in errors) and ran FunChIP according to the manual in Bioconductor with the smoothing parameter $lambda = 10^3$. StoatyDive classified most peaks correctly into the 3 peak shape groups (Figure 5a, accuracy (ACC) = 0.87). It sorted 4 peaks incorrectly that came from the broad and sharp peak group. Sharp and broad peaks are harder to cluster and a second factor such as the CV helps to give a final assessment over the shape of the peak. SIC-ChIP identified up to 6 different peak shapes (Figure 5b), whereas FunChIP found 3 (Figure 5c and d, ACC = 0.6). Furthermore, SIC-ChIP as well as FunChIP had clusters that are mixed and not as well separated as with StoatyDive. SIC-ChIP's predefined shape indices were not enough to separate the peak shape profiles properly, as shown for one scatter plot (Figure 5b) with the highest explained variance (cluster separation). In turn, FunChIP performed slightly better, finding profiles with different summit intensities. However, for the smoothed (Figure 5c) as well as the smoothed and scaled profiles (Figure 5d) the clusters included a lot of profiles with different shapes. For example, cluster 2 and cluster 3 of the smoothed profiles seemed very similar. Thus, FunChIP's approach to use the whole profile without any predefined features or dimensional reduction was also not enough to separate the peak shapes in the same way as with StoatyDive.

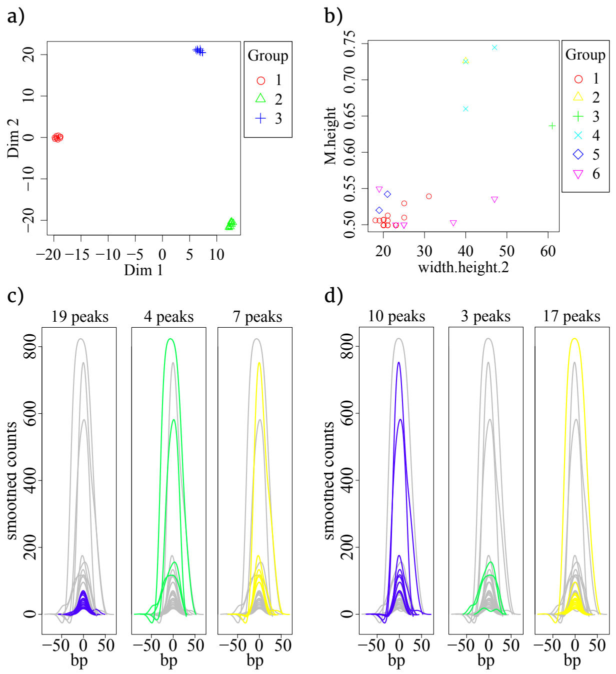

Figure 5. Peak shape clustering with StoatyDive, SIC-ChIP [17], and FunChIP [16] on a set of selected peaks from 3 different peak shapes of the second replicate of SLBP. (a) StoatyDive successfully identified the 3 distinct peak shape groups with 10 peaks each, but a few peaks were sorted incorrectly (ACC = 0.87). (b) From the 5 shape indices of SIC-ChIP (see Supplementary Figure 5), we picked one scatter plot (b) with the highest explained variance ($w_{h/2}$ vs $\frac{M}{h}$) to show the clustering of SIC-ChIP. FunChIP was only able to identify 3 distinct clusters (ACC = 0.6). The (c) smoothed and (d) smoothed and scaled profiles were clustered mostly on the intensity of the summit.

Investigation of eCLIP Protein Profiles

We further investigated the peak shapes of several proteins from the study of Van Nostrand et al. [5], namely: CPSF6, CSTF2T, EWSR1, LARP7, RBM22, SAFB2, SLBP, SLTM, TAF15, TRA2A, U2AF1, HNRNPA1, IGF2BP1, IGF2BP2, NONO, SRSF1, TARDBP, HNRNPM, U2AF2, and PTBP1. We took the robust peaks (peak-calling, IDR, signal normalization) and the bam files, which were used for the peak-calling, from each protein from the ENCODE database. We chose the data from the eCLIP experiment on K562 and focused on proteins where the biological and molecular function and the number of RRMs are clearly listed on UniProt. Thus, we wanted to investigate if the number of RRMs or the function of the protein by any means affected the shape of the peak profiles and consequently led to more or less broader peaks. We therefore took the files from ENCODE and merged both bam files (replicates) for the coverage. We then used StoatyDive with *--peak_correction --scale_max 10 --border_penalty --sm --peak_length* 77 *-k* 3. All peaks were therefore extended or shrunk to a length of 77 nucleotides. This was based on the observation that the third quartile of all peaks from all proteins was 77 nucleotides long (see Supplementary Figure 4). In addition, StoatyDive achieved for the SLBP data a better TPR and MCC with a peak size of 70 and a CV threshold of 0.2 (see Supplementary Figure 3). The results of SLBP showed that it may be wise to combine the clustering and the CV threshold to assess the profile landscape of other proteins. We therefore defined a peak as sharp if it had a CV > 0.2 and it

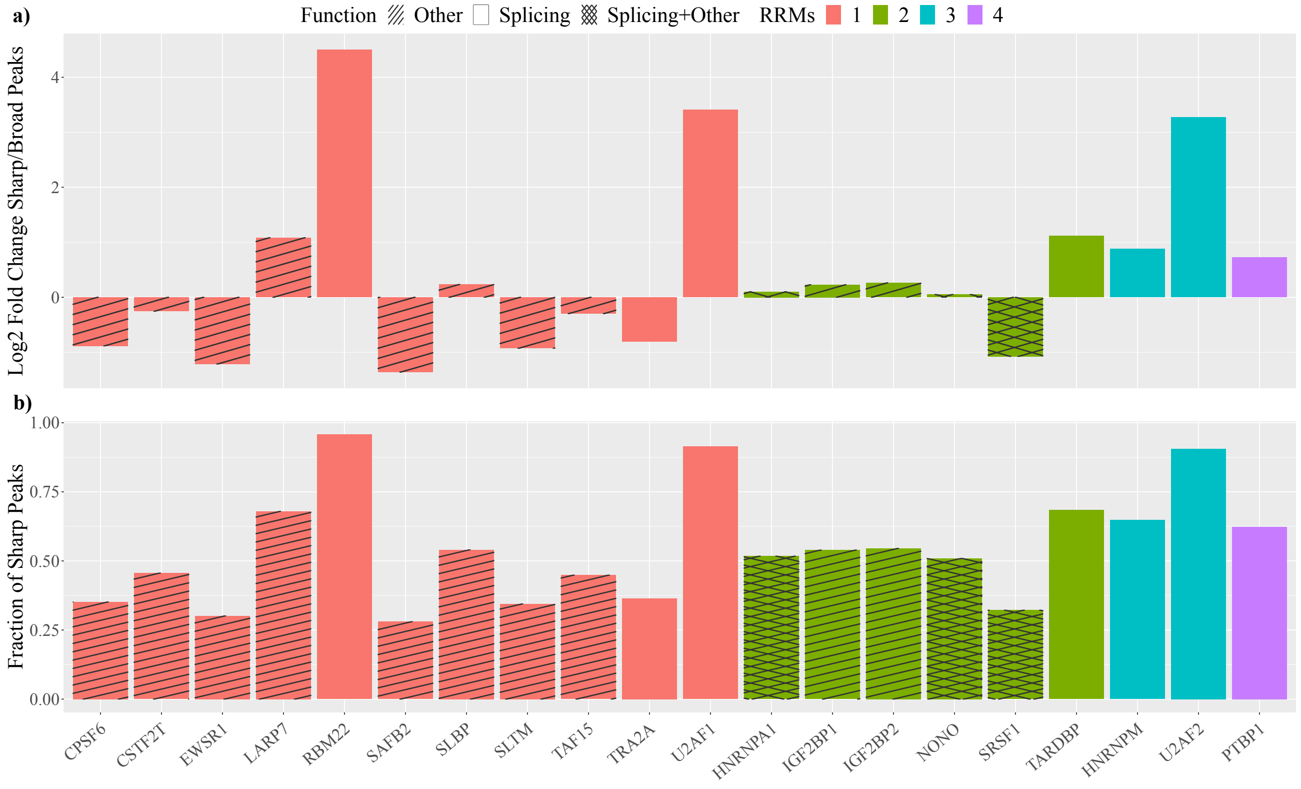

Figure 6. (a) Log_2 fold change of the number of sharp peaks versus the number of broad peaks; (b) fraction of sharp peaks. UniProt lists the proteins CPSF6, CSTF2T, EWSR1, LARP7, RBM22, SAFB2, SLBP, SLTM, TAF15, TRA2A, and U2AF1 with one RRM and the proteins HNRNPA1, IGF2BP1, IGF2BP2, NONO, SRSF1, TARDBP, HNRNPM, U2AF2, and PTBP1 with at least two RRMs. Proteins with more than one RRM tend to have more sharper shaped peaks, but also equally have more broader peaks. The proteins RBM22, U2AF1, TARDBP, HNRNPM, U2AF2, and PTBP1 had a higher number of sharper peaks and all of them are involved in RNA splicing (colored bars). Other proteins with different functions (stripes) often have less sharper peaks.

fell into a cluster that was generally sharper. A cluster was declared as sharp if the median CV of the cluster was bigger than the median CV of the whole peak set. All other peaks were classified as broad.

The proteins LARP7, RBM22, SLBP, U2AF1, IGF2BP2, NONO, TARDBP, HNRNPM, U2AF2, and PTBP1 had a higher number of sharper shaped peaks, whereas the rest of the proteins had a higher number of broader shaped peaks relative to the other shape (Figure 6a). Protein IGF2BP1 was almost half sharp and half broad peaks. UniProt lists the proteins CPSF6, CSTF2T, EWSR1, LARP7, RBM22, SAFB2, SLBP, SLTM, TAF15, TRA2A, U2AF1 with one RRM and the proteins HNRNPA1, IGF2BP1, IGF2BP2, NONO, SRSF1, TARDBP, HNRNPM, U2AF2, PTBP1 with at least two RRMs.

We could observe a trend between the number of RRMs and the number of sharper shaped peaks (Figure 6a). From 11 proteins with 1 RRM just 4 had more sharper peaks than broader shaped peaks and 8 out of 9 proteins with at least 2 RRMs had sharper shaped peaks. Furthermore, the proteins HNRNPM, U2AF2, and PTBP1 have all more than 2 RRMs (3, 3, and 4, respectively), which led to the assumption that an increasing number of RRMs results in more sharper peaks. Figure 6b shows more clearly that proteins with more than one RRM tend to have more sharper peaks. It is possible that RNA-proteins interactions become more specific with > 1 RRM and so the separation between more specific and unspecific binding sites is stricter. At this point, we had not taken any other RNA binding domains into account apart from RRMs.

Another observation was that shapes of splicing factors (RBM22, U2AF1, TARDBP, HNRNPM, U2AF2, and PTBP1) tend to be more sharper than broader (Figure 6a). Yet, proteins, such as SRSF1, had more broader peaks and are also involved in splicing. On the other hand, proteins not involved in splicing such as EWSR1, SAFB2, SLTM, and TAF15 clearly showed more broader shaped peaks. The proteins SLBP, HNRNPA1, IGF2BP1, IGF2BP2, and NONO almost had an equal number of sharper or broader shaped peaks, and all these proteins have multiple functions, contributing to at least two biological processes, such as transport and translation in the case of SLBP [6]. SRSF1 is also a multi-functional protein. Perhaps, that is the reason why it has more broader peaks even though it is a splicing factor. We also have to consider a combination of both factors, the number of RRMs and the biological function, which is a question for the future, because also technical factors might play a role for the peak shapes. For example, the number of broader and sharper shaped peaks of the protein SLBP was different from the previous results (Table 1). This can be the effect of a different peak caller (the study of Van Nostrand et al. [5] used CLIPper) or the different data processing steps.

We also checked whether our result that RBPs involved in splicing have sharper peaks can have technical reasons. In this case, a splicing related protein could have more peaks that are split over two exons, which are detected by the peak caller as two separate but sharp peaks (split peak). So we investigated the number of peaks that fall into introns (90% overlap) for the proteins that are involved in the splicing process. We used bedtools set to a strict overlap (intersect -u -s -f 0.9) to investigate potential split peaks. The proteins PTBP1 ($\approx$ 85%), RBM22 ($\approx$ 62%), TARDBP ($\approx$ 79%), and HNRNPM ($\approx$ 87%) had more than 50% of peaks in introns, which deflected the assumption of split peaks. However, the proteins U2AF1 ($\approx$ 17%), and U2AF2 ($\approx$ 15%) had more peaks in exon regions, where the possibility of split peaks might still occur. It is important to note that this does not mean that the aforementioned proteins

bind generally more introns or exons. As there exist no tool that can correct for split peaks, a further analysis for these proteins was required. We checked for potential split peaks by extending the peaks that fall completely into exons by 5 nucleotides to each side. Next, we intersected those extended peaks with introns to see if they are close to the exon boundaries. We found for U2AF1 only 27 peaks (0.72%) and for U2AF2 5 peaks (0.40%) that are potential split peaks, again deflecting the assumption of a technical artifact in the peak set of splicing factors. Thus, the sharpness of the peaks of U2AF1 and U2AF2 was potentially not the result of the peak-calling.

Potential Implications

StoatyDive is a powerful tool that can evaluate and classify peak profiles. It can be used in any sequencing data analysis that involves the prediction of binding sites such as CLIP-Seq, or ChIP-Seq. Within this work, we provided an example for SLBP to show the usability of StoatyDive. First, it is possible to assess the quality of an experiment such as CLIP. The CV is just one quality factor and we recommend to test other features as well, such as the read coverage correlation. Second, StoatyDive assists to evaluate the binding specificity of the protein. The normalized CV distribution produced by StoatyDive provides valuable information for the user. A protein that binds very specific will have a distribution concentrated around a normalized CV of one. A protein with a lot of unspecific bindings will have a normalized CV distribution ≈ 0. Third, StoatyDive helps to filter for specific and unspecific binding sites to investigate if the protein has multiple protein domains that have different binding mechanisms. A finer distinction can be made with the classification mode of StoatyDive. This helps to identify peak profiles with a specific shape and filter them based on the corresponding biological question and function of the protein. For example, a transcription factor might have more specific bindings (more spiky mountains), than a protein complex or a helicase (more broader mountains). Fourth, the results of StoatyDive can be used to validate a peak caller (e.g., PureCLIP), that is to say, one can assess how many false positives are in the peak sets based on the shape. Different peak caller might result in disparate peak sets and consequently different peak profile shapes.

StoatyDive is a very powerful, well documented, and easy to apply tool that refines the binding site detection in the data analysis such as CLIP-Seq. Nevertheless, StoatyDive is a very general tool. In the future it is worth to investigate, if StoatyDive can be used with different types of peak-calling outputs and data types of sequencing data (e.g., ChIP-Seq, ATAC-Seq, Ribo-Seq, and others). It serves as a quality control and filtering step to select specific binding profiles, which therefore allows to improve other binding site prediction tools such as DeepBind [18], or any other subsequent analysis tasks, to increase the accuracy for the prediction.

Methods

Peak Correction, Extension and Coverage Calculation

StoatyDive was implemented in python (>= 3.6) and R (>= 3.4.4). The tool needs three files: the predicted binding regions of a peak-calling algorithm in bed6 format, a bam or bed file that was used for the peak-calling (experiment or control), and a tabular file of the chromosome size of the reference genome (Figure 7).

First, StoatyDive checks if a peak profile needs to be centered (peak correction). In the default mode, the profiles are centered by a convolution with a standard normal distribution. The maximum value of the convolution gives the nucleotide shift of the peak profile to center the peaks. So the window with the peak length is shifted to the center of the peak (Figure 7 step 1). With this approach we retain the context and take care of two problems. First, peak callers often produce peaks that are not correctly centered. Second, dimensionality reduction methods, such as uniform manifold approximation and projection for dimension reduction (uMAP; McInnes et al. 19), are not translation invariant. Thus, two profiles with the same shape but in a different relative genomic position might end up in different locations in the new dimensional space.

After the peak correction, StoatyDive extents the peaks by default to the maximal peak length of the given peak set (Figure 7 step 2). This removes the peak length as a potential feature for the evaluation and classification. StoatyDive then calculates the read coverage (Figure 7 step 3) for each position inside a peak with the help of bedtools [9].

Evaluation of Peak Profiles

With the results of bedtools, StoatyDive evaluates every peak i from the total set of k peaks. StoatyDive will estimate the read count for every peak as a negative binomial $X_i \sim \mathrm{NB}(r_i, p_i)$ with the hyperparameters r_i (number of hits) and p_i (probability of a hit). It then calculates the coefficient of variation (CV) for every peak. A simple estimation of the variance is not enough because the profile depends on the read coverage. Thus, to be able to compare each peak profile we have to normalize for the expected number of reads to adjust the variance. So the CV for each peak,

$$CV_i = \sqrt{\frac{1 - p_i}{r_i}}, \qquad (1)$$

is calculated with the estimated hyperparameters. In the last step, StoatyDive normalizes the CV score by the max and min of all scores,

$$CV_i' = \frac{CV_i - \min(\forall CV)}{\max(\forall CV) - \min(\forall CV)}. \qquad (2)$$

At the end, our defined CV score will range from $CV_i = [0, \infty]$ and the normalized score from $CV_i' = [0, 1]$, with a $CV_i' = 0$ for a more unspecific binding and $CV_i' = 1$ for a more specific one.

Classification of Peak Profiles

StoatyDive classifies the peak profiles in an unsupervised manner using uMAP [19] and k-means clustering [20]. Yet before clustering, StoatyDive processes the peak profiles. First, the profiles are normalized based on the individual maximum and minimum read count, since we are only interested in the shape of the profiles and not in the absolute read counts (Figure 7 step 4). So assuming each peak X_i has $x_1, x_2, x_j \ldots, x_n$ nucleotides, we normalized the peaks by $x_j = \frac{x_j - \min(X_i)}{\max(X_i) - \min(X_i)}$. Second, the peak profiles are smoothed (Figure 7 step 4) with a spline regression [21]. The step reduces the noise for each profile and distributes the data more uniformly on the current manifold. The latter is important since it is the data assumption of uMAP. StoatyDive further adds curve specific features to the processed peak profiles including: the number of maximal values, the area under the curve, and the arc length. StoatyDive applies uMAP to the final data with 5,000 epochs, 2 components (dim = 2), a minimum distance of 0.01 and a size of the local neighborhood of 5. The original and high dimensional profiles often suffer from

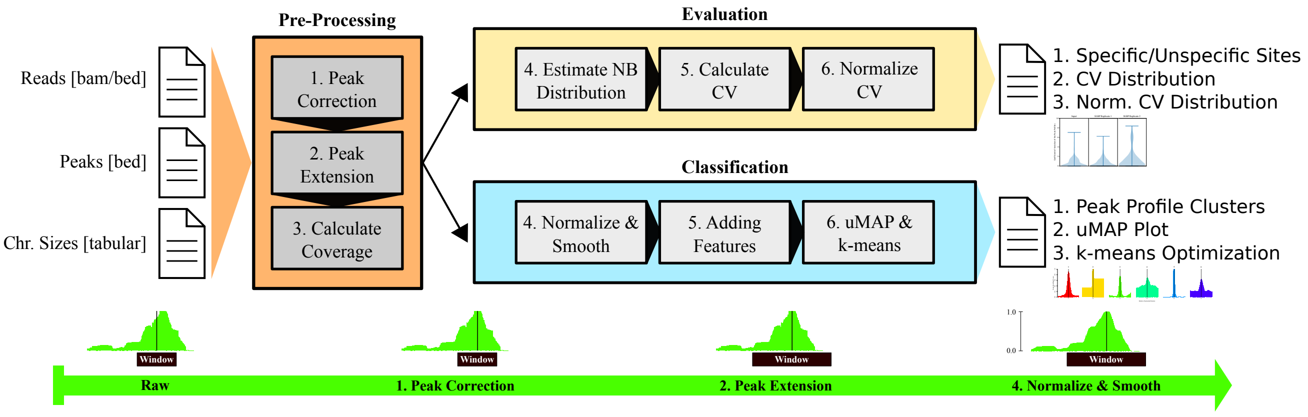

Figure 7. Overview of the StoatyDive pipeline. It consist of 2 major modules, namely the evaluation and the classification of peak profiles. The user has to provide reads (or events), peaks and a chromosome size file. StoatyDive then shifts the peaks to their correct center (peak correction), extents the peaks to a common length (maximal peak length of peak set or user defined value), and calculates the coverage with bedtools [9]. The peak correction can be turned off. In the evaluation, StoatyDive then estimates the read coverage as a negative binomial. From the hyperparameters it calculates the coefficient of variation (CV) and normalizes it (Equations 1 and 2). The normalized CV can then be used to divide the peaks into specific and unspecific sites. Furthermore, the CV distribution acts as a quality control between control and signal experiments. In the classification, StoatyDive first normalizes the peak profiles to remove the intensity as a feature. Then it smooths the profiles to support the data assumptions of uMAP [19] and to remove some noise. After that, it adds curve specific features to the data. The higher dimension of the data is then reduced with uMAP. StoatyDive then clusters the new data with k-means [20]. The user then obtains several plots and a table to investigate the different peak profile clusters.

the curse of dimensionality which would lead to a higher number of individual clusters. The dimensional reduction was optimized with some test data comprising four different sets of distributions: a uniform distribution, a linear distribution, an unimodal Gaussian distribution, and a bimodal Gaussian distribution. Subsequently, StoatyDive applies k-means clustering to the new data with 100 initializations, and maximal $10,000$ iterations. The number of clusters k is found by convergence of the total within-cluster sum of squares and checked with the Akaike information criterion (AIC; Akaike 22). We also tested other dimensionality reduction methods (see Supplementary Figure 6) such as principal component analysis (PCA), a self-organizing map (SOM), and t-Distributed Stochastic Neighbor Embedding (t-SNE). However, none of them came close to the results of uMAP.

Output of StoatyDive

For the peak evaluation, StoatyDive generates a plot of the CV (Equation 1) and normalized CV (Equation 2) distribution (Figure 7). The user receives a first impression of the binding specificity of the protein of interest from the CV distribution. An unspecific binder has a CV distribution ≈ 0. A more specific binder has a CV distribution ≥ 1. The CV distribution can also be used as a quality control to compare control and signal experiments. A quality breach might have occurred if the distributions of the control and signal experiment almost look identical. A control experiment should normally have a CV distribution ≈ 0, with only a very few binding sites showing higher CVs.

The normalized CV distribution helps to evaluate the peaks based on the individual experiments. An empirical threshold is set at a CV of 0.2 (Equation 1), below which binding sites are deemed unspecific.. The user can change the threshold. Keep in mind, the threshold for the normalized CV is relative in accordance to the individual experiment.

For the peak classification, StoatyDive generates a plot of the k-means optimization and a plot of the dimensional reduction with uMAP, which can be used to readjust the number of k clusters if this is necessary. The user also receives a set of example peak profiles and smoothed peak profiles of each cluster, which can be used to investigate the identified shapes. For a general trend, StoatyDive delivers average profiles for each cluster.

The final output of StoatyDive is a CV sorted table of the whole peak set, from the highest to the lowest CV. Each peak is labeled with 0, for more specific binding sites, and 1, for more unspecific sites. The table also lists for each peak the cluster number (group number) of the peak profile shape.

Important Options of StoatyDive

The peak correction (Figure 7 step 1) can be turned off. The user can also change the translocation scheme of the peak profiles to shift them based on the maximal value (summit). The maximum translocation scheme is useful for nucleotide specific events such as truncation events in the case of iCLIP data [23]. StoatyDive has also the option for a different CV score that penalizes peaks within broad plateaus. StoatyDive then adjusts the CV score of peaks that are covering a small appendage of a read stack. Furthermore, the user can provide a maximal score to StoatyDive to normalize the CV distribution (Equation 2). This option helps to compare the CV distribution between experiments in accordance to their disparate peak sizes and total amount of reads. StoatyDive also has a threshold for the normalized CV score to divide the peaks into more specific and more unspecific binding sites, which the user can change.

StoatyDive has two major parameters for the peak profile classification (Figure 7 step 6). First, the user can adjust the maximal amount of potential peak clusters identified by the k-means clustering. Yet, the final number of peak clusters will be optimized by StoatyDive. The parameter is an upper bound. However, the user has the option to force StoatyDive to use k specific clusters. The smoothing (Figure 7 step 4) of the peak profiles can also be adjusted by the user. The default was optimized with different test sets. Increasing the parameter (> default) might underfit the smoothing and thus lead to fewer peak clusters. A lower value (< default) might overfit and so lead to more clusters. The smoothing can also be turned off, but it is recommended to turn it on.

Availability of Supporting Source Code and Requirements

Project name: StoatyDive
Project home page: https://github.com/BackofenLab/StoatyDive
Conda: https://anaconda.org/bioconda/stoatydive
Operating system(s): Unix
biotools:StoatyDive
RRID:SCR_018796

Availability of Supporting Data and Materials

StoatyDive provides a small dataset for a test run, which can be found in the github repository. The whole eCLIP data used in this paper, such as SLBP or RBFOX2, is listed in the supplementary of the study by Van Nostrand et al. [5].

Additional files

Supplementary Figure 1. CV distributions of all other proteins analyzed for figure 6 with two-sided Wilcoxon test P-value. The two replicates quite often have different CV distributions.

Supplementary Figure 2. We applied StoatyDive to the size matched input control of the SLBP data [5]. StoatyDive has found 4 different peak profile shapes, broad (cluster 1), plateau (cluster 2), sharp (cluster 3), and constant (cluster 4). The supplements also include the average profiles for replicate 1 and 2 to show the overall trend of the clusters.

Supplementary Table 3. Mean CV, variance of the CV, mean $\log_2$ fold change (LFC) enrichment between the control and CLIP experiment, median P-value, true positive rate (TPR), true negative rate (TNR), accuracy (ACC), and Matthews correlation coefficient (MCC) for the analyzed peaks (All Peaks) of replicate 2 (ENCFF127WAK) from the study by Van Nostrand et al. [5]. Features are listed for the peak shape cluster with the highest number of peaks in histones (Main Cluster) and second highest number (Second Cluster), and for the peaks with a CV smaller or bigger a threshold of 0.2, 0.5, and 0.8, using different peak lengths (30, 40, 70, and maximum peak length of 201 nucleotides). We achieved the best TPR, ACC and MCC with a peak length of 70 and with a CV cutoff of 0.2.

Supplementary Figure 4. Peak lengths of the peak set (ENCFF127WAK) for the second replicate of SLBP and peak lengths of all other proteins of the eCLIP data from the study by Van Nostrand et al. [5].

Supplementary Figure 5. All scatter plots from SIC-ChIP [17] for the artificial SLBP data.

Supplementary Figure 6. We tested different dimensional reduction methods such as PCA, SOM, and t-SNE on the CLIP data of SLBP. The PCA has no clear clusters for replicate 2, which is similar for t-SNE on replicate 1 and 2. Using an optimized SOM delivers a feature layer with a very high activated hidden unit for replicate 2. It is hard to see any distinct clusters from the counts (activation) of each hidden unit. uMAP can clearly separate the data into more defined clusters. Furthermore it is much easier to interpret the results of uMAP, whereas an artificial neural network, such as a SOM, generates a feature layer (hidden layer) that is hard to explain.

Declarations

List of abbreviations

ACC: Accuracy; CLIP-Seq: Crosslinking immunoprecipitation in combination with high-throughput sequencing; CV: Coefficient of variation; LFC: $\log_2$ Fold Change; MCC: Matthews correlation coefficient; PCA: Principal component analysis; RBP: RNA-binding proteins; RRM: RNA recognition motifs; SOM: Self-organizing map; TPR: True positive rate; TNR: True negative rate; t-SNE: t-Distributed Stochastic Neighbor Embedding.

Ethical Approval

Not applicable

Consent for Publication

Not applicable

Competing Interests

The authors declare that they have no competing interests.

Funding

This study was funded by the Deutsche Forschungsgemeinschaft (DFG, German Research Foundation) grant 322977937/GRK2344 2017 MeInBio – BioInMe Research Training Group, and Germany's Excellence Strategy (CIBSS - EXC-2189 - Project ID 390939984).

Author's Contributions

F.H. performed the computational analysis and tool developement. R.B. initialized the project, and supervised the research. F.H. and R.B. wrote the manuscript. All authors read and approved the final manuscript.

Acknowledgements

We are grateful to Gokcen Eraslan for his support.

References

1. Lee FC, Ule J. Advances in CLIP technologies for studies of protein-RNA interactions. Molecular cell 2018;69(3):354–369.
2. Jankowsky E, Harris ME. Specificity and nonspecificity in RNA–protein interactions. Nature reviews Molecular cell biology 2015;16(9):533–544.
3. Müller-McNicoll M, Neugebauer KM. How cells get the message: dynamic assembly and function of mRNA–protein complexes. Nature Reviews Genetics 2013;14(4):275.
4. Corcoran DL, Georgiev S, Mukherjee N, Gottwein E, Skalsky RL, Keene JD, et al. PARalyzer: definition of RNA binding sites from PAR-CLIP short-read sequence data. Genome biology 2011;12(8):R79.
5. Van Nostrand EL, Pratt GA, Shishkin AA, Gelboin-Burkhart C, Fang MY, Sundararaman B, et al. Robust transcriptome-wide discovery of RNA-binding protein binding sites with enhanced CLIP (eCLIP). Nature methods 2016;13(6):508.

6. Sullivan KD, Mullen TE, Marzluff WF, Wagner EJ. Knockdown of SLBP results in nuclear retention of histone mRNA. Rna 2009;15(3):459–472.
7. Dobin A, Davis CA, Schlesinger F, Drenkow J, Zaleski C, Jha S, et al. STAR: ultrafast universal RNA-seq aligner. Bioinformatics 2013;29(1):15–21.
8. Krakau S, Richard H, Marsico A. PureCLIP: capturing target-specific protein–RNA interaction footprints from single-nucleotide CLIP-seq data. Genome biology 2017;18(1):240.
9. Quinlan AR, Hall IM. BEDTools: a flexible suite of utilities for comparing genomic features. Bioinformatics 2010;26(6):841–842.
10. Chen X, Chung D, Stefani G, Slack FJ, Zhao H. Statistical issues in binding site identification through CLIP-seq. Statistics and Its Interface 2015;8(4):419–436.
11. Erkmann JA, Wagner EJ, Dong J, Zhang Y, Kutay U, Marzluff WF. Nuclear import of the stem–loop binding protein and localization during the cell cycle. Molecular biology of the cell 2005;16(6):2960–2971.
12. Uhl M, Houwaart T, Corrado G, Wright PR, Backofen R. Computational analysis of CLIP-seq data. Methods 2017;118:60–72.
13. Chakrabarti AM, Haberman N, Praznik A, Luscombe NM, Ule J. Data Science Issues in Understanding Protein–RNA Interactions. bioRxiv 2017;.
14. Dominski Z, Erkmann JA, Yang X, Sànchez R, Marzluff WF. A novel zinc finger protein is associated with U7 snRNP and interacts with the stem–loop binding protein in the histone pre-mRNP to stimulate 3'-end processing. Genes & development 2002;16(1):58–71.
15. Bailey TL, Boden M, Buske FA, Frith M, Grant CE, Clementi L, et al. MEME SUITE: tools for motif discovery and searching. Nucleic acids research 2009;37(suppl_2):W202–W208.
16. Parodi AC, Sangalli LM, Vantini S, Amati B, Secchi P, Morelli MJ. FunChIP: an R/Bioconductor package for functional classification of ChIP-seq shapes. Bioinformatics 2017;33(16):2570–2572.
17. Cremona MA, Sangalli LM, Vantini S, Dellino GI, Pelicci PG, Secchi P, et al. Peak shape clustering reveals biological insights. BMC bioinformatics 2015;16(1):349.
18. Alipanahi B, Delong A, Weirauch MT, Frey BJ. Predicting the sequence specificities of DNA-and RNA-binding proteins by deep learning. Nature biotechnology 2015;33(8):831.
19. McInnes L, Healy J, Melville J. Umap: Uniform manifold approximation and projection for dimension reduction. arXiv preprint arXiv:180203426 2018;.
20. Hartigan JA, Wong MA. Algorithm AS 136: A k-means clustering algorithm. Journal of the Royal Statistical Society Series C (Applied Statistics) 1979;28(1):100–108.
21. Green PJ, Silverman BW. Nonparametric regression and generalized linear models: a roughness penalty approach. Chapman and Hall/CRC; 1993.
22. Akaike H. Information theory and an extension of the maximum likelihood principle. In: Selected papers of hirotugu akaike Springer; 1998.p. 199–213.
23. Huppertz I, Attig J, D'Ambrogio A, Easton LE, Sibley CR, Sugimoto Y, et al. iCLIP: Protein–RNA interactions at nucleotide resolution. Methods 2014;65(3):274–287.

Click here to access/download
Supplementary Material
Supplements_1.pdf

Supplementary Material 2

Click here to access/download
Supplementary Material
Supplements_2.pdf

Supplementary Material 3

Click here to access/download
Supplementary Material
Supplements_3.pdf

Supplementary Material 4

Click here to access/download
Supplementary Material
Supplements_4.pdf

Supplementary Material 5

Click here to access/download
Supplementary Material
Supplements_5.pdf

Supplementary Material 6

Click here to access/download
Supplementary Material
Supplements_6.pdf

main.tex

Click here to access/download
Supplementary Material
main.tex
