# Supplementary material for: StoatyDive: Evaluation and classification of peak profiles for sequencing data
Source: Gigascience. 2021 Jun 18;10(6):giab045. doi: 10.1093/gigascience/giab045 (PMC8212874; doi:10.1093/gigascience/giab045)
Supplement: giab045_GIGA-D-20-00218_Revision_2 [file giab045_giga-d-20-00218_revision_2.pdf]

GigaScience

StoatyDive: Evaluation and Classification of Peak Profiles for Sequencing Data

--Manuscript Draft--

**Manuscript Number:**	GIGA-D-20-00218R2
**Full Title:**	StoatyDive: Evaluation and Classification of Peak Profiles for Sequencing Data
**Article Type:**	Technical Note
**Funding Information:**	Deutsche Forschungsgemeinschaft (322977937/GRK2344 MeInBio) — Mr. Florian Heyl<br>Deutsche Forschungsgemeinschaft (390939984 EXC-2189) — Prof. Dr. Rolf Backofen
**Abstract:**	Background<br><br>The prediction of binding sites (peak calling) is a common task in the data analysis of methods such as crosslinking immunoprecipitation in combination with high-throughput sequencing (CLIP-Seq). The predicted binding sites are often further analyzed to predict sequence motifs or structure patterns. When looking at a typical result of such a high-throughput experiments, the obtained peak profiles differ largely on a genomic level. Thus, a tool is missing that evaluates and classifies the predicted peaks based on their shapes. We hereby present StoatyDive, a tool that can be used to filter for specific peak profile shapes of sequencing data such as CLIP.<br>Findings<br><br>With StoatyDive we are able to classify peak profile shapes from CLIP-seq data of the histone stem-loop-binding protein (SLBP). We compare the results to existing tools and show that StoatyDive finds more distinct peak shape clusters for CLIP data. Furthermore, we present StoatyDive's capabilities as a quality control tool and as a filter to pick different shapes based on biological or technical questions for other CLIP data from different RNA binding proteins with different biological functions and number of RNA recognition motifs. We finally show that proteins involved in splicing, such as RBM22 and U2AF1, have potentially more sharper shaped peaks than other RNA binding proteins.<br>Conclusion<br><br>StoatyDive finally fills the demand for a peak shape clustering tool for CLIP-Seq data that fine tunes downstream analysis steps such as structure or sequence motif predictions and that acts as a quality control.
**Corresponding Author:**	Florian Heyl<br><br>GERMANY
**Corresponding Author Secondary Information:**	
**Corresponding Author's Institution:**	
**Corresponding Author's Secondary Institution:**	
**First Author:**	Florian Heyl
**First Author Secondary Information:**	
**Order of Authors:**	Florian Heyl<br>Rolf Backofen
**Order of Authors Secondary Information:**	
**Response to Reviewers:**	\section{Reviewer 1}<br>I appreciate the effort of the authors to provide additional analyses and address previous concerns, and the sections with specific edits are greatly improved. However, while the authors have clarified in many locations, there remain others (especially in

the added sections) where it remains very confusing exactly what dataset is being used for analyses or plotted in figures. I also have remaining concerns (particularly about the newly added sections), where the authors seem to assume by default that their CV metric is accurate and reliable and then use that to draw conclusions about the underlying data, but I think this assumption is something that needs to be shown by analysis in the paper and cannot be assumed.

I think the new section on replicates "Peak Profile Landscape Reveals Low Reproducibility of Binding Sites" is weak and detracts from the paper. Broadly, my major concern is that it's completely unclear whether the low reproducibility seen is a feature of the experimental eCLIP data or a technical property of their CV metric. Essentially, this section defines a new metric (the CV for each peak), sees low reproducibility in this metric across replicates, and then assumes that this must imply low reproducibility in data "However, we have to stress out that we are unsure if this is the result of the specificity of SLBP, the different quality of replicate 1 and 2, or a different source, for example, a bad immunoprecipitation." To me, this statement requires some sort of analysis (downsampling / replicate splitting?) to show what the reproducibility of their CV metric is to random sampling variability. It would also be helpful to show here real examples of peaks that the authors claim show low reproducibility across replicates, to visualize what exactly their analysis is claiming.

\begin{answer}
Thank you very much for your suggestions. We have changed the term "low reproducibility". We rephrased the title to "Peak Profile Landscape Reveals Variability of Binding Sites". We also rephrased the chapter saying: "The distribution was an indicator for some variability in the binding events. This was also striking when we compared the distribution of replicate 1 and 2. However, we have to stress out that this does not mean a poor quality of replicate 1 and 2. For a downstream analysis, for example the prediction of sequence motifs, it is worth investigating why the CV distribution of replicate 1 was very different to replicate 2. A sequence motif prediction depends on the selected binding sites, thus StoatyDive's inspection allows the user to assess the binding sites using the CV distributions. This helps to appraise if SLBP has protein domains that bind to RNA in an unspecific manner. The user can also test if the unspecific peak Figure~1b might be a false positive of PureCLIP."

To clarify, we wanted to investigate the variability of the binding events that are common for both replicates. Therefore we merged all binding sites that we have obtained from replicate 1 and 2. So we have a set of 899 peaks. We looked how these 899 peaks behaved (peak profiles shapes) for replicate 1 and 2. We took also the same 899 peaks and analyzed the profiles shapes for the control data to see if the shapes are similar to the replicates (reference point).

The chapter was designed to investigate if differences in the peak profiles occur on a set of common binding sites for the replicates. This is crucial, because in the end, the user has to know if different shapes occur for different replicates and that this variability can be inspected with our tool, which ultimately leads to a better down-stream analysis.
\end{answer}

In addition, the conclusion that their analysis indicates that the replicates are of differing quality is also a computationally testable question (for example, comparing the fold-enrichment at previously validated histone 3'UTR peaks), which would help to convince that this isn't simply reflecting low reproducibility of their metric itself. The authors do this somewhat in Sup Fig 1, but then just use that to conclude that many other RBPs are also variable across replicates; SLBP definitely seems worse than those other RBPs, but this difference should be quantified. If the authors simply mean 'there is high variability in the CV metric between replicates for some RBPs', this should be worded as such and avoid claims about reproducibility of the underlying data without further analysis.

\begin{answer}
We have removed the word reproducibility as stated in our first answer. It is true, we wanted more to clarify that there is some variability in the CV between replicates.
\end{answer}

Powered by Editorial Manager® and ProduXion Manager® from Aries Systems Corporation

I'm also unclear what the 'control' dataset used in Figure 2 (and some later analyses) is exactly - the author state that they ran PureCLIP "for each CLIP replicate separately, taking the input control into account", so what is the control peak set in Figure 2 (as I assume it can't have been PureCLIP run on the input with itself as a control)?

\begin{answer}
Please refer to the first answer for the explanation.
\end{answer}

The conclusions here are also vague and non-specific - "For a downstream analysis, for example the prediction of sequence motifs, it is worth to either exclude replicate 1 or to investigate,
why the CV distribution of replicate 1 was very different to replicate 2. Thus given the inspection of StoatyDive, the user can now decide to investigate the binding sites of replicate 1 and compare them with the input control or replicate 2." - why was it worth investigating replicates separately? I'm unclear what the authors think was improved by performing this analysis - they show that the replicates have differences in CV distribution, with replicate 1 resembling the control (as above, I don't understand what this control peak set represents), but I don't see anything other than the CV analysis to indicate that Rep1 is of worse quality than Rep2 (in peak enrichment for histone RNAs, motifs, peak enrichment above input, etc).

\begin{answer}
We rephrased the statement, which we explained in the first answer. Indeed it is better to point out that the user just observes a variability in the CV distribution between the replicates, which does not say anything about the reproducibility or that replicate 1 should be excluded from further investigations.
\end{answer}

Similarly, the authors then split the peaks by exonic and intronic and claim "It is not clear why this difference appeared, but our observations stress out how important it is to perform an experiment with replicates." - how do they stress this? I
don't see any benefit that the replicates have provided beyond the ability to say that they're different; other than Replicate 2 having more peaks, it's not described whether those additional peaks are real or false positives, and thus whether replicate 2 is better or worse.

\begin{answer}
We have rephrased this sentence slightly: "It is not clear why this difference appeared, but StoatyDive showed a variability between the different peak sets."

It was not our intention to state that replicate 2 was better than replicate 1. We thought the sentence benefits the data since it had replicates. Our results showed that some variability in the binding events might occur and highlighted that it was very good that SLBP had two replicates.
\end{answer}

I similarly think there needs to be improved clarity in the "Optimizing StoatyDive with the data of SLBP" section. I don't understand this sentence "This was achieved by the second cluster, which pointed out that the cluster had to be carefully chosen in order to remove some noise in the data (artifacts)." - does this mean that the authors removed some peaks in this set? Moreover, what are 'main cluster' and 'second cluster'? I don't see these defined anywhere.

\begin{answer}
In the section "Optimizing StoatyDive with the data of SLBP" we wrote: "We investigated the cluster with the highest (Main Cluster) and second highest (Second Cluster) number of peaks in histones." So basically we just looked at each cluster and checked how many peaks in this cluster overlap with histones. Ideally the peaks that overlap with histones should be contained in the main cluster but StoatyDive is of course not flawless and perhaps the profile shape is different for some histone peaks. That is why we investigated also the cluster (second cluster) with the second highest number of peaks overlapping with histones.

Powered by Editorial Manager® and ProduXion Manager® from Aries Systems Corporation

Main and second clusters are therefore just different subsets of the entire peak set.
\end{answer}

Similarly, "Furthermore, the same size achieved the highest TPR (0.61) and MCC (0.68) with a CV threshold of 0.2. However, the enrichment was not as good as with the clustering resulting in a mean LFC of 1.6 (median P-value = 0.108)." - I am completely lost as to what 'the same size' or 'the clustering' are referring to here in terms of datasets. This section needs to be edited to clearly state what exact set of peaks is being referred to at each point.

\begin{answer}
We rewrote the paragraph saying: "\textcolor{red}{Furthermore, the same peak length of 70 nucleotides achieved the highest TPR (0.61) and MCC (0.68) when we filtered the peaks based on a CV threshold of 0.2. However, the enrichment was not as good as with the clustering by StoatyDive, where we achieved a mean LFC of 3.4 in the second cluster that was higher than the resulting mean LFC of 1.6 (median P-value $= 0.108$) with the CV cutoff of 0.2.}

The clustering refers to the aforementioned results, where we discuss the main and second cluster. StoatyDive has generally two modes, which you see in figure 7: evaluation (CV distribution) and classification (clustering). Both can be used to filter for peak profile shapes. The table assess the power of both modes for different peak length (sizes).
\end{answer}

Figure 5 (and related text) - I don't understand what Fig. 5a and b are plotting, and perhaps more broadly what is being done here? The text seems to indicate that the authors used 10 peaks defined as broad, 10 as sharp, and 10 as plateau and considered what the other tools did, but then state that they ran StoatyDive on these (with 0.87 accuracy), so I'm confused where the initial definition of broad / sharp / plateau came from (my initial impression was that they were from the StoatyDive analysis above, but that doesn't make sense with it giving different classification here).

\begin{answer}
We explain in the revised version of the paper: "We defined peaks as sharp (CV $>$ 1.0) and broad (CV $<$ 0.05) based on the calculated CV of StoatyDive. We selected peaks shaped like plateaus by inspecting them in a genome browser." We tried not to include a bias by our findings by StoatyDive. That is why, we did not take a pre-classified set by StoatyDive, just to show that StoatyDive classifies its own clusters correctly. However, we also needed a good way to decide what a sharp and broad peak is, thus we took the CV of StoatyDive and stayed very restrictive with the choice of a broad and sharp peak. However as seen in our results, if a broad peak resembles in its shape a sharp peak it can happen that StoatyDive makes some mistakes.
\end{answer}

I'm then confused what is being displayed in Fig. 5a and b - how does Fig. 5a show a few peaks sorted incorrectly? What do the dimensions/axes mean? Similarly, for 5c and 5d, what are the colored lines versus grey lines?

\begin{answer}
Figure 5a shows the dimensionally reduced space by umap of StoatyDive. Thus Dim1 and dim2 are the features found by umap, like PC1 and PC2 for principal component analysis. It is maybe not clearly visible, since all three clusters are clearly separated with 10 peaks in each clusters. But StoatyDive indeed has two peaks in cluster 1 which should belong to cluster 2 and 2 peaks in cluster 2 which should belong to cluster 1.

Figure 5b is a different tool called SIC-ChIP. They used in their analysis pre-defined features, such as half of the width of the peak at half of the maximum $w_{h/2}$. They defined for SIC-ChIP 5 features to classify the profiles. We showed in the paper only one plot with the highest cluster separation (variance), but the others can be found in the Supplementary 5.

Regarding the colored lines we state now in the caption of figure 5: "The coloured lines show the profiles of the related cluster, whereas the grey lines correspond to all other

Powered by Editorial Manager® and ProduXion Manager® from Aries Systems Corporation

profiles."
\end{answer}

"Figure 6b shows more clearly that proteins with more than one RRM tend to have more sharper peaks." - this needs some sort of significance test

\begin{answer}
We included a significance test for this statement saying: "Figure 6b shows more clearly that proteins with more than one RRM tend to have sharper peaks (two-sided Wilcoxon test P-value ≈ 0.046)."
\end{answer}

Minor comments:

The text (particularly the new sections) would benefit from additional editing to improve text clarity - statements like "huge CV difference" should be avoided

\begin{answer}
Thank you very much for your suggestion. We looked into the text and removed or changed fuzzy adjectives and adverbs.
\end{answer}

\section{Reviewer 2}

The resubmitted manuscript by Florian Heyl and Rolf Backofen shows a good improvement. The authors addressed all the reviewer's comments in detail and restructured the manuscript accordingly. There are still a lot of interesting observations that would need further investigation but as the authors already mentioned, it would be beyond the scope of this paper. Overall, the manuscript is well written, and the method would be an interesting tool for the CLIP community. There are just few minor comments/suggestions, which are listed below:

1.Adding a library complexity distribution (number of peaks or a number of uniquely mapped reads) from each sample into the final CV distribution figures (Figure 2, Supplements1.) would be very helpful to interpret the results.

\begin{answer}
Thank you very much, indeed this would benefit the paper. We included the number of uniquely mapped reads for each sample.

The library complexity of replicate two of the SLBP data had about 0.4 times more uniquely mapped reads than replicate one. However, experiments such as TAF15 or RBM22 had almost a similar complexity, but different CV distributions. Furthermore, the number of uniquely mapped reads could not be linked to a higher or lower mean CV since experiments such as CPSF6 and HNRNPA1 (higher number of reads leads to a higher mean CV) show an opposite behavior than experiments such as TARDBP and U2AF1 (higher number of reads leads to a lower mean CV). Furthermore, StoatyDive also normalizes for the read coverage and investigates just the shape of the profile and the variance that is normalized by the mean coverage.
\end{answer}

2.Same for the Figure 3, if the authors could add a number of exonic and intronic peaks from each sample or the ratio.

\begin{answer}
We added the number of exonic and intronic peaks as well as the ratio in the caption of the picture.

We also randomly sub-sampled the exonic and intronic peaks for both replicates to 150 to test if the sample size has an influence on the difference. We got for the exonic peaks a mean CV of 0.53 for replicate 1 and 0.93 for replicate 2, and a p-value of the Wilcoxen test of < 0.05, which was quite similar to the results without sub-sampling (with mean CVs 0.48 and 0.90). The same was observable for the intronic peaks with a mean CV of 0.23 for replicate 1 and 1.11 for replicate 2 (p-value < 0.05). The results in

the paper were 0.23 and 1.26 as a mean CV for the replicates.
\end{answer}

3.Page 6: "Furthermore, the proteins HNRNPM, U2AF2, and PTBP1 have all more than 2 RRMs (3, 3, and 4, respectively), which led to the assumption that an increasing number of RRMs results in more sharper peaks. Figure 6b shows more clearly that proteins with more than one RRM tend to have more sharper peaks." - The authors assumption could be misleading without supporting literature or further analyses since there are not enough RBPs in their analysis to be confident enough. It would be better if authors could discuss this as one of the possibilities in the discussion section. There are also specific RBPs which are known to interact with RBP complexes, which could also affect the shape of the binding profile.

\begin{answer}
Thank you very much for pointing out that there is an issue with the discussion. We now state in the paper: "Furthermore, the proteins HNRNPM, U2AF2, and PTBP1 have all more than 2 RRMs (3, 3, and 4, respectively), thus it might be possible that an increasing number of RRMs results in sharper peaks. Figure~\ref{fig:proteins}b shows more clearly that proteins with more than one RRM tend to have sharper peaks (two-sided Wilcoxon test P-value ≈ 0.046). It is possible that RNA-proteins interactions become more specific with > 1 RRM and so the separation between more specific and unspecific binding sites is stricter. However, we had not taken any other RNA binding domains into account apart from RRMs and we would need to investigate more proteins to be confident about this trend."

We hope that this fits your suggestions.
\end{answer}

\section{Final remark:}

We thank all reviewers for their comments, critics, ideas and questions. It was very helpful and improved the paper a lot. You will find changes in the paper marked in red. Furthermore we changed the supplements 1. Please take a look. Thank you very much for your time and effort.

Additional Information:

Question	Response
Are you submitting this manuscript to a special series or article collection?	No
**Experimental design and statistics**<br><br>Full details of the experimental design and statistical methods used should be given in the Methods section, as detailed in our Minimum Standards Reporting Checklist. Information essential to interpreting the data presented should be made available in the figure legends.<br><br>Have you included all the information requested in your manuscript?	Yes
**Resources**	Yes

A description of all resources used, including antibodies, cell lines, animals and software tools, with enough information to allow them to be uniquely identified, should be included in the Methods section. Authors are strongly encouraged to cite Research Resource Identifiers (RRIDs) for antibodies, model organisms and tools, where possible.<br><br>Have you included the information requested as detailed in our Minimum Standards Reporting Checklist?	
**Availability of data and materials**<br><br>All datasets and code on which the conclusions of the paper rely must be either included in your submission or deposited in publicly available repositories (where available and ethically appropriate), referencing such data using a unique identifier in the references and in the “Availability of Data and Materials” section of your manuscript.<br><br>Have you have met the above requirement as detailed in our Minimum Standards Reporting Checklist?	Yes

Powered by Editorial Manager® and ProduXion Manager® from Aries Systems Corporation

Manuscript Click here to access/download;Manuscript;main.pdf

GigaScience, 2017, 1–10

doi: xx.xxxx/xxxx
Manuscript in Preparation
Technical Note

TECHNICAL NOTE

StoatyDive: Evaluation and Classification of Peak Profiles for Sequencing Data

Florian Heyl[1,*] and Rolf Backofen[1,2,*]

[1]Bioinformatics Group, Department of Computer Science, University of Freiburg, Georges-Köhler-Allee 106, 79110 Freiburg, Germany and [2]Signalling Research Centres BIOSS and CIBSS, University of Freiburg, Schaenzlestr. 18, 79104 Freiburg, Germany

*heylf@informatik.uni-freiburg.de, backofen@informatik.uni-freiburg.de

Abstract

Background The prediction of binding sites (peak-calling) is a common task in the data analysis of methods such as crosslinking immunoprecipitation in combination with high-throughput sequencing (CLIP-Seq). The predicted binding sites are often further analyzed to predict sequence motifs or structure patterns. When looking at a typical result of such high-throughput experiments, the obtained peak profiles differ largely on a genomic level. Thus, a tool is missing that evaluates and classifies the predicted peaks based on their shapes. We hereby present StoatyDive, a tool that can be used to filter for specific peak profile shapes of sequencing data such as CLIP.
Findings With StoatyDive we are able to classify peak profile shapes from CLIP-seq data of the histone stem-loop-binding protein (SLBP). We compare the results to existing tools and show that StoatyDive finds more distinct peak shape clusters for CLIP data. Furthermore, we present StoatyDive's capabilities as a quality control tool and as a filter to pick different shapes based on biological or technical questions for other CLIP data from different RNA binding proteins with different biological functions and numbers of RNA recognition motifs. We finally show that proteins involved in splicing, such as RBM22 and U2AF1, have potentially sharper-shaped peaks than other RNA binding proteins.
Conclusion StoatyDive finally fills the demand for a peak shape clustering tool for CLIP-Seq data that fine-tunes downstream analysis steps such as structure or sequence motif predictions and that acts as a quality control.

Key words: CLIP-Seq; data analysis; peak shape clustering; RNA; protein

Findings

Background

The biological function of a protein is determined by its interaction partners and the mode of interaction. Studying these interactions broadens our horizon about the cellular mechanisms such as alternative splicing and post-transcriptional regulation. Crosslinking immunoprecipitation in combination with high-throughput sequencing (CLIP-Seq) fathoms these interactions. CLIP-Seq investigates all interactions between an RNA binding protein (RBP) and its target RNAs [1]. CLIP-Seq thus scrutinizes the post-transcriptional regulation by RBPs. Prediction of binding regions (peak-calling) is a crucial step in the data analysis of methods such as CLIP-Seq. Before the peak analysis there is typically no evaluation and classification of the peak characteristics. Yet, the obtained peak set might have different peak profiles that are worth to filter to refine a downstream analysis. The different peak shapes are the result of several biological and technical problems.

Many RBPs have several binding domains with different binding affinities, and are often part of protein complexes, leading to an intricate binding pattern. As described in a review by Jankowsky, Eckhard and Harris, Michael E [2], there are specific and unspecific binders. Examples for unspecific binders are often RBPs that need to bind many RNAs such as mRNA export factors [3]. Another example of common unspecific binders are RNA helicases. However, even more specific

Compiled on: February 22, 2021.
Draft manuscript prepared by the author.

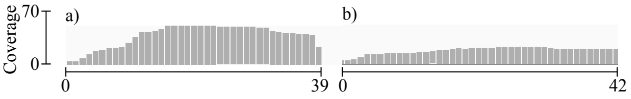

Figure 1. We show 2 significant peaks of a CLIP experiment for the protein SLBP (ENCSR483NOP, replicate 2). One can see peaks with drastically different peak profiles, pointing towards more specific (a) or unspecific (b) binding. Current analysis of CLIP binding sites is typically based on manual inspection of a few peaks. Thus a general tool is missing that allows to filter, cluster and quantify peak profiles and therefore refine downstream analysis tasks for data such as CLIP. StoatyDive assists to find and distinguish peaks like (a) and (b).

RBPs bind RNAs in large range of affinities, indicating that different binding sites vary in their binding specificity. While many factors, such as the affinity of an RBP for the binding site and the concentration of the protein and RNA, influence the binding specificity, it is likely that these factors are manifested in the CLIP binding profile landscape. At this point, however, no tool exists that can be used to study this possibility in more detail.

In addition, technical biases might change the peak profile landscape. Binding artifacts might be introduced during read library preparation. Protocol biases, for example, PAR-CLIP biases that are introduced by endonuclease and photoactivatable nucleosides [4], might also affect the binding site predictions. On top, the peak caller itself might generate specific peak profiles and false positives, which the user might not want to have in their data.

This leads to many questions in the data analysis of binding sites that can currently not be answered adequately. Examples are: Does my protein of interest bind generally specific (Figure 1a) or unspecific (Figure 1b)? Does my RBP of interest have more than one binding motif? Does my experiment have any quality issues, meaning, do my reads come from unspecific bindings because of library preparation artifacts? Does my protocol generate biases? Do I have false positives in the set of predicted peaks from my peak caller of choice?

We hereby present StoatyDive, a tool to evaluate and classify peak profiles to help to answer the aforementioned questions. StoatyDive uses the whole peak profiles as well as predefined features to do a peak shape clustering for sequencing data. In this paper, we will test StoatyDive on CLIP data of the eCLIP protocol from the histone stem-loop-binding protein (SLBP) from the study by Van Nostrand et al. [5]. SLBP has been reported to be a histone mRNA export and translation factor [6]. StoatyDive delivers several plots and a table to assess the different binding profiles of a protein. The tool assists to select specific and unspecific binding sites and to find similar shaped peak profiles. Thus, we try to refine the obtained peaks of the SLBP data to find more specific sites of SLBP. It also helps as a quality assessment to validate a CLIP-Seq or any other binding experiment. Later in the paper, we use StoatyDive to investigate the peak profile landscape of different RBPs with different biological functions and different number of RNA recognition motifs (RRM). StoatyDive comes with some test data and a quick installation guide.

Data Preparation of SLBP and Analysis

We used eCLIP data of the histone stem-loop-binding protein (SLBP; ENCSR483NOP; GSE91802; Van Nostrand et al. 5). The data comprised 2 CLIP replicates and a size-matched input control from immortalised myelogenous leukemia cells (K562). We processed the data with the snakemake pipeline SalamiSnake (https://github.com/BackofenLab/SalamiSnake, v0.0.1) for eCLIP data. SLBP has been reported to be cytoplasmic but to be present also in the nucleus [6]. Thus, we mapped the reads against the human genome (version hg38) with STAR [7] also taking the transcriptome into account. We predicted potential binding sites of SLBP with PureCLIP [8], which we ran for each CLIP replicate separately, taking the input control into account. We extended the predicted binding regions by 20 nucleotides left and right because PureCLIP often underestimates the binding region. We further fused the predicted peaks from each CLIP replicate with bedtools [9] to get a robust set of predicted binding sites, which resulted in 899 robust peaks. We executed StoatyDive (v1.1.0 with umap v0.2.5.0) with length normalization, a penalty for broader plateaus, and peak profile smoothing. The complete call was: *StoatyDive.py -a peaks.bed -b reads.bam -c hg38.chrom.sizes.txt --peak_correction --scale_max 10 --border_penalty --sm*.

Peak Profile Landscape Reveals Variability of Binding Sites

The user obtains from StoatyDive a distribution of the coefficient of variation (CV), calculated for each peak, to get a broad overview of the peak profile landscape of their experiment (see Methods). Broader peaks tend to have a CV ≈ 0. Although the CV distributions of the input control and replicate 1 of the SLBP data differed significantly (one-sided Wilcoxon test P-value = 0.03), both contained a lot of regions with a CV ≈ 0 (Figure 2, both with a mean CV of 0.47). In contrast, the CV distribution of replicate 2 was distinct (P-value < 0.05 to input control and replicate 1) since it had more peaks with a higher CV (mean CV of 1.41) and thus more specific binding events (e.g., Figure 1a, $CV \approx 5.3$). Yet, some potential binding sites were more unspecific with a CV ≈ 0 (e.g., Figure 1b, $CV \approx 0.006$).

The CV distribution of the input control was expected because an ideal control experiment should contain no real or not enriched binding events. But, the CV distribution of replicate 1 did not match the assumptions. The distribution was an indicator for some variability in the binding events. This was also striking when we compared the distribution of replicate 1 and 2. However, we have to stress out that this does not mean a poor quality of replicate 1 and 2. For a downstream analysis, for example the prediction of sequence motifs, it is worth investigating why the CV distribution of replicate 1 was very different to replicate 2. A sequence motif prediction depends on the selected binding sites, thus StoatyDive's inspection allows the user to assess the binding sites using the CV distributions. This helps to appraise if SLBP has protein domains that bind to RNA in an unspecific manner. The user can also test if the unspecific peak Figure 1b might be a false positive of PureCLIP.

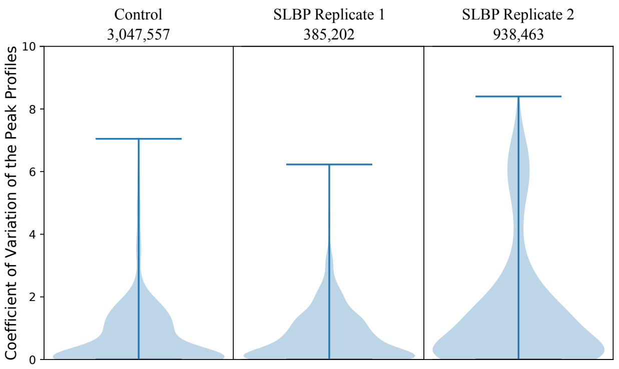

Figure 2. StoatyDive generates a CV distribution to evaluate the peak profile shapes, which can be used as a quality control. The CV distribution of the peak profiles of the input control and replicate 1 of the SLBP CLIP-Seq experiment are quite similar. In contrast, the CV distribution of replicate 2 is different. The number of uniquely mapped reads is listed below the sample name.

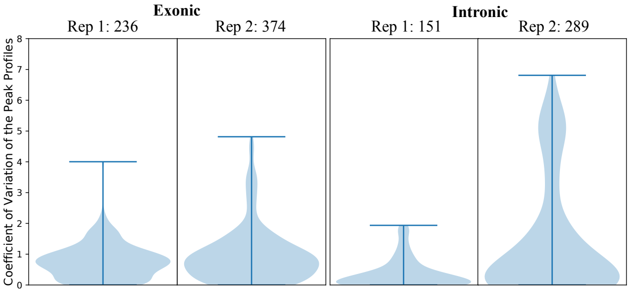

Figure 3. CV distibributions of exonic and intronic peaks reveal a difference in the profile shapes. The CV distributions of the peak profiles of replicate 1 and 2 are more similar in exonic regions than in intronic ones. The number of peaks is listed next to the sample names. The ratio of exonic and intronic peaks is about 1.6 for replicate 1 and 1.3 for replicate 2.

Checking the CV distributions of other CLIP-Seq datasets such as TAF15, TARDBP, and HNRNPA1 (Supplementary Figure 1), which we will analyze further in a later section, we saw that the CV distributions also had differences between the replicates (two-sided Wilcoxon test P-value < 0.05). However, this does not mean a low quality of the data and just highlights that it is important to do replicates in order to quantify biological and technical variance as noted in a previous CLIP study [10].

To investigate further differences between the two replicates, we split the peak set into peaks overlapping with exons and introns (see Figure 3). SLBP is a translation and transport factor, which is present in the cytoplasm as well as nucleus [6, 11]. Therefore, the replicates could have different binding events, where one replicate might have more events in cytoplasm and the other more in the nucleus. Replicate 2 had 136 exonic and 136 intronic peaks more than replicate 1. Notably, we can see a CV difference when comparing the intronic peaks of the two replicates, with a mean CV of 0.23 for replicate 1 and 1.26 for replicate 2 (one-sided Wilcoxon test P-value < 0.05). The exonic peaks on the other hand were more similar (mean CV = 0.48 and 0.90, respectively), but still the CV distributions were significantly different (one-sided Wilcoxon test P-value < 0.05). It is not clear why this difference appeared, but StoatyDive showed a variability between the different peak sets.

Seven Different Peak Shapes in the SLBP Data

For a more detailed analysis, we classified the peaks of replicate 1 and 2 with the help of StoatyDive (Figure 4). The procedure is mentioned in the methods. StoatyDive has found 7 distinguishable peak profiles for both replicate 1 and 2. We looked more closely at the profiles of replicate 2 (Figure 4b). Cluster 2 and 5, which are set apart clearly from clusters 1, 3, 4, and 6, are characterized by plateau-shaped profiles. The other groups had profiles with mountain-like shapes with peaks tending to become broader and fuzzier in the order of clusters 3, 1, 6, and 4. To return to our initial examples (Figure 1), peak profile Figure 1a was classified by StoatyDive as a small, centered mountain (Figure 4 b3), whereas peak profile Figure 1b was classified as a very broad profile (Figure 4 b4).

It is to mention that constant profiles (Figure 4) represent a constant read coverage throughout the whole peak. Because of the max-min normalization of the profile (see Methods), the value becomes 0, that is to say, the profile is not empty. In contrast, peaks shaped like plateaus have not a constant value since their coverage changes at a few positions. Furthermore, the number of clusters depend on the optimization of StoatyDive, but can also be defined by the user. Other proteins, experimental conditions, and methods might have different peak profile groups. Even in our example it is worth to investigate if cluster 4 and 6 of replicate 2 can be separated more distinctly. To do this, one could run StoatyDive again, using only the peaks of cluster 4 and 6.

The high distance between cluster 2 and 5 might have been the result of the difference between the profile borders (compare Figure 4 b2 and b5). Where cluster 2 had profiles with lots of values in the left or right side of the peak profile, cluster 5 has occupied the center of the peak profile.

A broader and fuzzier profile might not necessarily mean that it was an unspecific site. Perhaps some of them were just a collection of several specific peak profiles that were merged together. This could have happened because of the peak caller model or the peak correction and extension of StoatyDive. It is worth to take these profiles and reduce the extension, and in addition run a peak deconvolution. It is important to note that peaks shaped like plateaus might be false positives.

SLBP has been reported as an mRNA export and translation factor [6]. Thus, it is worth to investigate if peaks like Figure 1a are more informative for a translation factor than peaks like Figure 1b. That is to say, Figure 1a might be more suited for sequence and structure predictions than peak Figure 1b. Therefore, we will do a deeper inspection of group 1, 3, 4 and 6 later in the paper.

We also checked the uniqueness of the shapes by analyzing the peaks based on the reads from the size-matched input control (see Supplementary Figure 2). StoatyDive had just identified 4 different clusters, encompassing mountain-shaped peaks, as well as plateaus, and constant peaks. It was to be expected to find similar shapes in the control because the biggest challenge for peak-calling is the identification of enriched sites with different shapes between control and CLIP data [12, 13]. In a future version of StoatyDive, we will include a mode to check peak shapes between samples to see if we could improve peak-calling results with a peak shape comparison.

Information from Peak Profile Shapes

We made the assumption that replicate 1 might have more unspecific and less distinguishable profiles than replicate 2 based on the different CV distributions (Figure 2). Thus, we counted the number of peaks in each cluster for replicate 1 and 2 (Table 1). From our robust 899 peaks, in replicate 1 we had $\approx 19\%$ peaks being a sharp mountain shape (Figure 4 a4 and a6), $\approx 53\%$ being a broader mountain (Figure 4 a1, a2 and a5), $\approx 6\%$ peaks with plateaus (Figure 4 a3), and $\approx 22\%$ constant shaped peaks (Figure 4 a7). replicate 2, on the other hand, had $\approx 29\%$ sharply mountain-shaped peak profiles (see Figure 4 b1 and b3), so 94 more than replicate 1. This corroborated the assumption that replicate 1 had broader and more unspecific sites. Thus, replicate 2 had only $\approx 49\%$ broad peak profiles (Figure 4 b4 and b6), and only 2 constant peak profiles (Figure 4 b7). Yet, replicate 2 had $\approx 21\%$ peaks with plateaus (Figure 4 b2 and b5).

We further investigated the biological function of different peak profiles of replicate 2. Since SLBP targets histone mRNAs [6], we intersected known annotated mRNAs of histones with the peaks of the different profile clusters (Table 1). From the 899 peaks, only $\approx 13\%$ of replicate 2 overlapped with mRNAs of histones. Yet, of these 118 peaks almost all came from group 1, 3, 4, and 6. These groups were either spiky, or broader mountain-shaped peak profiles. For example, we found a sharper peak located on RNU7-1 RNA (U7 small nuclear 1) that contains a stem loop that might be potentially targeted by SLBP [14]. The peak got a CV of 3.9 and was classified into the peak profile cluster 3 of replicate 2. Only 5 peaks intersected with histone mRNAs that had a profile shaped like a plateau (Figure 4 b5). This endorsed the assumption that peak

Table 1. Number of peaks of SLBP for different shape groups.

Replicate	Total	Sharp	Broad	Plateau	Constant
1	899	171	481	51	196
2	899	265	444	188	2
	Peak Summits in Histone mRNAs				
1	116	22	86	6	2
2	118	42	71	5	0

profiles shaped like plateaus were less informative. The observation also suggests that broader profiles were still informative because some of them overlapped with histone mRNA.

Next, we used MEME-ChIP [15] to search for sequence motifs associated with the different peak shape groups of the second replicate of SLBP. We have found 2 significantly enriched motifs associated with the plateau peaks and 3 motifs associated with the sharper peaks (Table 2). Yet, both the plateaus and the sharper peaks had two similar sequences motifs. Both motifs *(G)GCUCUUU(U)* and *(CA)GAGCCA(C)* were higher enriched in the sharper-shaped peaks. On the other hand, we found > 10 enriched motifs for the broader-shaped peaks. The motifs of that peak set were very different to the motifs of the plateaus and sharper-shaped peaks. Even the first 3 significantly enriched motifs had more noise and consequently were less enriched than the motifs of the other 2 peak shape groups. The E-values of all motifs were also > 1000 times higher for broader peaks than for sharper peaks. Peaks shaped like plateaus were slightly more significant than broader peaks.

On further inspection of those peak motifs in histone mRNAs, we found that the 3 motifs of the sharper shape peaks covered 10 more histone mRNAs (49 in total) than the broader-shaped peaks (39 in total). This endorsed the observation that broader peaks encompassed more noise. For example, the second motif of the broad peaks *CA(A/C)CAAG* came close to the third sequence motif, with the sequence *A(C/A)CCAAAG*, of the sharper shape peak group. The second motif had the highest occurrence in histone mRNAs for the broader peaks. Thus, the broader peak set might include true binding sites but with some higher additional noise. Furthermore, we already showed that the plateau group might also hold some peaks that are true binding sites (Table 1), which was confirmed by the similar sequence motifs to the sharper peak set. We could confirm that the 2 sequence motifs that are present in plateaus were also present in histone mRNAs (Table 1). All in all, just the sequence motifs analysis showed how different the outcome of subsequent tasks can be for different peak shape groups. Yet, we cannot confirm the biological truth behind those motifs as it requires further experiments to verify them.

Optimizing StoatyDive with the data of SLBP

We investigated the peaks of the second replicate of SLBP further and took the CLIPper peaks (ENCFF127WAK) from the study by Van Nostrand et al. [5]. We wanted to check the specificity and sensitivity of StoatyDive for different CV cutoffs and peak sizes (see Supplementary Table 3). PureCLIP does not give a FC or P-value, so it was only possible to calculate those features with the CLIPper peaks. Since SLBP binds mainly histone mRNAs [6], we defined peaks in histones with a $\log_2$ fold change (LFC) >= 1 and a P-value < 0.05 as true positives. A true negative was a non-significant peak in a region that did

Table 2. First 3 MEME-ChIP motifs for the different peak shape groups of SLBP with the E-value, the portion of sequences that have the motif, and the number of peaks that have the motif and which also intersect with histone mRNAs.

Shape	Motif 1	Motif 2	Motif 3
Broad	3.5e-4; 5.85%<br>13<br>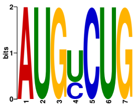	1.5e-3; 5.40%<br>18<br>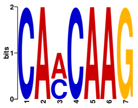	1.5e-3; 5.40%<br>8<br>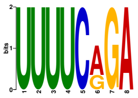
Sharp	5.7e-12; 20.00%<br>23<br>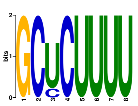	8.6e-9; 18.87%<br>17<br>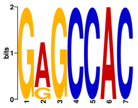	1.0e-3; 9.06%<br>9<br>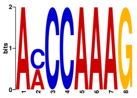
Plateau	1.6e-5; 15.42%<br>3<br>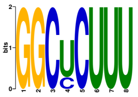	1.6e-3; 12.23%<br>2<br>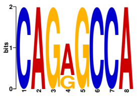	

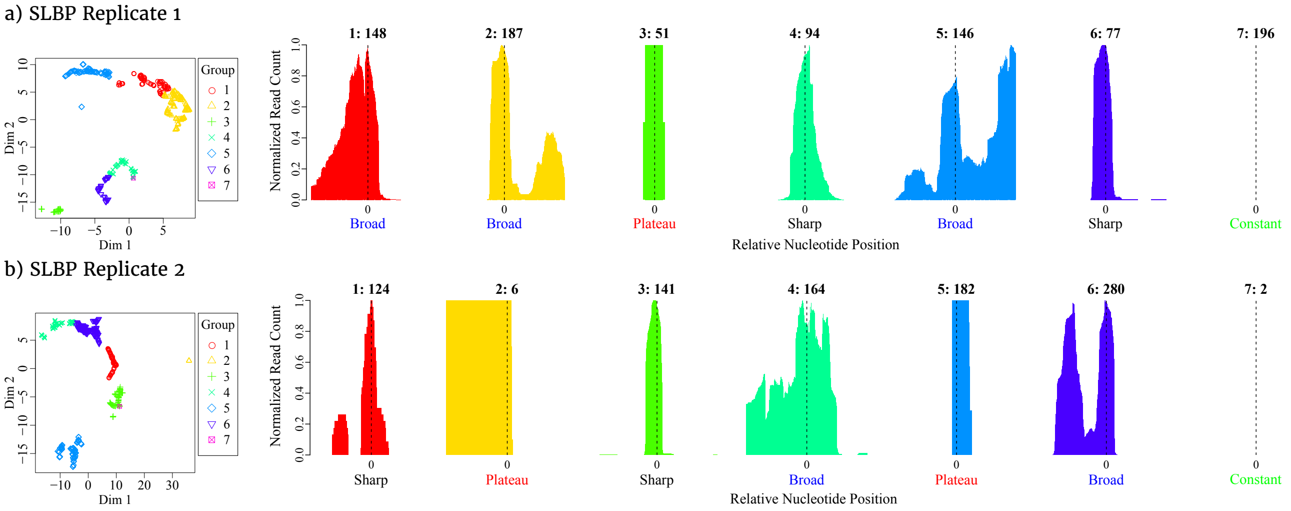

Figure 4. Results of the peak profile clustering with StoatyDive (procedure described in the methods). We applied StoatyDive to the SLBP data [5]. StoatyDive has found 7 different peak profile shapes in the data of replicate 1 (a1–7) and replicate 2 (b1–7) of SLBP. We present one example profile for each cluster with the number of peaks on top. For replicate 1 and 2 we could separate between very thin and specific mountains such as Figure 1a and very broad profiles like Figure 1b. We also found peaks shaped like plateaus, such as 3a, and constant peaks, for example, 7a. The profiles also vary slightly between groups. For example, 6b has more than one spiky mountain in contrast to 1b.

not overlap with a histone. We investigated only peaks where we could calculate a CV and used StoatyDive with a peak size of 30 (median), 40 (Q3), 70 (Q3 + 1.5×IQR) and a maximum peak length of 201 nucleotides. The peak sizes were chosen based on the length of all peaks (see Supplementary Figure 4). The Matthews correlation coefficient (MCC) is more informative in case of imbalanced datasets, which was the case of the SLBP data. The true negative (TNR) was always at 1.0 because the peak set had no peaks in histones that were not significant (no false positives). We investigated the cluster with the highest (Main Cluster) and second highest (Second Cluster) number of peaks in histones. Looking solely at the clustering, we achieved the highest true positive rate (TPR) (0.69) and MCC (0.66) with a peak length of 70 than does the set of all CLIPper peaks with a TPR of 0.48 and MCC of 0.57. This was achieved by the second cluster, which pointed out that the cluster had to be carefully chosen in order to remove some noise in the data (artifacts). The cluster had also the highest enrichment with a mean LFC of 3.4 (median P-value < 0.05) than does all CLIPper peaks with a value of 1.9 (median P-value = 0.038). Furthermore, the same peak length of 70 nucleotides achieved the highest TPR (0.61) and MCC (0.68) when we filtered the peaks based on a CV threshold of 0.2. However, the enrichment was not as good as with the clustering by StoatyDive, where we achieved a mean LFC of 3.4 in the second cluster that was higher than the resulting mean LFC of 1.6 (median P-value = 0.108) with the CV cutoff of 0.2. Based on these different sets, we observed that the CV was sometimes lower in the main or second cluster. Together with the previous results, we concluded that broad and sharp peaks equally play an important role for SLBP.

Comparison to Existing Tools

To further validate StoatyDive, we applied 2 other peak shape clustering tools, namely FunChIP (Parodi et al. 16; version 0.99.4) and SIC-ChIP (Cremona et al. 17; current release), to the second replicate of SLBP. To have a better ground truth, we took 10 peaks of three different peak shape groups (broad, sharp, and plateau) to define a test set with three distinct peak shapes from real CLIP-Seq data (in total 30 peaks). We defined peaks as sharp (CV > 1.0) and broad (CV < 0.05) based on the calculated CV of StoatyDive. We selected peaks shaped like plateaus by inspecting them in a genome browser. We strictly used the output of the tested tools. Both tools were designed and tested for ChIP data. A peak shape clustering was so far not done for CLIP data and a specific tool for that data type did not exist to the best of our knowledge. We applied SIC-ChIP with $N = 10$ and $toll = 10$ (the default parameter set resulted in errors) and ran FunChIP according to the manual in Bioconductor with the smoothing parameter $lambda = 10^3$. StoatyDive classified most peaks correctly into the 3 peak shape groups (Figure 5a, accuracy (ACC) = 0.87). It sorted 4 peaks incorrectly that came from the broad and sharp peak group. Sharp and broad peaks are harder to cluster and a second factor such as the CV helps to give a final assessment over the shape of the peak. SIC-ChIP identified up to 6 different peak shapes (Figure 5b), whereas FunChIP found 3 (Figure 5c and d, ACC = 0.6). Furthermore, SIC-ChIP as well as FunChIP had clusters that are mixed and not as well separated as with StoatyDive. SIC-ChIP's predefined shape indices were not enough to separate the peak shape profiles properly, as shown for one scatter plot (Figure 5b) with the highest explained variance (cluster separation). In turn, FunChIP performed slightly better, finding profiles with different summit intensities. However, for the smoothed (Figure 5c) as well as the smoothed and scaled profiles (Figure 5d) the clusters included a lot of profiles with different shapes. For example, cluster 2 and cluster 3 of the smoothed profiles seemed very similar. Thus, FunChIP's approach to use the whole profile without any predefined features or dimensional reduction was also not enough to separate the peak shapes in the same way as with StoatyDive.

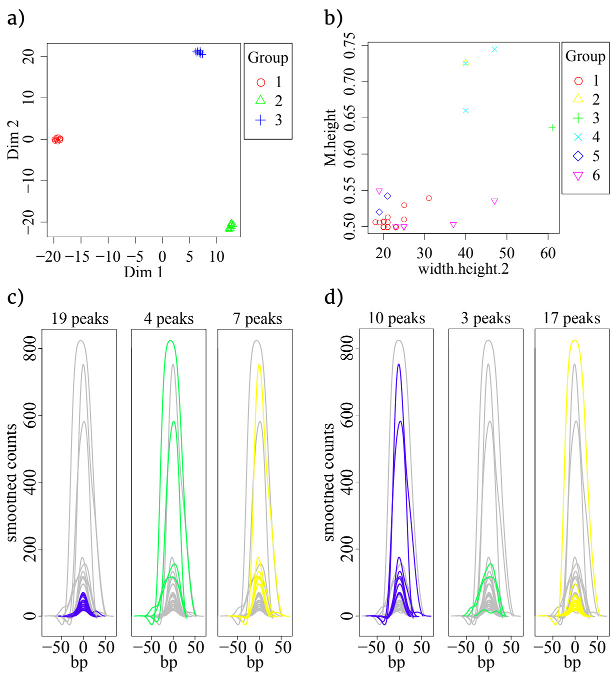

Figure 5. Peak shape clustering with StoatyDive, SIC-ChIP [17], and FunChIP [16] on a set of selected peaks from 3 different peak shapes of the second replicate of SLBP. (a) StoatyDive successfully identified the 3 distinct peak shape groups with 10 peaks each, but a few peaks were sorted incorrectly (ACC = 0.87). (b) From the 5 shape indices of SIC-ChIP (see Supplementary Figure 5), we picked one scatter plot (b) with the highest explained variance ($w_{h/2}$ vs $\frac{M}{h}$) to show the clustering of SIC-ChIP. FunChIP was only able to identify 3 distinct clusters (ACC = 0.6). The (c) smoothed and (d) smoothed and scaled profiles were clustered mostly on the intensity of the summit. The coloured lines show the profiles of the related cluster, whereas the grey lines correspond to all other profiles.

Investigation of eCLIP Protein Profiles

We further investigated the peak shapes of several proteins from the study of Van Nostrand et al. [5], namely: CPSF6, CSTF2T, EWSR1, LARP7, RBM22, SAFB2, SLBP, SLTM, TAF15, TRA2A, U2AF1, HNRNPA1, IGF2BP1, IGF2BP2, NONO, SRSF1, TARDBP, HNRNPM, U2AF2, and PTBP1. We took the robust peaks (peak-calling, IDR, signal normalization) and the bam files, which were used for the peak-calling, from each protein from the ENCODE database. We chose the data from the eCLIP experiment on K562 and focused on proteins where the biological and molecular function and the number of RRMs are clearly listed on UniProt. Thus, we wanted to investigate if the number of RRMs or the function of the protein by any means affected the shape of the peak profiles and consequently led to more or less broader peaks. We therefore took the files from ENCODE and merged both bam files (replicates) for the coverage. We then used StoatyDive with *--peak_correction --scale_max 10 --border_penalty --sm --peak_length* 77 *-k* 3. All peaks were therefore extended or shrunk to a length of 77 nucleotides. This was based on the observation that the third quartile of all peaks from all proteins was 77 nucleotides long (see Supplementary Figure 4). In addition, StoatyDive achieved for the SLBP data a better TPR and MCC with a peak size of 70 and a CV thresh-

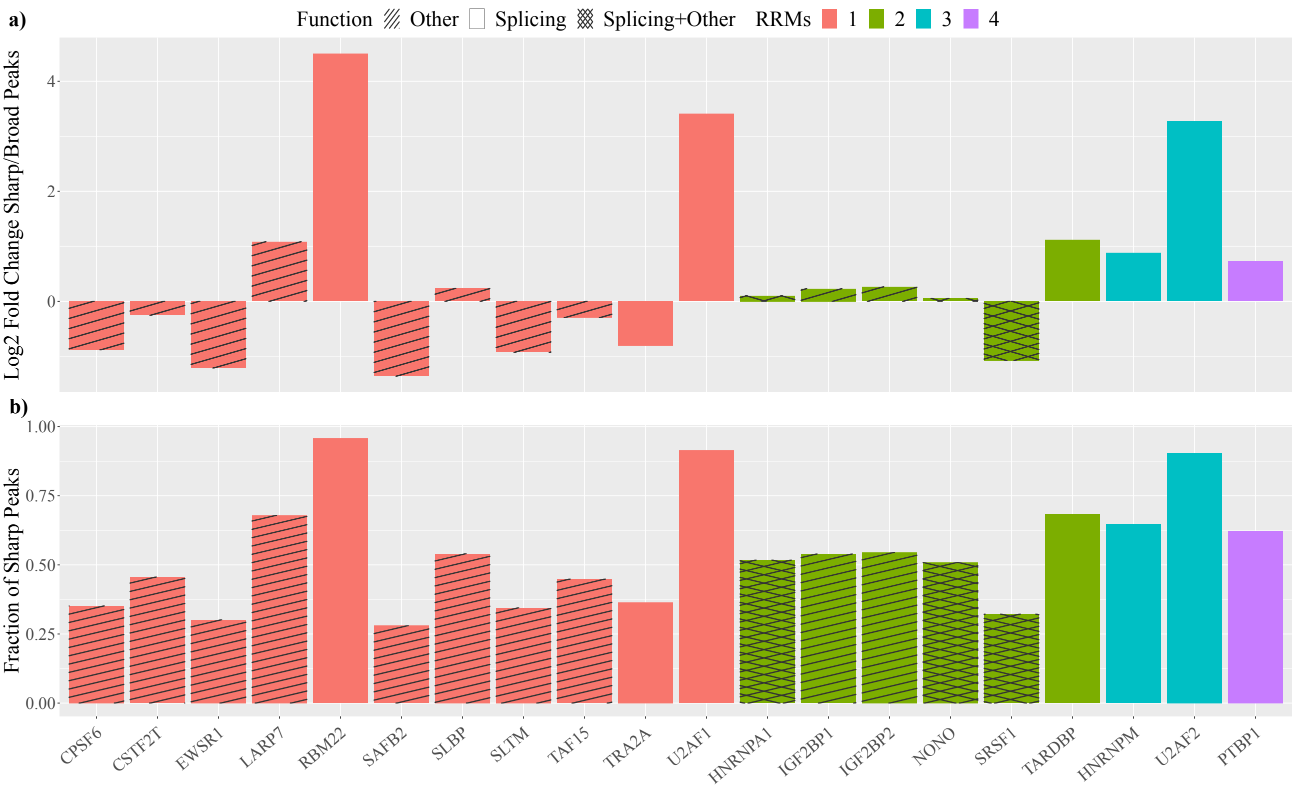

Figure 6. (a) Log_2 fold change of the number of sharp peaks versus the number of broad peaks; (b) fraction of sharp peaks. UniProt lists the proteins CPSF6, CSTF2T, EWSR1, LARP7, RBM22, SAFB2, SLBP, SLTM, TAF15, TRA2A, and U2AF1 with one RRM and the proteins HNRNPA1, IGF2BP1, IGF2BP2, NONO, SRSF1, TARDBP, HNRNPM, U2AF2, and PTBP1 with at least two RRMs. Proteins with more than one RRM tend to have sharper-shaped peaks, but also equally have broader peaks. The proteins RBM22, U2AF1, TARDBP, HNRNPM, U2AF2, and PTBP1 had a higher number of sharper peaks and all of them are involved in RNA splicing (colored bars). Other proteins with different functions (stripes) often have less sharper peaks.

old of 0.2 (see Supplementary Figure 3). The results of SLBP showed that it may be wise to combine the clustering and the CV threshold to assess the profile landscape of other proteins. We therefore defined a peak as sharp if it had a CV > 0.2 and it fell into a cluster that was generally sharper. A cluster was declared as sharp if the median CV of the cluster was bigger than the median CV of the whole peak set. All other peaks were classified as broad.

The proteins LARP7, RBM22, SLBP, U2AF1, IGF2BP2, NONO, TARDBP, HNRNPM, U2AF2, and PTBP1 had a higher number of sharper-shaped peaks, whereas the rest of the proteins had a higher number of broader-shaped peaks relative to the other shape (Figure 6a). Protein IGF2BP1 was almost half sharp and half broad peaks. UniProt lists the proteins CPSF6, CSTF2T, EWSR1, LARP7, RBM22, SAFB2, SLBP, SLTM, TAF15, TRA2A, U2AF1 with one RRM and the proteins HNRNPA1, IGF2BP1, IGF2BP2, NONO, SRSF1, TARDBP, HNRNPM, U2AF2, PTBP1 with at least two RRMs.

We could observe a trend between the number of RRMs and the number of sharper-shaped peaks (Figure 6a). From 11 proteins with 1 RRM just 4 had sharper peaks than broader-shaped peaks and 8 out of 9 proteins with at least 2 RRMs had sharper-shaped peaks. Furthermore, the proteins HNRNPM, U2AF2, and PTBP1 have all more than 2 RRMs (3, 3, and 4, respectively), thus it might be possible that an increasing number of RRMs results in sharper peaks. Figure 6b shows more clearly that proteins with more than one RRM tend to have sharper peaks (two-sided Wilcoxon test P-value ≈ 0.046). It is possible that RNA-proteins interactions become more specific with > 1 RRM and so the separation between more specific and unspecific binding sites is stricter. However, we had not taken any other RNA binding domains into account apart from RRMs and we would need to investigate more proteins to be confident about this trend.

Another observation was that shapes of splicing factors (RBM22, U2AF1, TARDBP, HNRNPM, U2AF2, and PTBP1) tend to be sharper than broader (Figure 6a). Yet, proteins, such as SRSF1, had broader peaks and are also involved in splicing. On the other hand, proteins not involved in splicing such as EWSR1, SAFB2, SLTM, and TAF15 clearly showed more broader-shaped peaks. The proteins SLBP, HNRNPA1, IGF2BP1, IGF2BP2, and NONO almost had an equal number of sharper or broader-shaped peaks, and all these proteins have multiple functions, contributing to at least two biological processes, such as transport and translation in the case of SLBP [6]. SRSF1 is also a multi-functional protein. Perhaps, that is the reason why it has broader peaks even though it is a splicing factor. We also have to consider a combination of both factors, the number of RRMs and the biological function, which is a question for the future because also technical factors might play a role for peak shapes. For example, the number of broader and sharper-shaped peaks of the protein SLBP was different from the previous results (Table 1). This can be the effect of a different peak caller (the study of Van Nostrand et al. [5] used CLIPper) or the different data processing steps.

We also checked whether our result that RBPs involved in splicing have sharper peaks can have technical reasons. In this case, a splicing related protein could have more peaks that are split over two exons, which are detected by the peak caller as two separate but sharp peaks (split peak). So we investigated the number of peaks that fall into introns (90% overlap) for the proteins that are involved in the splicing process. We used bedtools set to a strict overlap (intersect -u -s -f 0.9) to investigate potential split peaks. The proteins PTBP1 ($\approx$ 85%),

RBM22 ($\approx$ 62%), TARDBP ($\approx$ 79%), and HNRNPM ($\approx$ 87%) had more than 50% of peaks in introns, which deflected the assumption of split peaks. However, the proteins U2AF1 ($\approx$ 17%), and U2AF2 ($\approx$ 15%) had more peaks in exon regions, where the possibility of split peaks might still occur. It is important to note that this does not mean that the aforementioned proteins bind generally more introns or exons. As there exist no tool that can correct for split peaks, a further analysis for these proteins was required. We checked for potential split peaks by extending the peaks that fall completely into exons by 5 nucleotides to each side. Next, we intersected those extended peaks with introns to see if they are close to the exon boundaries. We found for U2AF1 only 27 peaks (0.72%) and for U2AF2 5 peaks (0.40%) that are potential split peaks, again deflecting the assumption of a technical artifact in the peak set of splicing factors. Thus, the sharpness of the peaks of U2AF1 and U2AF2 was potentially not the result of the peak-calling.

Potential Implications

StoatyDive is a powerful tool that can evaluate and classify peak profiles. It can be used in any sequencing data analysis that involves the prediction of binding sites such as CLIP-Seq, or ChIP-Seq. Within this work, we provided an example for SLBP to show the usability of StoatyDive. First, it is possible to assess the quality of an experiment such as CLIP. The CV is just one quality factor and we recommend to test other features as well, such as the read coverage correlation. Second, StoatyDive assists to evaluate the binding specificity of the protein. The normalized CV distribution produced by StoatyDive provides valuable information for the user. A protein that binds very specific will have a distribution concentrated around a normalized CV of one. A protein with a lot of unspecific bindings will have a normalized CV distribution ≈ 0. Third, StoatyDive helps to filter for specific and unspecific binding sites to investigate if the protein has multiple protein domains that have different binding mechanisms. A finer distinction can be made with the classification mode of StoatyDive. This helps to identify peak profiles with a specific shape and filter them based on the corresponding biological question and function of the protein. For example, a transcription factor might have more specific bindings (more spiky mountains), than a protein complex or a helicase (more broader mountains). Fourth, the results of StoatyDive can be used to validate a peak caller (e.g., PureCLIP), that is to say, one can assess how many false positives are in the peak sets based on the shape. Different peak caller might result in disparate peak sets and consequently different peak profile shapes.

StoatyDive is a very powerful, well documented, and easy to apply tool that refines the binding site detection in the data analysis such as CLIP-Seq. Nevertheless, StoatyDive is a very general tool. In the future it is worth to investigate, if StoatyDive can be used with different types of peak-calling outputs and data types of sequencing data (e.g., ChIP-Seq, ATAC-Seq, Ribo-Seq, and others). It serves as a quality control and filtering step to select specific binding profiles, which therefore allows to improve other binding site prediction tools such as DeepBind [18], or any other subsequent analysis tasks, to increase the accuracy for the prediction.

Methods

Peak Correction, Extension and Coverage Calculation

StoatyDive was implemented in python (>= 3.6) and R (>= 3.4.4). The tool needs three files: the predicted binding regions of a peak-calling algorithm in bed6 format, a bam or bed file that was used for the peak-calling (experiment or control), and a tabular file of the chromosome size of the reference genome (Figure 7).

First, StoatyDive checks if a peak profile needs to be centered (peak correction). In the default mode, the profiles are centered by a convolution with a standard normal distribution. The maximum value of the convolution gives the nucleotide shift of the peak profile to center the peaks. So the window with the peak length is shifted to the center of the peak (Figure 7 step 1). With this approach we retain the context and take care of two problems. First, peak callers often produce peaks that are not correctly centered. Second, dimensionality reduction methods, such as uniform manifold approximation and projection for dimension reduction (uMAP; McInnes et al. 19), are not translation invariant. Thus, two profiles with the same shape but in a different relative genomic position might end up in different locations in the new dimensional space.

After the peak correction, StoatyDive extents the peaks by default to the maximal peak length of the given peak set (Figure 7 step 2). This removes the peak length as a potential feature for the evaluation and classification. StoatyDive then calculates the read coverage (Figure 7 step 3) for each position inside a peak with the help of bedtools [9].

Evaluation of Peak Profiles

With the results of bedtools, StoatyDive evaluates every peak i from the total set of k peaks. StoatyDive will estimate the read count for every peak as a negative binomial $X_i \sim \mathrm{NB}(r_i, p_i)$ with the hyperparameters r_i (number of hits) and p_i (probability of a hit). It then calculates the coefficient of variation (CV) for every peak. A simple estimation of the variance is not enough because the profile depends on the read coverage. Thus, to be able to compare each peak profile we have to normalize for the expected number of reads to adjust the variance. So the CV for each peak,

$$CV_i = \sqrt{\frac{1 - p_i}{r_i}}, \quad (1)$$

is calculated with the estimated hyperparameters. In the last step, StoatyDive normalizes the CV score by the max and min of all scores,

$$CV'_i = \frac{CV_i - \min(\forall CV)}{\max(\forall CV) - \min(\forall CV)}. \quad (2)$$

At the end, our defined CV score will range from $CV_i = [0, \infty]$ and the normalized score from $CV'_i = [0, 1]$, with a $CV'_i = 0$ for a more unspecific binding and $CV'_i = 1$ for a more specific one.

Classification of Peak Profiles

StoatyDive classifies the peak profiles in an unsupervised manner using uMAP [19] and k-means clustering [20]. Yet before clustering, StoatyDive processes the peak profiles. First, the profiles are normalized based on the individual maximum and minimum read count, since we are only interested in the shape of the profiles and not in the absolute read counts (Figure 7 step 4). So assuming each peak X_i has $x_1, x_2, x_j ..., x_n$ nucleotides, we normalized the peaks by $x_j = \frac{x_j - \min(X_i)}{\max(X_i) - \min(X_i)}$. Second, the peak profiles are smoothed (Figure 7 step 4) with a spline regression [21]. The step reduces the noise for each profile and distributes the data more uniformly on the current manifold. The latter is important since it is the data assumption of uMAP. StoatyDive

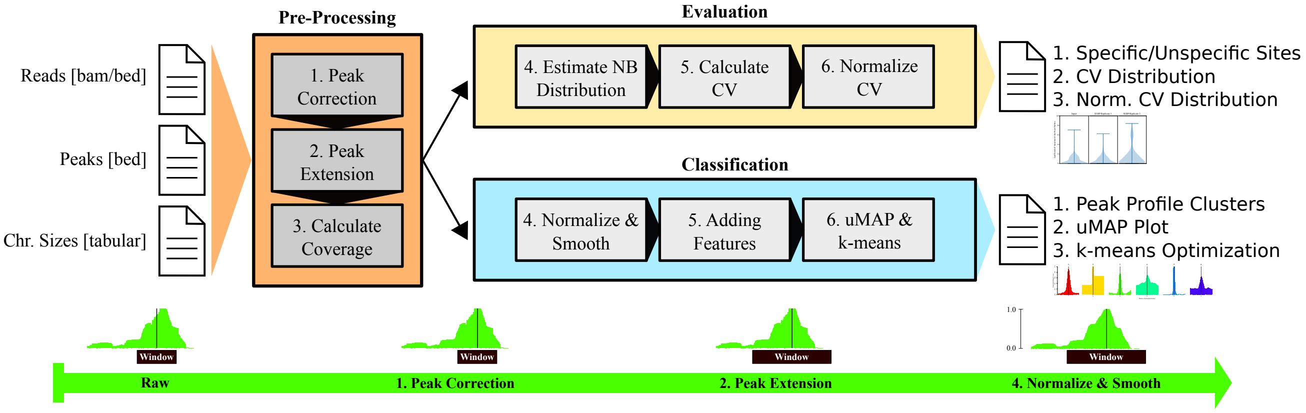

Figure 7. Overview of the StoatyDive pipeline. It consist of 2 major modules, namely the evaluation and the classification of peak profiles. The user has to provide reads (or events), peaks and a chromosome size file. StoatyDive then shifts the peaks to their correct center (peak correction), extents the peaks to a common length (maximal peak length of peak set or user defined value), and calculates the coverage with bedtools [9]. The peak correction can be turned off. In the evaluation, StoatyDive then estimates the read coverage as a negative binomial. From the hyperparameters it calculates the coefficient of variation (CV) and normalizes it (Equations 1 and 2). The normalized CV can then be used to divide the peaks into specific and unspecific sites. Furthermore, the CV distribution acts as a quality control between control and signal experiments. In the classification, StoatyDive first normalizes the peak profiles to remove the intensity as a feature. Then it smooths the profiles to support the data assumptions of uMAP [19] and to remove some noise. After that, it adds curve specific features to the data. The higher dimension of the data is then reduced with uMAP. StoatyDive then clusters the new data with k-means [20]. The user then obtains several plots and a table to investigate the different peak profile clusters.

further adds curve specific features to the processed peak profiles including: the number of maximal values, the area under the curve, and the arc length. StoatyDive applies uMAP to the final data with 5,000 epochs, 2 components (dim = 2), a minimum distance of 0.01 and a size of the local neighborhood of 5. The original and high dimensional profiles often suffer from the curse of dimensionality which would lead to a higher number of individual clusters. The dimensional reduction was optimized with some test data comprising four different sets of distributions: a uniform distribution, a linear distribution, an unimodal Gaussian distribution, and a bimodal Gaussian distribution. Subsequently, StoatyDive applies k-means clustering to the new data with 100 initializations, and maximal 10,000 iterations. The number of clusters k is found by convergence of the total within-cluster sum of squares and checked with the Akaike information criterion (AIC; Akaike 22). We also tested other dimensionality reduction methods (see Supplementary Figure 6) such as principal component analysis (PCA), a self-organizing map (SOM), and t-Distributed Stochastic Neighbor Embedding (t-SNE). However, none of them came close to the results of uMAP.

Output of StoatyDive

For the peak evaluation, StoatyDive generates a plot of the CV (Equation 1) and normalized CV (Equation 2) distribution (Figure 7). The user receives a first impression of the binding specificity of the protein of interest from the CV distribution. An unspecific binder has a CV distribution ≈ 0. A more specific binder has a CV distribution ≥ 1. The CV distribution can also be used as a quality control to compare control and signal experiments. A quality breach might have occurred if the distributions of the control and signal experiment almost look identical. A control experiment should normally have a CV distribution ≈ 0, with only a very few binding sites showing higher CVs.

The normalized CV distribution helps to evaluate the peaks based on the individual experiments. An empirical threshold is set at a CV of 0.2 (Equation 1), below which binding sites are deemed unspecific. The user can change the threshold. Keep in mind, the threshold for the normalized CV is relative in accordance to the individual experiment.

For the peak classification, StoatyDive generates a plot of the k-means optimization and a plot of the dimensional reduction with uMAP, which can be used to readjust the number of k clusters if this is necessary. The user also receives a set of example peak profiles and smoothed peak profiles of each cluster, which can be used to investigate the identified shapes. For a general trend, StoatyDive delivers average profiles for each cluster.

The final output of StoatyDive is a CV sorted table of the whole peak set, from the highest to the lowest CV. Each peak is labeled with 0, for more specific binding sites, and 1, for more unspecific sites. The table also lists for each peak the cluster number (group number) of the peak profile shape.

Important Options of StoatyDive

The peak correction (Figure 7 step 1) can be turned off. The user can also change the translocation scheme of the peak profiles to shift them based on the maximal value (summit). The maximum translocation scheme is useful for nucleotide specific events such as truncation events in the case of iCLIP data [23]. StoatyDive has also the option for a different CV score that penalizes peaks within broad plateaus. StoatyDive then adjusts the CV score of peaks that are covering a small appendage of a read stack. Furthermore, the user can provide a maximal score to StoatyDive to normalize the CV distribution (Equation 2). This option helps to compare the CV distribution between experiments in accordance to their disparate peak sizes and total amount of reads. StoatyDive also has a threshold for the normalized CV score to divide the peaks into more specific and more unspecific binding sites, which the user can change.

StoatyDive has two major parameters for the peak profile classification (Figure 7 step 6). First, the user can adjust the maximal amount of potential peak clusters identified by the k-means clustering. Yet, the final number of peak clusters will be optimized by StoatyDive. The parameter is an upper bound. However, the user has the option to force StoatyDive to use k specific clusters. The smoothing (Figure 7 step 4) of the peak profiles can also be adjusted by the user. The default was optimized with different test sets. Increasing the parameter (> default) might underfit the smoothing and thus lead to fewer peak clusters. A lower value (< default) might overfit and so lead to more clusters. The smoothing can also be turned off,

but it is recommended to turn it on.

Availability of Supporting Source Code and Requirements

Project name: StoatyDive
Project home page: https://github.com/BackofenLab/StoatyDive
Conda: https://anaconda.org/bioconda/stoatydive
Operating system(s): Unix
biotools:StoatyDive
RRID:SCR_018796

Availability of Supporting Data and Materials

StoatyDive provides a small dataset for a test run, which can be found in the github repository. The whole eCLIP data used in this paper, such as SLBP or RBFOX2, is listed in the supplementary of the study by Van Nostrand et al. [5].

Additional files

Supplementary Figure 1. CV distributions of all other proteins analyzed for figure 6 with two-sided Wilcoxon test P-value, the number of uniquely mapped reads and the mean CV for each replicate. The two replicates quite often have different CV distributions.

Supplementary Figure 2. We applied StoatyDive to the size matched input control of the SLBP data [5]. StoatyDive has found 4 different peak profile shapes, broad (cluster 1), plateau (cluster 2), sharp (cluster 3), and constant (cluster 4). The supplements also include the average profiles for replicate 1 and 2 to show the overall trend of the clusters.

Supplementary Table 3. Mean CV, variance of the CV, mean $\log_2$ fold change (LFC) enrichment between the control and CLIP experiment, median P-value, true positive rate (TPR), true negative rate (TNR), accuracy (ACC), and Matthews correlation coefficient (MCC) for the analyzed peaks (All Peaks) of replicate 2 (ENCFF127WAK) from the study by Van Nostrand et al. [5]. Features are listed for the peak shape cluster with the highest number of peaks in histones (Main Cluster) and second highest number (Second Cluster), and for the peaks with a CV smaller or bigger a threshold of 0.2, 0.5, and 0.8, using different peak lengths (30, 40, 70, and maximum peak length of 201 nucleotides). We achieved the best TPR, ACC and MCC with a peak length of 70 and with a CV cutoff of 0.2.

Supplementary Figure 4. Peak lengths of the peak set (ENCFF127WAK) for the second replicate of SLBP and peak lengths of all other proteins of the eCLIP data from the study by Van Nostrand et al. [5].

Supplementary Figure 5. All scatter plots from SIC-ChIP [17] for the artificial SLBP data.

Supplementary Figure 6. We tested different dimensional reduction methods such as PCA, SOM, and t-SNE on the CLIP data of SLBP. The PCA has no clear clusters for replicate 2, which is similar for t-SNE on replicate 1 and 2. Using an optimized SOM delivers a feature layer with a very high activated hidden unit for replicate 2. It is hard to see any distinct clusters from the counts (activation) of each hidden unit. uMAP can clearly separate the data into more defined clusters. Furthermore, it is much easier to interpret the results of uMAP, whereas an artificial neural network, such as a SOM, generates a feature layer (hidden layer) that is hard to explain.

Declarations

List of abbreviations

ACC: Accuracy; CLIP-Seq: Crosslinking immunoprecipitation in combination with high-throughput sequencing; CV: Coefficient of variation; LFC: $\log_2$ Fold Change; MCC: Matthews correlation coefficient; PCA: Principal component analysis; RBP: RNA-binding proteins; RRM: RNA recognition motifs; SOM: Self-organizing map; TPR: True positive rate; TNR: True negative rate; t-SNE: t-Distributed Stochastic Neighbor Embedding.

Ethical Approval

Not applicable

Consent for Publication

Not applicable

Competing Interests

The authors declare that they have no competing interests.

Funding

This study was funded by the Deutsche Forschungsgemeinschaft (DFG, German Research Foundation) grant 322977937/GRK2344 2017 MeInBio – BioInMe Research Training Group, and Germany's Excellence Strategy (CIBSS – EXC-2189 – Project ID 390939984).

Author's Contributions

F.H. performed the computational analysis and tool developement. R.B. initialized the project, and supervised the research. F.H. and R.B. wrote the manuscript. All authors read and approved the final manuscript.

Acknowledgements

We are grateful to Gokcen Eraslan for his support.

References

1. Lee FC, Ule J. Advances in CLIP technologies for studies of protein-RNA interactions. Molecular cell 2018;69(3):354–369.
2. Jankowsky E, Harris ME. Specificity and nonspecificity in RNA–protein interactions. Nature reviews Molecular cell biology 2015;16(9):533–544.
3. Müller-McNicoll M, Neugebauer KM. How cells get the message: dynamic assembly and function of mRNA–protein complexes. Nature Reviews Genetics 2013;14(4):275.

4. Corcoran DL, Georgiev S, Mukherjee N, Gottwein E, Skalsky RL, Keene JD, et al. PARalyzer: definition of RNA binding sites from PAR-CLIP short-read sequence data. Genome biology 2011;12(8):R79.
5. Van Nostrand EL, Pratt GA, Shishkin AA, Gelboin-Burkhart C, Fang MY, Sundararaman B, et al. Robust transcriptome-wide discovery of RNA-binding protein binding sites with enhanced CLIP (eCLIP). Nature methods 2016;13(6):508.
6. Sullivan KD, Mullen TE, Marzluff WF, Wagner EJ. Knockdown of SLBP results in nuclear retention of histone mRNA. Rna 2009;15(3):459–472.
7. Dobin A, Davis CA, Schlesinger F, Drenkow J, Zaleski C, Jha S, et al. STAR: ultrafast universal RNA-seq aligner. Bioinformatics 2013;29(1):15–21.
8. Krakau S, Richard H, Marsico A. PureCLIP: capturing target-specific protein–RNA interaction footprints from single-nucleotide CLIP-seq data. Genome biology 2017;18(1):240.
9. Quinlan AR, Hall IM. BEDTools: a flexible suite of utilities for comparing genomic features. Bioinformatics 2010;26(6):841–842.
10. Chen X, Chung D, Stefani G, Slack FJ, Zhao H. Statistical issues in binding site identification through CLIP-seq. Statistics and Its Interface 2015;8(4):419–436.
11. Erkmann JA, Wagner EJ, Dong J, Zhang Y, Kutay U, Marzluff WF. Nuclear import of the stem–loop binding protein and localization during the cell cycle. Molecular biology of the cell 2005;16(6):2960–2971.
12. Uhl M, Houwaart T, Corrado G, Wright PR, Backofen R. Computational analysis of CLIP-seq data. Methods 2017;118:60–72.
13. Chakrabarti AM, Haberman N, Praznik A, Luscombe NM, Ule J. Data Science Issues in Understanding Protein-RNA Interactions. bioRxiv 2017;.
14. Dominski Z, Erkmann JA, Yang X, Sànchez R, Marzluff WF. A novel zinc finger protein is associated with U7 snRNP and interacts with the stem–loop binding protein in the histone pre-mRNP to stimulate 3’-end processing. Genes & development 2002;16(1):58–71.
15. Bailey TL, Boden M, Buske FA, Frith M, Grant CE, Clementi L, et al. MEME SUITE: tools for motif discovery and searching. Nucleic acids research 2009;37(suppl_2):W202–W208.
16. Parodi AC, Sangalli LM, Vantini S, Amati B, Secchi P, Morelli MJ. FunChIP: an R/Bioconductor package for functional classification of ChIP-seq shapes. Bioinformatics 2017;33(16):2570–2572.
17. Cremona MA, Sangalli LM, Vantini S, Dellino GI, Pelicci PG, Secchi P, et al. Peak shape clustering reveals biological insights. BMC bioinformatics 2015;16(1):349.
18. Alipanahi B, Delong A, Weirauch MT, Frey BJ. Predicting the sequence specificities of DNA-and RNA-binding proteins by deep learning. Nature biotechnology 2015;33(8):831.
19. McInnes L, Healy J, Melville J. Umap: Uniform manifold approximation and projection for dimension reduction. arXiv preprint arXiv:180203426 2018;.
20. Hartigan JA, Wong MA. Algorithm AS 136: A k-means clustering algorithm. Journal of the Royal Statistical Society Series C (Applied Statistics) 1979;28(1):100–108.
21. Green PJ, Silverman BW. Nonparametric regression and generalized linear models: a roughness penalty approach. Chapman and Hall/CRC; 1993.
22. Akaike H. Information theory and an extension of the maximum likelihood principle. In: Selected papers of hirotugu akaike Springer; 1998.p. 199–213.
23. Huppertz I, Attig J, D’Ambrogio A, Easton LE, Sibley CR, Sugimoto Y, et al. iCLIP: Protein–RNA interactions at nucleotide resolution. Methods 2014;65(3):274–287.

Supplementary Material 1

Click here to access/download

Supplementary Material

Supplements_1.pdf

Supplementary Material 2

Click here to access/download
Supplementary Material
Supplements_2.pdf

Supplementary Material 3

Click here to access/download
Supplementary Material
Supplements_3.pdf

Click here to access/download
Supplementary Material
Supplements_4.pdf

Click here to access/download
Supplementary Material
Supplements_5.pdf

Supplementary Material 6

Click here to access/download
Supplementary Material
Supplements_6.pdf

Click here to access/download
Supplementary Material
main.tex
