# Supplementary material for: StoatyDive: Evaluation and classification of peak profiles for sequencing data
Source: Gigascience. 2021 Jun 18;10(6):giab045. doi: 10.1093/gigascience/giab045 (PMC8212874; doi:10.1093/gigascience/giab045)

Average profiles of the input control.

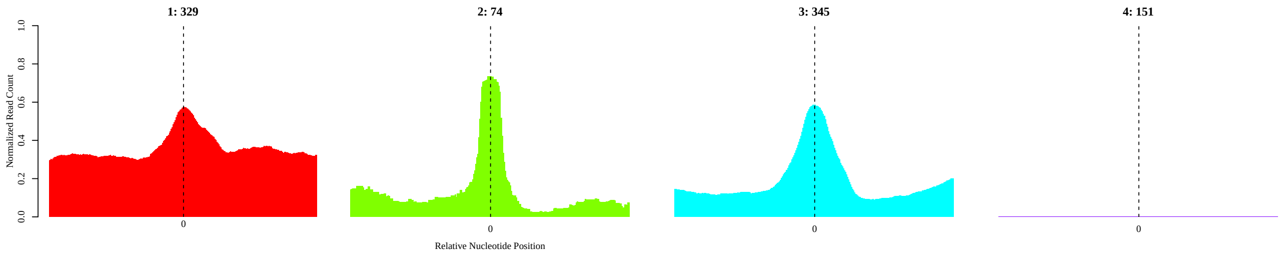

Clustering of the input control.

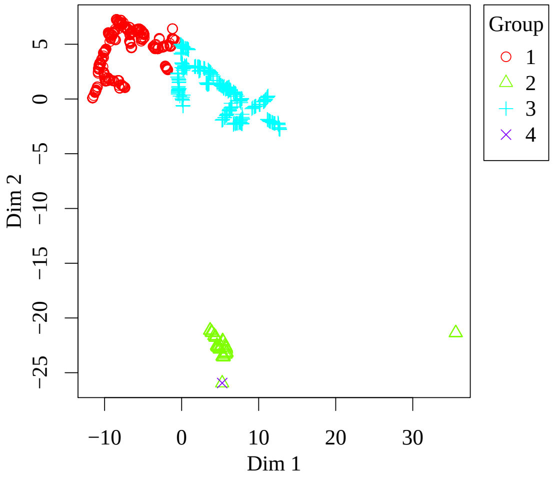

Average profiles of the first replicate of the SLBP data.

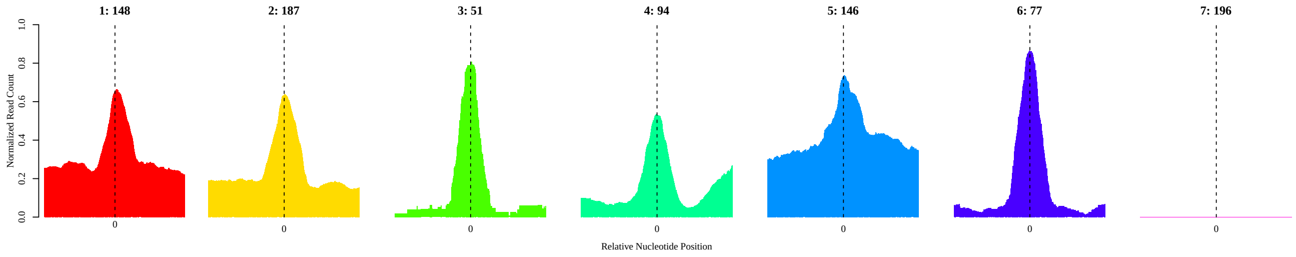

Average profiles of the second replicate of the SLBP data.

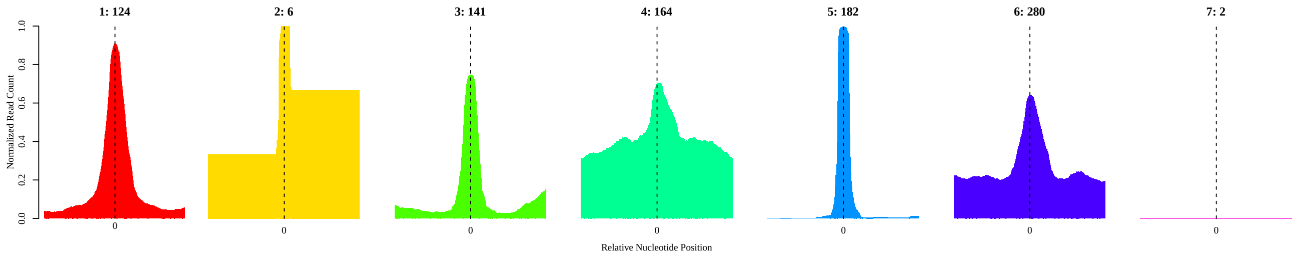

Supplement: giab045_Supplemental_Files [file giab045_supplemental_files.zip › Supplements_2.pdf]
