# Supplementary material for: StoatyDive: Evaluation and classification of peak profiles for sequencing data
Source: Gigascience. 2021 Jun 18;10(6):giab045. doi: 10.1093/gigascience/giab045 (PMC8212874; doi:10.1093/gigascience/giab045)
Supplement: giab045_Supplemental_Files [file giab045_supplemental_files.zip › Supplements_3.pdf]

	All Peaks	Main Cluster	Second Cluster
**30 nucleotides**			
**Mean CV**	0.091	0.046	0.750
**Variance CV**	0.073	0.007	0.600
**Mean LFC**	1.991	2.912	2.233
**Median P-value**	0.024	0.000	0.000
**TPR**	0.453	0.581	0.000
**TNR**	1.000	1.000	1.000
**ACC**	0.724	0.749	0.385
**MCC**	0.539	0.598	0.000
**40 nucleotides**			
**Mean CV**	0.138	0.081	0.065
**Variance CV**	0.120	0.018	0.034
**Mean LFC**	1.989	2.705	0.479
**Median P-value**	0.022	0.000	0.525
**TPR**	0.452	0.544	0.220
**TNR**	1.000	1.000	1.000
**ACC**	0.723	0.731	0.740
**MCC**	0.538	0.573	0.398
**70 nucleotides**			
**Mean CV**	0.322	0.322	**0.217**
**Variance CV**	0.129	0.093	**0.164**
**Mean LFC**	1.898	1.898	**3.433**
**Median P-value**	0.038	0.013	**0.000**
**TPR**	0.483	0.441	**0.690**
**TNR**	1.000	1.000	**1.000**
**ACC**	0.747	0.722	**0.795**
**MCC**	0.568	0.533	**0.657**
**max length (201 nucleotides)**			
**Mean CV**	1.027	1.156	0.594
**Variance CV**	1.158	0.599	0.201
**Mean LFC**	1.960	2.987	1.014
**Median P-value**	0.025	0.000	0.467
**TPR**	0.462	0.528	0.480
**TNR**	1.000	1.000	1.000
**ACC**	0.729	0.703	0.808
**MCC**	0.546	0.542	0.606

	All Peaks	CV<0.2	CV>=0.2	CV<0.5	CV>=0.5	CV<0.8	CV>=0.8
				**30 nucleotides**			
**Mean CV**	0.091	0.025	0.739	0.039	1.203	0.046	1.444
**Variance CV**	0.073	0.001	0.316	0.005	0.227	0.009	0.106
**Mean LFC**	1.991	1.819	3.702	1.901	3.952	1.913	4.405
**Median P-value**	0.024	0.067	0.000	0.055	0.000	0.039	0.000
**TPR**	0.453	0.484	0.259	0.487	0.065	0.483	0.000
**TNR**	1.000	1.000	1.000	1.000	1.000	1.000	1.000
**ACC**	0.724	0.754	0.429	0.751	0.171	0.747	0.040
**MCC**	0.539	0.574	0.272	0.573	0.088	0.568	0.000
				**40 nucleotides**			
**Mean CV**	0.138	0.033	0.695	0.060	1.233	0.079	1.748
**Variance CV**	0.120	0.002	0.376	0.010	0.400	0.020	0.154
**Mean LFC**	1.989	1.644	3.815	1.891	3.376	1.910	4.112
**Median P-value**	0.022	0.081	0.000	0.046	0.000	0.039	0.000
**TPR**	0.452	0.486	0.337	0.488	0.132	0.486	0.000
**TNR**	1.000	1.000	1.000	1.000	1.000	1.000	1.000
**ACC**	0.723	0.761	0.533	0.750	0.353	0.749	0.071
**MCC**	0.538	0.579	0.361	0.573	0.193	0.571	0.000
				**70 nucleotides**			
**Mean CV**	0.322	**0.042**	0.703	0.147	1.043	0.230	1.489
**Variance CV**	0.129	**0.003**	0.410	0.024	0.538	0.055	0.731
**Mean LFC**	1.898	**1.571**	2.296	1.620	2.838	1.855	2.662
**Median P-value**	0.038	**0.108**	0.003	0.070	0.000	0.058	0.000
**TPR**	0.483	**0.607**	0.354	0.533	0.308	0.506	0.212
**TNR**	1.000	**1.000**	1.000	1.000	1.000	1.000	1.000
**ACC**	0.747	**0.825**	0.644	0.781	0.595	0.761	0.509
**MCC**	0.568	**0.679**	0.444	0.614	0.394	0.589	0.304
				**max length (201 nucleotides)**			
**Mean CV**	1.027	0.036	1.269	0.159	1.436	0.326	1.693
**Variance CV**	1.158	0.002	1.141	0.028	1.168	0.075	1.276
**Mean LFC**	1.960	1.615	2.044	1.202	2.317	1.092	2.785
**Median P-value**	0.025	0.035	0.018	0.083	0.008	0.097	0.000
**TPR**	0.462	0.581	0.433	0.462	0.462	0.404	0.502
**TNR**	1.000	1.000	1.000	1.000	1.000	1.000	1.000
**ACC**	0.729	0.783	0.716	0.758	0.716	0.736	0.722
**MCC**	0.546	0.633	0.525	0.566	0.537	0.523	0.556
