# Supplementary material for: StoatyDive: Evaluation and classification of peak profiles for sequencing data
Source: Gigascience. 2021 Jun 18;10(6):giab045. doi: 10.1093/gigascience/giab045 (PMC8212874; doi:10.1093/gigascience/giab045)

Peak lengths of SLBP

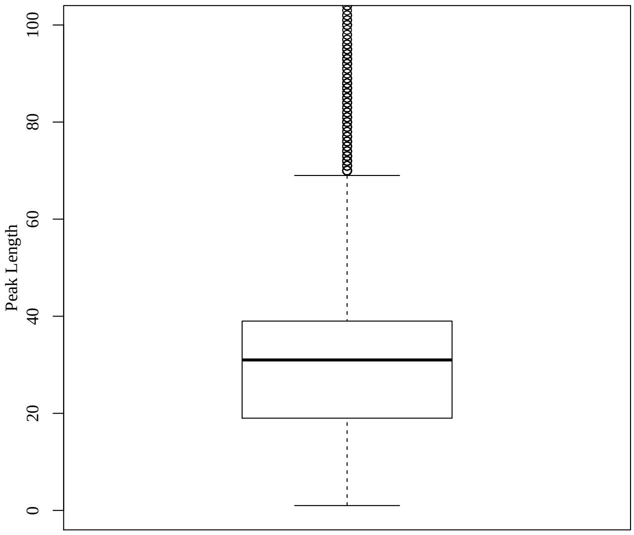

Q1: 19, Median: 31, Q3: 39, IQR: 20

Peak lengths of the proteins analyzed for the fold changes between sharp versus broad peaks.

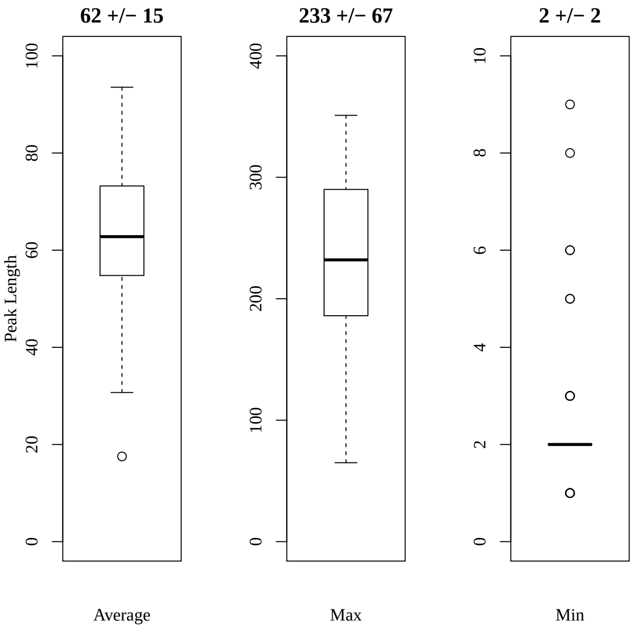

Supplement: giab045_Supplemental_Files [file giab045_supplemental_files.zip › Supplements_4.pdf]
