# Supplementary material for: StoatyDive: Evaluation and classification of peak profiles for sequencing data
Source: Gigascience. 2021 Jun 18;10(6):giab045. doi: 10.1093/gigascience/giab045 (PMC8212874; doi:10.1093/gigascience/giab045)

Dimensional reduction with a PCA.

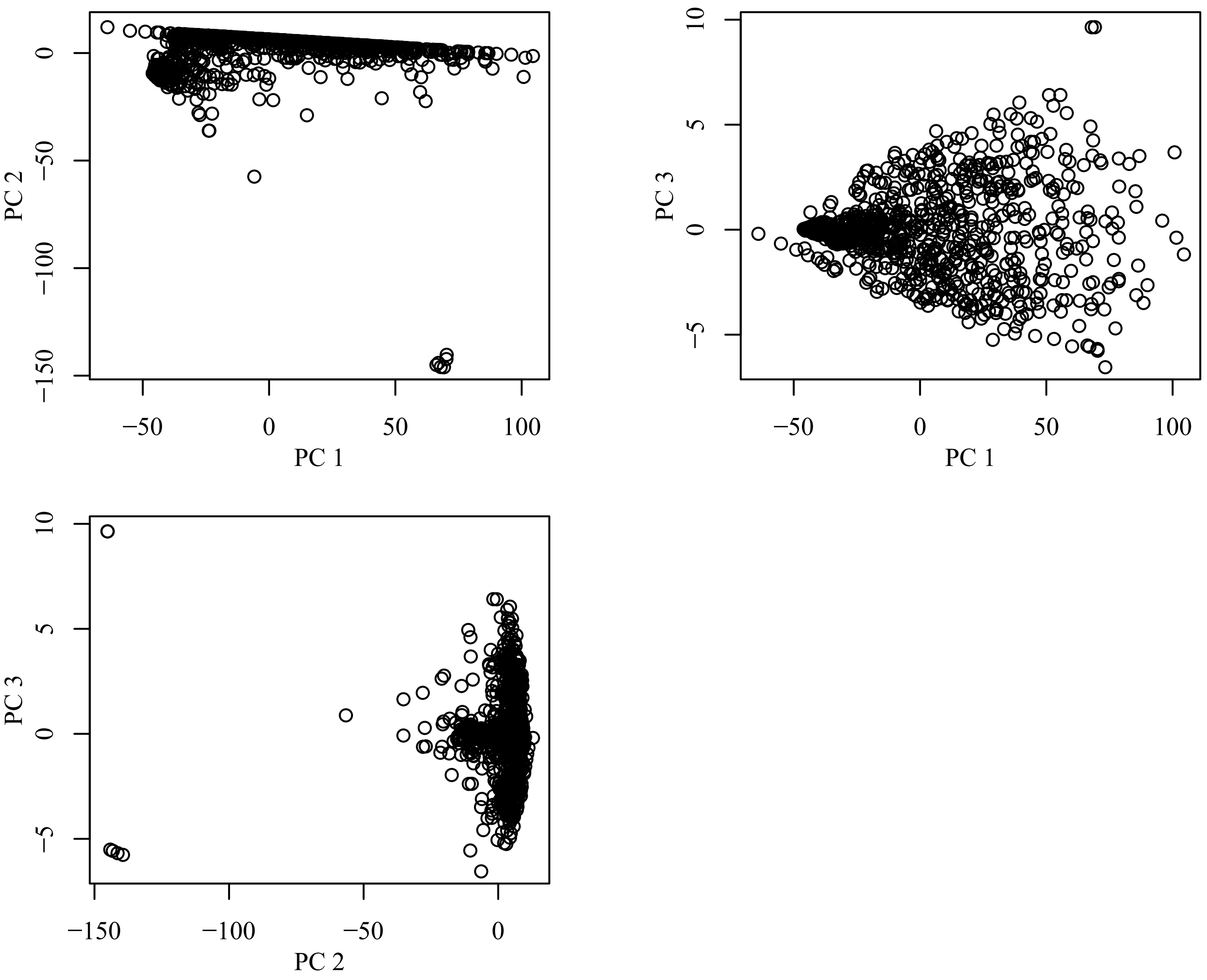

Dimensional reduction with a SOM.

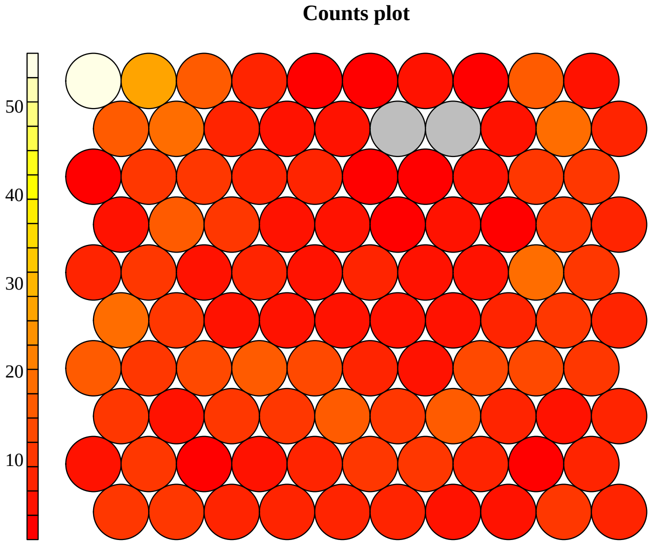

Dimensional reduction with t-SNE.

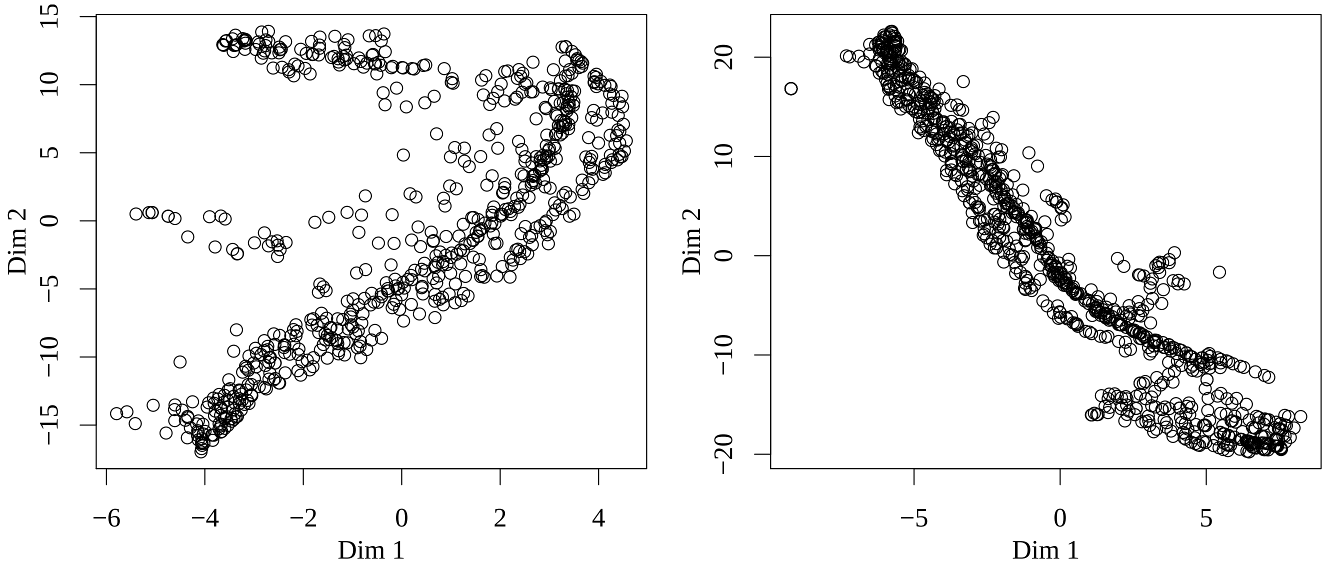

Supplement: giab045_Supplemental_Files [file giab045_supplemental_files.zip › Supplements_6.pdf]
