# Supplementary material for: StoatyDive: Evaluation and classification of peak profiles for sequencing data
Source: Gigascience. 2021 Jun 18;10(6):giab045. doi: 10.1093/gigascience/giab045 (PMC8212874; doi:10.1093/gigascience/giab045)
Supplement: giab045_Response_to_Reviewer_Comments_Original_Submission [file giab045_response_to_reviewer_comments_original_submission.pdf]

Author's Response To Reviewer Comments

Close

\section{Reviewer 1}

Major comments:

1) I find that there's an imbalance where the most important findings are stated very briefly (sometimes without figures), whereas significant text and figure space is devoted to more descriptive analyses that lack a clear conclusion.

For example, to me the most important finding from the method is that the use of different peak shapes enables separation of real versus artifact peaks, which is aided by the authors use of SLBP (which has a well-characterized specific role in binding a specific hairpin structure in histone 3' UTRs), However, this result is stated in Table 1 without statistical analysis (enrichment, significance, etc) and a couple sentences of text. The manuscript would be stronger with additional analysis and description here (what is the sensitivity/specificity tradeoff in using the shape group / CV cutoffs described here? How do those change with different cutoffs?)

\begin{answer}
Thank you very much for your suggestion as it contributed a lot to the paper.

PureCLIP already filters for significant peaks and does not give any enrichments (no fold changes or p-values). Thus all peaks of SLBP that are mentioned in table 1 are significant peaks. So it was not possible to define sensitivity/specificity or other features for table 1.

We made the effort and applied the peak shape clustering on the CLIPper peaks for replicate 2 (all peaks). Therefore, we add the new Supplements 3 in the paper and a whole new section called "Optimizing StoatyDive with the data of SLBP", where we go deeper about StoatyDive based on different clusters, CV cutoffs and peak sizes.

As a short summary, we show that the best CV cutoff is at 0.2 and that the cluster has to be chosen carefully in order to reduce some noise. We also show, that we can remove some noise from the initial CLIPper peak set. Furthermore, we demonstrate that the best peak length for StoatyDive is around 70 nucleotides.

The section includes more findings, and we would invite you to read the new section.

In addition we included also more statistical analysis to clarify our findings. So we state for the different CV distributions between the replicates and the control of the SLBP data: "Although the CV distributions of the input control and replicate 1 of the SLBP data differed significantly (one-sided Wilcoxon test p-value = 0.03), both contained a lot of regions with a CV close to zero (Figure 2, both with a mean CV of 0.47). In contrast, the CV distribution of replicate 2 was distinct (p-value < 0.05 to input control and replicate 1) since it had more peaks with a higher CV (mean CV of 1.41) and thus more specific binding events (e.g., Figure 1a, $CV \approx 5.3$)."

We also calculated the ACC for the tool comparison: "StoatyDive classified most peaks correctly into the three peak shape groups (Figure 5a, ACC = 0.87)." and later "SIC-ChIP identified up to six different peak shapes (Figure 5b), whereas FunChIP found three (Figure 5c and d, ACC = 0.6)."
\end{answer}

In contrast, two paragraphs are devoted to a fairly superficial motif analysis - it's not clear from the text which motifs the authors believe are real and which are false-positives, which makes it descriptive but unclear what the conclusion is (other than that they are 'different'). Although this does not appear to be discussed in the text, it seems that the GCUCUUU motif matches the known SLBP hairpin (e.g. Fig 2 from Tan et al, PMID 23329046), which would again suggest that the sharp and plateau classes are real

whereas the broad class motifs are artifacts; this should be more clear.

\begin{answer}
So far we do not believe any of the motifs are true or false. We initially started to analyse SLBP and tried to show that different sequence motifs can be found with different peak shapes. SLBP is still a good candidate to show, that different peak shapes are either artifacts or real binding sites. Yet, it is important that the user checks the biological correctness of those clusters also for other proteins. We made a statement in the paper to clarify this issue by noting:

"Yet, we cannot confirm the biological truth behind those motifs as it requires further experiments to verify them."
\end{answer}

2) The authors use the ENCODE data, but then re-perform peak calling with PureCLIP to perform their analyses. This isn't necessarily a problem; however, in the context of the major point of the manuscript being discussion of peak shapes, I find it perplexing that this is done with no further discussion, as one of the main differences between PureCLIP and the CLIPper algorithm (which was used in the peak calls provided by ENCODE) is that CLIPper explicitly uses peak shape as a feature to perform spline-fitting as part of the cluster identification process (Lovci et al. PMID 24213538). I can thus see an argument for using a peak caller that does not incorporate this feature in order to enable downstream analyses comparing peak shape; but I do think there should be some ultimate discussion of whether the classification and clustering described here provides additional power over the spline-fitting used in the initial CLIPper calls.

\begin{answer}
We showed and analyzed also the CLIPper peaks (peaks which are significant and not significant) of SLBP in the paper. We demonstrated that also CLIPper's results could benefit from StoatyDive. Furthermore, the intention behind StoatyDive is an analysis independent of the peak caller. Of course it can be used to remove potential artifacts of the data processing and peak calling, but it is also worth to investigate a set of significant peaks with StoatyDive. You can cluster those peaks in different groups, which might reflect different biological properties. That to say, peak shape clustering comes after the peak calling and is a refinement or selection step for a deeper analysis of the peak set.
\end{answer}

Specific comments:

The authors conclude a hypothesis that "peak profiles shaped like plateaus were mainly PCR duplicates that were less informative." - this is a conclusion that is not supported by data, but is both confusing (the eCLIP datasets contain unique molecular identifiers and thus shouldn't contain significant PCR duplicates) and also is easy to test from the data used (those peaks should have higher mutation rates or PCR duplication rates among pre-PCR-duplicate-removed reads than other peaks).

\begin{answer}
We removed this statement as we saw that those peaks are indeed not PCR duplicates and basically a distinctive shape of the data.
\end{answer}

I'm confused by the sentence "We looked further and found sharper peaks located on stem loops targeted by SLBP such as RNU7-1 RNA ..." - do the authors believe these are real or artifact peaks? Either way there should be some discussion of why (and why they are specifically called out here)

\begin{answer}
RNU7-1 is a potential target of SLBP. We have removed the other stem loop protein from the paper because we could not find any literature to verify it as a target of SLBP. The other stem loop was an assumption based on our own past findings.

The paper now states: "For example, we found a sharper peak located on RNU7-1 RNA (U7 small nuclear 1) that contains a stem loop that might be potentially targeted by SLBP [citation]. The peak got a CV of 3.9 and was classified into the peak profile cluster 3 of replicate 2."
\end{answer}

Many figures need editing to be more visually clear. In addition to fonts (Fig. 3 and 4 in particular have

multiple panels that illegibly small at standard resolution), the figures would be significantly clearer if additional labels are provided to enhance flow through the analyses in the paper. For example - Table 1 refers to separation of the 7 classes in Fig. 3c/d into 4 shape 'groups'; it would be helpful to have those labeled in Fig 3 as well.

\begin{answer}
We included your recommendations and changed the font as well as layouts for all figures. Therefore we invite you to check the new figures. We hope we could improve the readability and intuitive understanding.
\end{answer}

I think it needs to be clearer exactly what the analysis being done in the last section (of other RBPs) - after multiple reads I'm still a bit confused how the earlier analysis (with unsupervised clustering and manual annotation of classes) connects with the last analysis (with a hard cutoff of 'sharp' vs 'broad' peaks). The text in this section (Fig 7) refers to 'broader' versus 'sharper' shaped peaks; however I'm not clear on what defines those terms (the methods text suggests that for standard analysis the authors set a CV cutoff of 0.5 to define 'specific' versus 'unspecific'; I'm assuming but not clear that it's that and not manual annotation of unsupervised clustering classes as was done for SLBP?)

\begin{answer}
It is true, we have not done a manual classification into \textbf{broad} and \textbf{sharp} as we have done for the data of SLBP. SLBP was a detailed example and finer analysis, whereas the last part was done more automatically. We stayed very conservative and looked at the CV distribution of each protein to find a good cutoff for broad vs sharp. It is to note that the CV distribution depends on the size of the peaks and the protein being analyzed. The initial cutoff of 0.5, was done on initial observations. Now, we know that a very conservative cutoff is at 0.2. We state in the paper in section "Investigation of eCLIP Protein Profiles":

"All peaks were therefore extended or shrunk to a length of 77 nucleotides. This was based on the observation that the third quartile of all peaks from all proteins was 77 nucleotides long (see Supplementary Figure 4). In addition, StoatyDive achieved for the SLBP data a better TPR and MCC with a peak size of 70 and a CV threshold of 0.2 (see Supplementary Figure 3). The results of SLBP showed that it may be wise to combine the clustering and the CV threshold to assess the profile landscape of other proteins. We therefore defined a peak as sharp if it had a CV > 0.2 and it fell into a cluster that was generally sharper. A cluster was declared as sharp if the median CV of the cluster was bigger than the median CV of the whole peak set. All other peaks were classified as broad."
\end{answer}

\section{Reviewer 2}

1. comment: I am surprised that the authors focused their analyses on the SLBP protein and not on the more well studied RBP.

\begin{answer}
It is true that there is no information about a sequence or structure motif for SLBP, but it is well known (see reviewer 1), that SLBP binds distinctively histone mRNAs at the 3' UTR region. Thus, it was interesting to see if we can find differences in peak shapes and show that we can differentiate between specific and unspecific binding with StoatyDive. Furthermore, it was interesting to see if we can find other distinct binding shapes for SLBP that might reflect different biological mechanics since the protein has two functions in eukaryotic cells, serving as transport and translation factor. SLBP is one of the proteins that can be used to optimize CLIP-Seq data analysis and is therefore a good candidate for StoatyDive.
\end{answer}

However, the authors did find interesting observations for the SLBP protein by analysing eCLIP samples in K562 cell lines, including low reproducibility between the replicates, which haven't been fully investigated in this manuscript. I think it would be important to address these observation in a more systematic manner:
* Have you checked how much the Coefficient of Variation (CV) changes between other replicates in other eCLIP samples, including eCLIP HepG2 samples? It would also be important to include CV results

for all other samples that you analysed in this study (RBM22, U2AF2, PTBP1…). Do other eCLIP samples also show similar differences between the replicates or is the SLBP protein one of the exceptions?

\begin{answer}
We included the CV distributions of the other proteins for the K562 cell line in the supplementary of the paper (Supplementary Figure 1) and made a short statement in the section "Peak Profile Landscape Reveals Low Reproducibility of Binding Sites", where we state: "Checking the CV distributions of other CLIP-Seq datasets such as TAF15, TARDBP, and HNRNPA1, which we will analyze further in a later section, (Supplementary Figure 1), we saw that the CV distributions also had differences between the replicates (two-sided Wilcoxon test p-value < 0.05). However, this does not mean a low quality of the data and just highlights that it is important to do replicates in order to quantify biological and technical variance as noted in a previous CLIP study [citation]."

We think that this is already enough for a demonstration that the CV might vary between replicates. An even deeper analysis including the HepG2 cell line is beyond the scope of the paper.
\end{answer}

Is this variation between replicates more specific for the eCLIP protocol or is it also common in other CLIP-seq methods such as iCLIP, irCLIP or PAR-CLIP?

\begin{answer}
A variation between replicates can of course happen (see AC Jungkamp et al. (2011), In vivo and transcriptome-wide identification of RNA binding protein target sites, and X. Chen et al. (2015), Statistical issues in binding site identification through CLIP-seq). This can come from different sources: execution of the experiment, changes in lab staff, technical variances, biological variances and more. It is hard to figure out what the actual reason was, especially since we used data that we did not generated in our group. It is beyond the scope of this paper to investigate other CLIP protocols, since we wanted to check the power of StoatyDive. However, we would analyze this in the future, when we will update StoatyDive and give it even more power.
\end{answer}

* How many peaks identified in replicate one overlap with replicate two?

\begin{answer}
Replicate one had 659 peaks and replicate two 935. The overlap is the mentioned 899 peaks. We made a robust peak detection (intersecting and merging the two peak sets) to define a robust set of peaks. So we use these 899 peaks to evaluate both replicate one and replicate two. Replicate two has perhaps more peaks, but some of those peaks can be merge into a bigger peak region that is covered by one peak of replicate one.

From those peaks, we have 236 exonic and 151 intronic peaks for replicate one, and 374 exonic and 289 intronic peaks for replicate two. So replicate two has for both 136 exonic and 136 intronic peaks more. So there is a difference in the number of peaks between replicate one and two but the difference between exonic and intronic is the same.
\end{answer}

* Is the library size similar between the replicates and have you done any more detailed quality controls between the replicates (number of uniquely mapped reads, sequencing errors etc.)? Maybe a poor quality or low coverage could explain the difference.

\begin{answer}
We checked the raw data quality and there are no quality breeches, except the high duplication level and adapters you usual see in CLIP-data.

Furthermore, the supplementary data table by Van Nostrand et al. (2016, Robust transcriptome-wide discovery of RNA-binding protein binding sites with enhanced CLIP (eCLIP)) state for the aligned files:

\textbf{Replicate 1}\\
\# Input read: 13,553,232\\
\# Uniquely mapped read: 3,558,228\\
\# Usable read: 2,664,051\\
\% usable (out of uniquely mapped): 0.748701601\\ \\

\textbf{Replicate 2}\\
\# Input read: 15,493,437 \\
\# Uniquely mapped read: 3,354,237 \\
\# Usable read: 2,520,409 \\
\% usable (out of uniquely mapped): 0.751410529

So the library sizes are almost the same.
\end{answer}

* Would it be possible that one replicate has more cytoplasmic binding sites than the other? One way of testing this would be to check how many peaks that you identify are in intronic regions vs exonic regions, for each replicate separately. Also, how does the CVs and shape profile of the peaks change between exonic and intronic peaks? Maybe this sample has a higher binding specificity in the nucleus than in cytoplasm or vice versa.

\begin{answer}
Thank you very much for this interesting question and idea.

Indeed there was a big difference for exonic and intronic peaks. We dedicated a new chapter in the paper, where we state: "To investigate further differences between the two replicates, we split the peak set into peaks overlapping with exons and introns (see Figure~3). SLBP is a translation and transport factor, which is present in the cytoplasm as well as nucleus [citations]. Therefore, the replicates could have different binding events, where one replicate might have more events in cytoplasm and the other more in the nucleus. For replicate 1 we got 236 exonic and 151 intronic peaks for replicate 1, and 374 exonic and 289 intronic peaks for replicate 2. So replicate 2 had 136 exonic and 136 intronic peaks more. Notably, we can see a huge CV difference when comparing the intronic peaks of the two replicates, with a mean CV of 0.23 for replicate 1 and 1.26 for replicate 2 (one-sided Wilcoxon test P-value < 0.05). The exonic peaks on the other hand were more similar (mean CV = 0.48 and 0.90, respectively), but still the CV distributions were significantly different (one-sided Wilcoxon test P-value < 0.05). It is not clear why this difference appeared, but our observations stress out how important it is to perform an experiment with replicates."
\end{answer}

2. comment: "It is important to note that peaks shaped like plateaus might be false positives. These peaks most likely corresponded to PCR duplicates that were not real binding sites. We have removed PCR duplicates during the pre-processing of the read library, but some duplicates might still be in the data. Sequencing errors in the unique molecular identifiers (UMI) are a common reason."
* Are there any changes in shape profiles between intronic and exonic peaks? To filter out false positive peaks, you could use ENCODE RNA-seq data for the same cell line and analyse peaks and their shapes in expressed transcripts. How many False positives are present in non-expressed transcripts and do they show different motifs and different shapes of their peaks?

\begin{answer}
We removed this statement in the paper as we observed that those peaks are not PCR duplicates and a distinct peak shape.

While your suggestion still pose an interesting question for future research, it goes beyond the scope of this paper and we would do this part with a newer version of StoatyDive. We want to expand StoatyDive with a comparison mode to allow two or more samples to be compared (cluster them together) in their profile shape landscape (e.g., control vs CLIP). This would give a better distinction of false positives.
\end{answer}

3. comment: "We investigated also the sequence motifs of sharp and broad peak profiles of the protein RBFOX2 (eCLIP data from the study by Van Nostrand et al. [5]), because it has the conserved sequence motif TGCATG, which is enriched in the RBPFOX2's binding sites [13, 14, 15]. We have found the conserved motif only in sharp peaks (Table 3), but the broader profiles also had an enriched motif. It is worth investigating if RBFOX2 has some unspecific binding preferences with that specific motif. The results for RBFOX2 again demonstrated that different peak shape groups result in different sequence motifs."
* A recent paper by Bridget E. Begg et al. identified secondary binding motifs from their RBFOX2 CLIP experiments (Begg et al. 2020). These motifs are specific for intronic and 3'UTR regions (Bridget E. Begg

et al., Figure 3). Could the authors also identify these motifs by separating their peak shape profiles into intronic and 3'UTR region?

\begin{answer}
Thank you very much for this questions, however we have removed this chapter from the paper as it was too short and disrupted the flow of the paper.

We did a short analysis for your question, but there where no interesting results or significant differences of the shapes between exonic, intronic and 3'UTR regions.
\end{answer}

4. comment: "All peaks were therefore extended or shrank to a length of 77 nucleotides. This was based on the observation that the third quartile of all peaks (from all proteins) was 77 nucleotides long."
* The 77 nucleotides window size for all RBPs could be too broad to identify sharp peaks for proteins with specific bindings. Anob M. Chakrabarti et al. has shown in their review (Chakrabarti et al. 2018), that the window size parameters can modify the sensitivity and specificity of the data and it needs to be adapted for each RBP. For example, PTBP1 can have multiple binding sites just next to each other, where the broad window size would detect all the protein binding sites as one large binding site instead of multiple sharp ones. Could the authors demonstrate and comment on at least one example of how the window size can affects peak finding specificity and their shape profiles?

\begin{answer}
It is true that different window sizes affect the results. We did an additional analysis to show the effect of the peak length. We dedicated now a complete new chapter "Optimizing StoatyDive with the data of SLBP", where we go deeper about StoatyDive based on different clusters, CV cutoffs and peak sizes.

As a short summary, we show that the best CV cutoff is at 0.2 and that the cluster has to be chosen carefully in order to reduce some noise. We also show, that we can remove some noise from the initial CLIPper peak set. Furthermore, we demonstrate that the best peak length for StoatyDive is around 70 nucleotides.

The section includes more findings, and we would invite you to read the new section.

A size of 77 nucleotides might be long, yet it is good to discriminate the profiles that you have mentioned. We state later in the paper now:
"All peaks were therefore extended or shrunk to a length of 77 nucleotides. This was based on the observation that the third quartile of all peaks from all proteins was 77 nucleotides long (see Supplementary Figure 4). In addition, StoatyDive achieved for the SLBP data a better TPR and MCC with a peak size of 70 and a CV threshold of 0.2 (see Supplementary Figure 3). The results of SLBP showed that it may be wise to combine the clustering and the CV threshold to assess the profile landscape of other proteins. We therefore defined a peak as sharp if it had a CV > 0.2 and it fell into a cluster that was generally sharper. A cluster was declared as sharp if the median CV of the cluster was bigger than the median CV of the whole peak set. All other peaks were classified as broad."
\end{answer}

5. comment: "So we investigated the number of peaks that fall into introns (90\% overlap) for the proteins that are involved in the splicing process. The proteins PTBP1 (85\%), RBM22 (62\%), TARDBP (79\%), and HNRNPM (87\%) had more than 50\% of peaks in introns, which detected the assumption of split peaks. However, the proteins U2AF1 (17\%), and U2AF2 (15\%) had more peaks in exon regions, where the possibility of split peaks might still occur. We found for U2AF1 only 27 peaks (0.72\%) and for U2AF2 5 peaks (0.40\%) that are potential split peaks, again defecting the assumption of a technical artefact in the peak set of splicing factors. Thus, the sharpness of the peaks of U2AF1 and U2AF2 was potentially not the result of the peak calling."
* The authors examined RBPs that are involved in the splicing process, including U2AF1 and UAF2, which recognises 3' splice sites and recruits the spliceosome. The authors claim that the majority of the identified peaks from both proteins fall into the exonic region. This statement contrasts with previous studies including my preliminary analysis (see Figure ??? below). The figure shows a normalised coverage of cDNA-starts for the same eCLIP U2AF1 and U2AF2 K562 and HepG2 samples relative to the 3' splice site region, showing a dominant enrichment of the intronic region for both proteins which also agrees with the previous CLIP studies (Zarnack et al. 2013; Sutandy et al. 2018; Briese et al. 2019). The authors should examine their peak analyses in these results further to identify the reason for such a difference.

\begin{answer}
Thank you for your suggestion. It was not our intention to make this claim. We just wanted to investigate a potential shape bias, because of a peak phenomenon that we called "split peaks". We state now in the paper: "We also checked whether our result that RBPs involved in
splicing have sharper peaks can have technical reasons. In this case, a splicing related protein could have more peaks that are split over two exons, which are detected by the peakcaller as two separate but sharp peaks (split peak). So we investigated the number of peaks that fall into introns (90% overlap) for the proteins that are involved in the splicing process. We used bedtools set to a strict overlap (intersect -u -s -f 0.9) to investigate potential split peaks." and later "It is important to note that this does not mean that the aforementioned proteins bind generally more introns or exons."

We checked and assume that our constraint for bedtools with 90% overlap is the reason for this different observation to your findings. When we decrease the overlap, more peaks overlap with introns.
\end{answer}

\section{Reviewer 3 -- finished}

* Figure 2 indicates that replicate 1 of SLBP CLIP-seq is indistinguishable from the negative control from the perspective of the coefficient of variation. However, this replicate is then used throughout the analyses of Figure 3 and Tables 1 and 2. If Replicate 1 did work experimentally, it would contradict the authors' proposal that evaluating the coefficient of variations across peak-profiles (Figure 2) is a valuable method of examining CLIP-seq quality control.

\begin{answer}
Thank you for your question. We state in the paper: "Although the CV distributions of the input control and replicate 1 of the SLBP data differed significantly (one-sided Wilcoxon test p-value = 0.03), both contained a lot of regions with a CV close to zero (Figure 2, both with a mean CV of 0.47). In contrast, the CV distribution of replicate 2 was distinct (p-value < 0.05 to input control and replicate 1) since it had more peaks with a higher CV (mean CV of 1.41) and thus more specific binding events (e.g., Figure 1a, CV ≈ 5.3). Yet, some potential binding sites were more unspecific with a CV closer to zero (e.g., Figure 1b, CV ≈ 0.006)."

Even though the distribution between the input control and replicate one looks quite similar the p-value is < 0.05, thus replicate one is significantly different in the CV distribution in comparison to the input control.

Furthermore, the CV distribution is just one quality factor for a CLIP experiment. We state in the paper: "The CV is just one quality factor and we recommend to test other features as well, such as the read coverage correlation."
\end{answer}

* Figure 3 shows the distinct patterns present across two replicates of SLBP CLIP-seq. It is unclear how unique these patterns are to SLBP CLIP-seq or if they are an artifact of CLIP-seq assay. It would be important to show the average tag distribution of the control experiment at sample peaks (separated by distinct peak profile) in order to demonstrate that the discovered peak profiles exist only within sample-targeted CLIP-seq.

\begin{answer}
We included the results of the input control in the paper and state: "We also checked the uniqueness of the shapes by analyzing the peaks based on the reads from the size-matched input control (see Supplementary Figure 2). StoatyDive had just identified four different clusters, encompassing mountain shaped peaks, as well as plateaus, and constant peaks. It was to be expected to find similar shapes in the control, because the biggest challenge for peak calling is the identification of enriched sites with different shapes between control and CLIP data [12, 13]. In a future version of StoatyDive, we will include a mode to check peak shapes between samples to see if we could improve peakcalling results with a peak shape comparison."
\end{answer}

* * The underlying RNA sequence of the peaks should also be investigated with regards to whether

mappability (or a specific A/U/G/C content) is responsible for producing broad vs narrow peaks. Are broad peaks a function of less mappable reads producing unnatural peaks? It would important to demonstrate that StoatyDive is not just modeling the underlying sequence mappability at the region.

\begin{answer}
We can immediately excluded this because we have used only significant peaks from CLIPper and PureCLIP. PureCLIP searches for significant peaks (combining both CLIP replicates) with an HMM model. CLIPper from the CLIPper pipeline is run on each replicate separately and both peaks sets are then tested for robustness with an irreducible discovery rate (IDR) analysis. That is to say, mappability and low quality of the peaks can be excluded since (a) only significant and robust peaks are used from the peakcalling, and (b) only high-quality (quality score $>$ 20) and unique mappable reads were used. So the comparison of broad and narrow peaks really is just done on the CLIP-Seq enrichment.
\end{answer}

\section{Final remark:}

We thank all reviewers for their comments, critics, ideas and questions. It was very helpful and improved the paper a lot. You will find changes in the paper marked in red. Furthermore we added a lot of new supplements. Please take a look. Thank you very much for your time and effort.

Close
