# Supplementary material for: StoatyDive: Evaluation and classification of peak profiles for sequencing data
Source: Gigascience. 2021 Jun 18;10(6):giab045. doi: 10.1093/gigascience/giab045 (PMC8212874; doi:10.1093/gigascience/giab045)
Supplement: giab045_Response_to_Reviewer_Comments_Revision_1 [file giab045_response_to_reviewer_comments_revision_1.pdf]

Author's Response To Reviewer Comments

Close

\section{Reviewer 1}
I appreciate the effort of the authors to provide additional analyses and address previous concerns, and the sections with specific edits are greatly improved. However, while the authors have clarified in many locations, there remain others (especially in the added sections) where it remains very confusing exactly what dataset is being used for analyses or plotted in figures. I also have remaining concerns (particularly about the newly added sections), where the authors seem to assume by default that their CV metric is accurate and reliable and then use that to draw conclusions about the underlying data, but I think this assumption is something that needs to be shown by analysis in the paper and cannot be assumed.

I think the new section on replicates "Peak Profile Landscape Reveals Low Reproducibility of Binding Sites" is weak and detracts from the paper. Broadly, my major concern is that it's completely unclear whether the low reproducibility seen is a feature of the experimental eCLIP data or a technical property of their CV metric. Essentially, this section defines a new metric (the CV for each peak), sees low reproducibility in this metric across replicates, and then assumes that this must imply low reproducibility in data "However, we have to stress out that we are unsure if this is the result of the specificity of SLBP, the different quality of replicate 1 and 2, or a different source, for example, a bad immunoprecipitation." To me, this statement requires some sort of analysis (downsampling / replicate splitting?) to show what the reproducibility of their CV metric is to random sampling variability. It would also be helpful to show here real examples of peaks that the authors claim show low reproducibility across replicates, to visualize what exactly their analysis is claiming.

\begin{answer}
Thank you very much for your suggestions. We have changed the term "low reproducibility". We rephrased the title to "Peak Profile Landscape Reveals Variability of Binding Sites". We also rephrased the chapter saying: "The distribution was an indicator for some variability in the binding events. This was also striking when we compared the distribution of replicate 1 and 2. However, we have to stress out that this does not mean a poor quality of replicate 1 and 2. For a downstream analysis, for example the prediction of sequence motifs, it is worth investigating why the CV distribution of replicate 1 was very different to replicate 2. A sequence motif prediction depends on the selected binding sites, thus StoatyDive's inspection allows the user to assess the binding sites using the CV distributions. This helps to appraise if SLBP has protein domains that bind to RNA in an unspecific manner. The user can also test if the unspecific peak Figure~1b might be a false positive of PureCLIP."

To clarify, we wanted to investigate the variability of the binding events that are common for both replicates. Therefore we merged all binding sites that we have obtained from replicate 1 and 2. So we have a set of 899 peaks. We looked how these 899 peaks behaved (peak profiles shapes) for replicate 1 and 2. We took also the same 899 peaks and analyzed the profiles shapes for the control data to see if the shapes are similar to the replicates (reference point).

The chapter was designed to investigate if differences in the peak profiles occur on a set of common binding sites for the replicates. This is crucial, because in the end, the user has to know if different shapes occur for different replicates and that this variability can be inspected with our tool, which ultimately leads to a better down-stream analysis.
\end{answer}

In addition, the conclusion that their analysis indicates that the replicates are of differing quality is also a computationally testable question (for example, comparing the fold-enrichment at previously validated histone 3'UTR peaks), which would help to convince that this isn't simply reflecting low reproducibility of their metric itself. The authors do this somewhat in Sup Fig 1, but then just use that to conclude that many other RBPs are also variable across replicates; SLBP definitely seems worse than those other RBPs, but this difference should be quantified. If the authors simply mean 'there is high variability in the CV metric between replicates for some RBPs', this should be worded as such and avoid claims about

reproducibility of the underlying data without further analysis.

\begin{answer}
We have removed the word reproducibility as stated in our first answer. It is true, we wanted more to clarify that there is some variability in the CV between replicates.
\end{answer}

I'm also unclear what the 'control' dataset used in Figure 2 (and some later analyses) is exactly - the author state that they ran PureCLIP "for each CLIP replicate separately, taking the input control into account", so what is the control peak set in Figure 2 (as I assume it can't have been PureCLIP run on the input with itself as a control)?

\begin{answer}
Please refer to the first answer for the explanation.
\end{answer}

The conclusions here are also vague and non-specific - "For a downstream analysis, for example the prediction of sequence motifs, it is worth to either exclude replicate 1 or to investigate,
why the CV distribution of replicate 1 was very different to replicate 2. Thus given the inspection of StoatyDive, the user can now decide to investigate the binding sites of replicate 1 and compare them with the input control or replicate 2." - why was it worth investigating replicates separately? I'm unclear what the authors think was improved by performing this analysis - they show that the replicates have differences in CV distribution, with replicate 1 resembling the control (as above, I don't understand what this control peak set represents), but I don't see anything other than the CV analysis to indicate that Rep1 is of worse quality than Rep2 (in peak enrichment for histone RNAs, motifs, peak enrichment above input, etc).

\begin{answer}
We rephrased the statement, which we explained in the first answer. Indeed it is better to point out that the user just observes a variability in the CV distribution between the replicates, which does not say anything about the reproducibility or that replicate 1 should be excluded from further investigations.
\end{answer}

Similarly, the authors then split the peaks by exonic and intronic and claim "It is not clear why this difference appeared, but our observations stress out how important it is to perform an experiment with replicates." - how do they stress this? I
don't see any benefit that the replicates have provided beyond the ability to say that they're different; other than Replicate 2 having more peaks, it's not described whether those additional peaks are real or false positives, and thus whether replicate 2 is better or worse.

\begin{answer}
We have rephrased this sentence slightly: "It is not clear why this difference appeared, but StoatyDive showed a variability between the different peak sets."

It was not our intention to state that replicate 2 was better than replicate 1. We thought the sentence benefits the data since it had replicates. Our results showed that some variability in the binding events might occur and highlighted that it was very good that SLBP had two replicates.
\end{answer}

I similarly think there needs to be improved clarity in the "Optimizing StoatyDive with the data of SLBP" section. I don't understand this sentence "This was achieved by the second cluster, which pointed out that the cluster had to be carefully chosen in order to remove some noise in the data (artifacts)." - does this mean that the authors removed some peaks in this set? Moreover, what are 'main cluster' and 'second cluster'? I don't see these defined anywhere.

\begin{answer}
In the section "Optimizing StoatyDive with the data of SLBP" we wrote: "We investigated the cluster with the highest (Main Cluster) and second highest (Second Cluster) number of peaks in histones." So basically we just looked at each cluster and checked how many peaks in this cluster overlap with histones. Ideally the peaks that overlap with histones should be contained in the main cluster but StoatyDive is of course not flawless and perhaps the profile shape is different for some histone peaks. That is why we investigated also the cluster (second cluster) with the second highest number of peaks

overlapping with histones.

Main and second clusters are therefore just different subsets of the entire peak set.
\end{answer}

Similarly, "Furthermore, the same size achieved the highest TPR (0.61) and MCC (0.68) with a CV threshold of 0.2. However, the enrichment was not as good as with the clustering resulting in a mean LFC of 1.6 (median P-value = 0.108)." - I am completely lost as to what 'the same size' or 'the clustering' are referring to here in terms of datasets. This section needs to be edited to clearly state what exact set of peaks is being referred to at each point.

\begin{answer}
We rewrote the paragraph saying: "\textcolor{red}{Furthermore, the same peak length of 70 nucleotides achieved the highest TPR (0.61) and MCC (0.68) when we filtered the peaks based on a CV threshold of 0.2. However, the enrichment was not as good as with the clustering by StoatyDive, where we achieved a mean LFC of 3.4 in the second cluster that was higher than the resulting mean LFC of 1.6 (median P-value $= 0.108$) with the CV cutoff of 0.2.}

The clustering refers to the aforementioned results, where we discuss the main and second cluster. StoatyDive has generally two modes, which you see in figure 7: evaluation (CV distribution) and classification (clustering). Both can be used to filter for peak profile shapes. The table assess the power of both modes for different peak length (sizes).
\end{answer}

Figure 5 (and related text) - I don't understand what Fig. 5a and b are plotting, and perhaps more broadly what is being done here? The text seems to indicate that the authors used 10 peaks defined as broad, 10 as sharp, and 10 as plateau and considered what the other tools did, but then state that they ran StoatyDive on these (with 0.87 accuracy), so I'm confused where the initial definition of broad / sharp / plateau came from (my initial impression was that they were from the StoatyDive analysis above, but that doesn't make sense with it giving different classification here).

\begin{answer}
We explain in the revised version of the paper: "We defined peaks as sharp (CV $>$ 1.0) and broad (CV $<$ 0.05) based on the calculated CV of StoatyDive. We selected peaks shaped like plateaus by inspecting them in a genome browser." We tried not to include a bias by our findings by StoatyDive. That is why, we did not take a pre-classified set by StoatyDive, just to show that StoatyDive classifies its own clusters correctly. However, we also needed a good way to decide what a sharp and broad peak is, thus we took the CV of StoatyDive and stayed very restrictive with the choice of a broad and sharp peak. However as seen in our results, if a broad peak resembles in its shape a sharp peak it can happen that StoatyDive makes some mistakes.
\end{answer}

I'm then confused what is being displayed in Fig. 5a and b - how does Fig. 5a show a few peaks sorted incorrectly? What do the dimensions/axes mean? Similarly, for 5c and 5d, what are the colored lines versus grey lines?

\begin{answer}
Figure 5a shows the dimensionally reduced space by umap of StoatyDive. Thus Dim1 and dim2 are the features found by umap, like PC1 and PC2 for principal component analysis. It is maybe not clearly visible, since all three clusters are clearly separated with 10 peaks in each clusters. But StoatyDive indeed has two peaks in cluster 1 which should belong to cluster 2 and 2 peaks in cluster 2 which should belong to cluster 1.

Figure 5b is a different tool called SIC-ChIP. They used in their analysis pre-defined features, such as half of the width of the peak at half of the maximum $w_{h/2}$. They defined for SIC-ChIP 5 features to classify the profiles. We showed in the paper only one plot with the highest cluster separation (variance), but the others can be found in the Supplementary 5.

Regarding the colored lines we state now in the caption of figure 5: "The coloured lines show the profiles of the related cluster, whereas the grey lines correspond to all other profiles."
\end{answer}

"Figure 6b shows more clearly that proteins with more than one RRM tend to have more sharper peaks." - this needs some sort of significance test

\begin{answer}
We included a significance test for this statement saying: "Figure 6b shows more clearly that proteins with more than one RRM tend to have sharper peaks (two-sided Wilcoxon test P-value ≈ 0.046)."
\end{answer}

Minor comments:

The text (particularly the new sections) would benefit from additional editing to improve text clarity - statements like "huge CV difference" should be avoided

\begin{answer}
Thank you very much for your suggestion. We looked into the text and removed or changed fuzzy adjectives and adverbs.
\end{answer}

\section{Reviewer 2}

The resubmitted manuscript by Florian Heyl and Rolf Backofen shows a good improvement. The authors addressed all the reviewer's comments in detail and restructured the manuscript accordingly. There are still a lot of interesting observations that would need further investigation but as the authors already mentioned, it would be beyond the scope of this paper. Overall, the manuscript is well written, and the method would be an interesting tool for the CLIP community. There are just few minor comments/suggestions, which are listed below:

1. Adding a library complexity distribution (number of peaks or a number of uniquely mapped reads) from each sample into the final CV distribution figures (Figure 2, Supplements1.) would be very helpful to interpret the results.

\begin{answer}
Thank you very much, indeed this would benefit the paper. We included the number of uniquely mapped reads for each sample.

The library complexity of replicate two of the SLBP data had about 0.4 times more uniquely mapped reads than replicate one. However, experiments such as TAF15 or RBM22 had almost a similar complexity, but different CV distributions. Furthermore, the number of uniquely mapped reads could not be linked to a higher or lower mean CV since experiments such as CPSF6 and HNRNPA1 (higher number of reads leads to a higher mean CV) show an opposite behavior than experiments such as TARDBP and U2AF1 (higher number of reads leads to a lower mean CV). Furthermore, StoatyDive also normalizes for the read coverage and investigates just the shape of the profile and the variance that is normalized by the mean coverage.
\end{answer}

2. Same for the Figure 3, if the authors could add a number of exonic and intronic peaks from each sample or the ratio.

\begin{answer}
We added the number of exonic and intronic peaks as well as the ratio in the caption of the picture.

We also randomly sub-sampled the exonic and intronic peaks for both replicates to 150 to test if the sample size has an influence on the difference. We got for the exonic peaks a mean CV of 0.53 for replicate 1 and 0.93 for replicate 2, and a p-value of the Wilcoxen test of < 0.05, which was quite similar to the results without sub-sampling (with mean CVs 0.48 and 0.90). The same was observable for the intronic peaks with a mean CV of 0.23 for replicate 1 and 1.11 for replicate 2 (p-value < 0.05). The results in the paper were 0.23 and 1.26 as a mean CV for the replicates.
\end{answer}

3. Page 6: "Furthermore, the proteins HNRNPM, U2AF2, and PTBP1 have all more than 2 RRMs (3, 3, and 4, respectively), which led to the assumption that an increasing number of RRMs results in more

sharper peaks. Figure 6b shows more clearly that proteins with more than one RRM tend to have more sharper peaks." - The authors assumption could be misleading without supporting literature or further analyses since there are not enough RBPs in their analysis to be confident enough. It would be better if authors could discuss this as one of the possibilities in the discussion section. There are also specific RBPs which are known to interact with RBP complexes, which could also affect the shape of the binding profile.

\begin{answer}
Thank you very much for pointing out that there is an issue with the discussion. We now state in the paper: "Furthermore, the proteins HNRNPM, U2AF2, and PTBP1 have all more than 2 RRMs (3, 3, and 4, respectively), thus it might be possible that an increasing number of RRMs results in sharper peaks. Figure~\ref{fig:proteins}b shows more clearly that proteins with more than one RRM tend to have sharper peaks (two-sided Wilcoxon test P-value ≈ 0.046). It is possible that RNA-proteins interactions become more specific with > 1 RRM and so the separation between more specific and unspecific binding sites is stricter. However, we had not taken any other RNA binding domains into account apart from RRMs and we would need to investigate more proteins to be confident about this trend."

We hope that this fits your suggestions.
\end{answer}

\section{Final remark:}

We thank all reviewers for their comments, critics, ideas and questions. It was very helpful and improved the paper a lot. You will find changes in the paper marked in red. Furthermore we changed the supplements 1. Please take a look. Thank you very much for your time and effort.

Close
