# Supplementary material for: StoatyDive: Evaluation and classification of peak profiles for sequencing data
Source: Gigascience. 2021 Jun 18;10(6):giab045. doi: 10.1093/gigascience/giab045 (PMC8212874; doi:10.1093/gigascience/giab045)
Supplement: giab045_Response_to_Reviewer_Comments_Revision_2 [file giab045_response_to_reviewer_comments_revision_2.pdf]

Author's Response To Reviewer Comments

Close

\section{Reviewer 1}
I appreciate the authors' effort, and they have addressed most of my concerns

My main remaining issue is that there are a large number of instances where the authors leave threads either as superficial data summaries or hanging as "it is worth to investigate…"; I don't find these helpful, either they are important for proving the utility of StoatyDive (and should be clarified and included) or are not (and maybe are better left out or mentioned in the discussion section).

\begin{answer}
We thank the reviewer for the suggestion. It was a bit difficult to know, which sections and comments the reviewer referred to. We further revised the text for better clarity in reflecting such kind of statement.

We removed instances of "it is worth to investigate" from the paper. All other "worth of investigate" are either explained in the paper or used as a phrase for an outlook in the conclusion section. We also removed or rephrased a lot of sentences with "might" or phrases that were still vague.
\end{answer}

While many of these I think can be corrected with simple text editing, for me in the first section that issue remains - there's 3 figures and significant text devoted to this section, and at the end of that section I still don't have a good sense of what the authors think is going on here with respect to the CV distribution between replicates. Is it within range of what's expected? Surprisingly low enough to suggest a data problem with replicate 1?

\begin{answer}
We state now in the paper: "The CV distribution of the input control was expected because an ideal control experiment should contain no real or not enriched binding events, that is to say, the value of all CVs is expected to be very small and close to 0 (see Supplementary Figure 1). But, the CV distribution of replicate 1 was more similar to the control experiment than to replicate 2. A CLIP experiment should result into a peak set with enriched regions and thus more specific peaks. The distribution of replicate 1 indicates some degree of variability in the binding events. However, we have to stress out that this does not necessarily depicts a poor quality of replicate 1 and 2."

And also in the methods section: "The user receives a first impression of the binding specificity of the protein of interest from the CV distribution. An unspecific binder has a CV distribution ≈ 0. A more specific binder has a CV distribution > 0. The CV distribution can also be used as a quality control to compare control and signal experiments. A quality breach might have occurred if the distributions of the control and signal experiment almost look identical. A control experiment should normally have a CV distribution ≈ 0, with only a very few binding sites showing higher CVs. A CLIP experiment, on the other hand, should contain more peaks with higher CVs and thus have a CV distribution that significantly differs in comparison to the control (Wilcoxon P-value < 0.05, see Supplementary Figure 1)."

For both paragraphs we generated a new plot that we included in the Supplementary Figure 1, stating in the paper: "Furthermore, we report a plot for the mean CV for the CLIP data in comparison to the size-matched input control of each protein. The control data tends to have a CV close to 0. The CV distributions between CLIP and control data always have a two-sided Wilcoxon test P-value < 0.05."

Consequently, we show empirically the difference of the CV distribution between CLIP and control data.
\end{answer}

I appreciate that they edited the language to weaken the claim that the data itself is irreproducible, but I think they may have misinterpreted my prior review comment in that sense - my concern wasn't really

inclusion of that claim, it was having analyses to validate it. I don't think it makes sense to simply write around this question in this way - it's a critical part of convincing that the method is of broad utility.

To phrase it a different way - I think the rest of the manuscript is now solidly presented once you have confidence that the CV metric is robust. But I think it's still missing evidence for whether the difference between replicates is biological / experimental noise or simply reflects extreme sensitivity / variability of the CV metric, and if it's the latter then it's problematic for believing the downstream analyses are robust.

Maybe as a more straightforward question - if one dataset (say the SLBP Replicate 2 one) is simply downsampled by splitting it in half into two pseudo-replicates, how consistent are the CV values for each peak? I think that would be convincing that the results seen in Fig 2 and 3 are real rather than noise.

\begin{answer}
Thank you very much for this question so that we can clarify. We tested your suggestion and split the dataset for replicate two into two pseudo-replicates using samtools (v 1.12). we state in the paper:

"We checked the robustness of the CV distribution and split the second replicate into two pseudo-replicates. We took randomly 50\%, with and without replacement, of the reads for pseudo-replicate 1 and the other half for pseudo-replicate 2. Both scenarios gave us similar results. Without replacement, replicate 1 had a mean CV of ≈ 1.05 and replicate 2 a value of ≈ 1.03, with a P-value of two-sided Wilcoxon test of 0.768. With replacement, replicate 1 had a mean CV of ≈ 1.05 and replicate 2 a value of ≈ 1.02, with a P-value of the Wilcoxon test of 0.7546. For example, the sharp peak Figure~\ref{fig:example}a had a CV in both scenarios of ≈ 5.2 and ≈ 5.3 for pseudo-replicate 1, and ≈ 4.8 and ≈ 5.4 for pseudo-replicate 2. The peak Figure~\ref{fig:example}a was in the peak list on position 47 and 42 for pseudo-replicate 1, and 52 and 45 for pseudo-replicate 2. The broad peak Figure~\ref{fig:example}b had a CV ≈ 0.001 (position 662) and ≈ 0.001 (position 658) for replicate 1, and ≈ 0.001 (position 667) and ≈ 0.001 (position 655) for replicate 2. A peak stayed as sharp or broad despite the random sampling. Thus, the overall trend of the CV distribution and the peak position in the list were robust to some random noise."
\end{answer}

\section{Final remark:}

We thank the reviewer for his suggestion. All changes are marked in red. We included a new figure in the Supplementary Figure 1.

Close
