# Supplementary material for: StoatyDive: Evaluation and classification of peak profiles for sequencing data
Source: Gigascience. 2021 Jun 18;10(6):giab045. doi: 10.1093/gigascience/giab045 (PMC8212874; doi:10.1093/gigascience/giab045)
Supplement: giab045_Response_to_Reviewer_Comments_Revision_3 [file giab045_response_to_reviewer_comments_revision_3.pdf]

Author's Response To Reviewer Comments

Close

Dear Florian,

Your manuscript "StoatyDive: Evaluation and Classification of Peak Profiles for Sequencing Data" (GIGA-D-20-00218R3) has successfully been reviewed and curated. Based on the reports, and my own assessment as Editor, I am pleased to inform you that it is acceptable for publication in GigaScience, once you have carried out some final editing and formatting changes to meet our policies. I've attached a very of the paper with these annotated on, but the main one is changing the final data section to a Data Availability section and citing the GigaDB DOI. This needs to be cited in the end of the paper as follows:

Heyl F; Backofen R (2021): Supporting data for "StoatyDive: Evaluation and Classification of Peak Profiles for Sequencing Data" GigaScience Database. http://dx.doi.org/10.5524/100896

And please also follow these guidelines for citing software in the paper:

https://f1000research.com/articles/9-1257/v2

Once you have made the necessary corrections, please submit a revised manuscript online at:

https://www.editorialmanager.com/giga/

If you have forgotten your username or password please use the "Send Login Details" link to get your login information. For security reasons, your password will be reset.

Please include a point-by-point within the 'Response to Reviewers' box in the submission system. Please ensure you describe additional experiments that were carried out and include a detailed rebuttal of any criticisms or requested revisions that you disagreed with. Please also ensure that your revised manuscript conforms to the journal style, which can be found in the Instructions for Authors on the journal homepage. If the data and code has been modified in the revision process please be sure to update the public versions of this too.

The due date for submitting the revised version of your article is 11 Aug 2021.

We look forward to receiving your revised manuscript soon.

Best wishes,

Scott Edmunds, Ph.D.
GigaScience
www.gigasciencejournal.com

Thank you very much for the revision. We have made the necessary amendments.

Thank you very much for your time and have a good week.

Best wishes,
Florian Heyl

Close
