# Supplementary material for: StoatyDive: Evaluation and classification of peak profiles for sequencing data
Source: Gigascience. 2021 Jun 18;10(6):giab045. doi: 10.1093/gigascience/giab045 (PMC8212874; doi:10.1093/gigascience/giab045)
Supplement: giab045_Reviewer_1_Report_Original_Submission — Eric Van Nostrand -- 8/22/2020 Reviewed [file giab045_reviewer_1_report_original_submission.pdf]

Reviewer Report

Title: StoatyDive: Evaluation and Classification of Peak Profiles for Sequencing Data

Version: Original Submission **Date:** 8/22/2020

Reviewer name: Eric Van Nostrand

Reviewer Comments to Author:

In the manuscript by Heyl & Backofen, the authors describe their analysis of peak shape as a feature to include in developing deeper understanding of RNA binding protein function and interpretation of CLIP-seq data. They describe a tool (StoatyDive) for performing peak-level calculation of coefficient of variation, followed by clustering or classification to distinguish groups of peaks by different patterns of read variance within the peak. The concept of peak shape correlating with biological properties of identified peaks is intriguing, and the authors describe a useful tool for performing such calculation quickly
Major comments:
1) I find that there's an imbalance where the most important findings are stated very briefly (sometimes without figures), whereas significant text and figure space is devoted to more descriptive analyses that lack a clear conclusion.
For example, to me the most important finding from the method is that the use of different peak shapes enables separation of real versus artifact peaks, which is aided by the authors use of SLBP (which has a well-characterized specific role in binding a specific hairpin structure in histone 3' UTRs), However, this result is stated in Table 1 without statistical analysis (enrichment, significance, etc) and a couple sentences of text. The manuscript would be stronger with additional analysis and description here (what is the sensitivity/specificity tradeoff in using the shape group / CV cutoffs described here? How do those change with different cutoffs?)
In contrast, two paragraphs are devoted to a fairly superficial motif analysis - it's not clear from the text which motifs the authors believe are real and which are false-positives, which makes it descriptive but unclear what the conclusion is (other than that they are 'different'). Although this does not appear to be discussed in the text, it seems that the GCUCUUU motif matches the known SLBP hairpin (e.g. Fig 2 from Tan et al, PMID 23329046), which would again suggest that the sharp and plateau classes are real whereas the broad class motifs are artifacts; this should be more clear.
2) The authors use the ENCODE data, but then re-perform peak calling with PureCLIP to perform their analyses. This isn't necessarily a problem; however, in the context of the major point of the manuscript being discussion of peak shapes, I find it perplexing that this is done with no further discussion, as one of the main differences between PureCLIP and the CLIPper algorithm (which was used in the peak calls provided by ENCODE) is that CLIPper explicitly uses peak shape as a feature to perform spline-fitting as part of the cluster identification process (Lovci et al. PMID 24213538). I can thus see an argument for using a peak caller that does not incorporate this feature in order to enable downstream analyses comparing peak shape; but I do think there should be some ultimate discussion of whether the classification & clustering described here provides additional power over the spline-fitting used in

the initial CLIPper calls.
Specific comments:
The authors conclude a hypothesis that "peak profiles shaped like plateaus were mainly PCR duplicates that were less informative." - this is a conclusion that is not supported by data, but is both confusing (the eCLIP datasets contain unique molecular identifiers and thus shouldn't contain significant PCR duplicates) and also is easy to test from the data used (those peaks should have higher mutation rates or PCR duplication rates among pre-PCR-duplicate-removed reads than other peaks).
I'm confused by the sentence "We looked further and found sharper peaks located on stem loops targeted by SLBP such as RNU7-1 RNA ..." - do the authors believe these are real or artifact peaks? Either way there should be some discussion of why (and why they are specifically called out here)
Many figures need editing to be more visually clear. In addition to fonts (Fig. 3 and 4 in particular have multiple panels that illegibly small at standard resolution), the figures would be significantly clearer if additional labels are provided to enhance flow through the analyses in the paper. For example - Table 1 refers to separation of the 7 classes in Fig. 3c/d into 4 shape 'groups'; it would be helpful to have those labeled in Fig 3 as well.
I think it needs to be clearer exactly what the analysis being done in the last section (of other RBPs) - after multiple reads I'm still a bit confused how the earlier analysis (with unsupervised clustering and manual annotation of classes) connects with the last analysis (with a hard cutoff of 'sharp' vs 'broad' peaks). The text in this section (& Fig 7) refers to 'broader' versus 'sharper' shaped peaks; however I'm not clear on what defines those terms (the methods text suggests that for standard analysis the authors set a CV cutoff of 0.5 to define 'specific' versus 'unspecific'; I'm assuming but not clear that it's that and not manual annotation of unsupervised clustering classes as was done for SLBP?)

Level of Interest

Please indicate how interesting you found the manuscript: Choose an item.

Quality of Written English

Please indicate the quality of language in the manuscript: Choose an item.

Declaration of Competing Interests

Please complete a declaration of competing interests, considering the following questions:

- Have you in the past five years received reimbursements, fees, funding, or salary from an organisation that may in any way gain or lose financially from the publication of this manuscript, either now or in the future?
- Do you hold any stocks or shares in an organisation that may in any way gain or lose financially from the publication of this manuscript, either now or in the future?

- Do you hold or are you currently applying for any patents relating to the content of the manuscript?
- Have you received reimbursements, fees, funding, or salary from an organization that holds or has applied for patents relating to the content of the manuscript?
- Do you have any other financial competing interests?
- Do you have any non-financial competing interests in relation to this paper?

If you can answer no to all of the above, write 'I declare that I have no competing interests' below. If your reply is yes to any, please give details below.

I am co-founder, member of the Board of Directors, on the SAB, equity holder, and paid consultant for Eclipse BioInnovations, a genomics company developing RNA genomics kits and services. I do not believe that Eclipse will benefit or lose financially from publication of this manuscript.

I agree to the open peer review policy of the journal. I understand that my name will be included on my report to the authors and, if the manuscript is accepted for publication, my named report including any attachments I upload will be posted on the website along with the authors' responses. I agree for my report to be made available under an Open Access Creative Commons CC-BY license (http://creativecommons.org/licenses/by/4.0/). I understand that any comments which I do not wish to be included in my named report can be included as confidential comments to the editors, which will not be published.

Choose an item.

To further support our reviewers, we have joined with Publons, where you can gain additional credit to further highlight your hard work (see: https://publons.com/journal/530/gigascience). On publication of this paper, your review will be automatically added to Publons, you can then choose whether or not to claim your Publons credit. I understand this statement.

Yes Choose an item.
