# Supplementary material for: StoatyDive: Evaluation and classification of peak profiles for sequencing data
Source: Gigascience. 2021 Jun 18;10(6):giab045. doi: 10.1093/gigascience/giab045 (PMC8212874; doi:10.1093/gigascience/giab045)
Supplement: giab045_Reviewer_1_Report_Revision_1 — Eric Van Nostrand -- 12/15/2020 Reviewed [file giab045_reviewer_1_report_revision_1.pdf]

Reviewer Report

Title: StoatyDive: Evaluation and Classification of Peak Profiles for Sequencing Data

Version: Revision 1 **Date:** 12/15/2020

Reviewer name: Eric Van Nostrand

Reviewer Comments to Author:

I appreciate the effort of the authors to provide additional analyses and address previous concerns, and the sections with specific edits are greatly improved. However, while the authors have clarified in many locations, there remain others (especially in the added sections) where it remains very confusing exactly what dataset is being used for analyses or plotted in figures. I also have remaining concerns (particularly about the newly added sections), where the authors seem to assume by default that their CV metric is accurate and reliable and then use that to draw conclusions about the underlying data, but I think this assumption is something that needs to be shown by analysis in the paper and cannot be assumed.
I think the new section on replicates "Peak Profile Landscape Reveals Low Reproducibility of Binding Sites" is weak and detracts from the paper. Broadly, my major concern is that it's completely unclear whether the low reproducibility seen is a feature of the experimental eCLIP data or a technical property of their CV metric. Essentially, this section defines a new metric (the CV for each peak), sees low reproducibility in this metric across replicates, and then assumes that this must imply low reproducibility in data "However, we have to stress out that we are unsure if this is the result of the specificity of SLBP, the different quality of replicate 1 and 2, or a different source, for example, a bad immunoprecipitation." To me, this statement requires some sort of analysis (downsampling / replicate splitting?) to show what the reproducibility of their CV metric is to random sampling variability. It would also be helpful to show here real examples of peaks that the authors claim show low reproducibility across replicates, to visualize what exactly their analysis is claiming. In addition, the conclusion that their analysis indicates that the replicates are of differing quality is also a computationally testable question (for example, comparing the fold-enrichment at previously validated histone 3'UTR peaks), which would help to convince that this isn't simply reflecting low reproducibility of their metric itself. The authors do this somewhat in Sup Fig 1, but then just use that to conclude that many other RBPs are also variable across replicates; SLBP definitely seems worse than those other RBPs, but this difference should be quantified. If the authors simply mean 'there is high variability in the CV metric between replicates for some RBPs', this should be worded as such and avoid claims about reproducibility of the underlying data without further analysis.
I'm also unclear what the 'control' dataset used in Figure 2 (and some later analyses) is exactly - the author state that they ran PureCLIP "for each CLIP replicate separately, taking the input control into account", so what is the control peak set in Figure 2 (as I assume it can't have been PureCLIP run on the input with itself as a control)?
The conclusions here are also vague and non-specific - "For a downstream analysis, for example the prediction of sequence motifs, it is worth to either exclude replicate 1 or to investigate,
why the CV distribution of replicate 1 was very different to replicate 2. Thus given the inspection of

StoatyDive, the user can now decide to investigate the binding sites of replicate 1 and compare them with the input control or replicate 2." - why was it worth investigating replicates separately? I'm unclear what the authors think was improved by performing this analysis - they show that the replicates have differences in CV distribution, with replicate 1 resembling the control (as above, I don't understand what this control peak set represents), but I don't see anything other than the CV analysis to indicate that Rep1 is of worse quality than Rep2 (in peak enrichment for histone RNAs, motifs, peak enrichment above input, etc). Similarly, the authors then split the peaks by exonic and intronic and claim "It is not clear why this difference appeared, but our observations stress out how important it is to perform an experiment with replicates." - how do they stress this? I don't see any benefit that the replicates have provided beyond the ability to say that they're different; other than Replicate 2 having more peaks, it's not described whether those additional peaks are real or false positives, and thus whether replicate 2 is better or worse.
I similarly think there needs to be improved clarity in the "Optimizing StoatyDive with the data of SLBP" section. I don't understand this sentence "This was achieved by the second cluster, which pointed out that the cluster had to be carefully chosen in order to remove some noise in the data (artifacts)." - does this mean that the authors removed some peaks in this set? Moreover, what are 'main cluster' and 'second cluster'? I don't see these defined anywhere. Similarly, "Furthermore, the same size achieved the highest TPR (0.61) and MCC (0.68) with a CV threshold of 0.2. However, the enrichment was not as good as with the clustering resulting in a mean LFC of 1.6 (median P-value = 0.108)." - I am completely lost as to what 'the same size' or 'the clustering' are referring to here in terms of datasets. This section needs to be edited to clearly state what exact set of peaks is being referred to at each point.
Figure 5 (and related text) - I don't understand what Fig. 5a and b are plotting, and perhaps more broadly what is being done here? The text seems to indicate that the authors used 10 peaks defined as broad, 10 as sharp, and 10 as plateau and considered what the other tools did, but then state that they ran StoatyDive on these (with 0.87 accuracy), so I'm confused where the initial definition of broad / sharp / plateau came from (my initial impression was that they were from the StoatyDive analysis above, but that doesn't make sense with it giving different classification here). I'm then confused what is being displayed in Fig. 5a and b - how does Fig. 5a show a few peaks sorted incorrectly? What do the dimensions/axes mean? Similarly, for 5c and 5d, what are the colored lines versus grey lines?
"Figure 6b shows more clearly that proteins with more than one RRM tend to have more sharper peaks." - this needs some sort of significance test
Minor comments:
The text (particularly the new sections) would benefit from additional editing to improve text clarity - statements like "huge CV difference" should be avoided

Level of Interest

Please indicate how interesting you found the manuscript: Choose an item.

Quality of Written English

Please indicate the quality of language in the manuscript: Choose an item.

Declaration of Competing Interests

Please complete a declaration of competing interests, considering the following questions:

- Have you in the past five years received reimbursements, fees, funding, or salary from an organisation that may in any way gain or lose financially from the publication of this manuscript, either now or in the future?
- Do you hold any stocks or shares in an organisation that may in any way gain or lose financially from the publication of this manuscript, either now or in the future?
- Do you hold or are you currently applying for any patents relating to the content of the manuscript?
- Have you received reimbursements, fees, funding, or salary from an organization that holds or has applied for patents relating to the content of the manuscript?
- Do you have any other financial competing interests?
- Do you have any non-financial competing interests in relation to this paper?

If you can answer no to all of the above, write 'I declare that I have no competing interests' below. If your reply is yes to any, please give details below.

I am co-founder, member of the Board of Directors, on the SAB, equity holder, and paid consultant for Eclipse BioInnovations, a genomics company developing RNA genomics kits and services. I do not believe that Eclipse will benefit or lose financially from publication of this manuscript.

I agree to the open peer review policy of the journal. I understand that my name will be included on my report to the authors and, if the manuscript is accepted for publication, my named report including any attachments I upload will be posted on the website along with the authors' responses. I agree for my report to be made available under an Open Access Creative Commons CC-BY license (http://creativecommons.org/licenses/by/4.0/). I understand that any comments which I do not wish to be included in my named report can be included as confidential comments to the editors, which will not be published.

Choose an item.

To further support our reviewers, we have joined with Publons, where you can gain additional credit to further highlight your hard work (see: https://publons.com/journal/530/gigascience). On publication of this paper, your review will be automatically added to Publons, you can then choose whether or not to claim your Publons credit. I understand this statement.

Yes Choose an item.
