# Supplementary material for: StoatyDive: Evaluation and classification of peak profiles for sequencing data
Source: Gigascience. 2021 Jun 18;10(6):giab045. doi: 10.1093/gigascience/giab045 (PMC8212874; doi:10.1093/gigascience/giab045)
Supplement: giab045_Reviewer_1_Report_Revision_2 — Eric Van Nostrand -- 3/16/2021 Reviewed [file giab045_reviewer_1_report_revision_2.pdf]

Reviewer Report

Title: StoatyDive: Evaluation and Classification of Peak Profiles for Sequencing Data

Version: Revision 2 **Date:** 3/16/2021

Reviewer name: Eric Van Nostrand

Reviewer Comments to Author:

I appreciate the authors' effort, and they have addressed most of my concerns
My main remaining issue is that there are a large number of instances where the authors leave threads either as superficial data summaries or hanging as "it is worth to investigate..."; I don't find these helpful, either they are important for proving the utility of StoatyDive (and should be clarified and included) or are not (and maybe are better left out or mentioned in the discussion section).
While many of these I think can be corrected with simple text editing, for me in the first section that issue remains - there's 3 figures and significant text devoted to this section, and at the end of that section I still don't have a good sense of what the authors think is going on here with respect to the CV distribution between replicates. Is it within range of what's expected? Surprisingly low enough to suggest a data problem with replicate 1? I appreciate that they edited the language to weaken the claim that the data itself is irreproducible, but I think they may have misinterpreted my prior review comment in that sense - my concern wasn't really inclusion of that claim, it was having analyses to validate it. I don't think it makes sense to simply write around this question in this way - it's a critical part of convincing that the method is of broad utility.
To phrase it a different way - I think the rest of the manuscript is now solidly presented once you have confidence that the CV metric is robust. But I think it's still missing evidence for whether the difference between replicates is biological / experimental noise or simply reflects extreme sensitivity / variability of the CV metric, and if it's the latter then it's problematic for believing the downstream analyses are robust.
Maybe as a more straightforward question - if one dataset (say the SLBP Replicate 2 one) is simply downsampled by splitting it in half into two pseudo-replicates, how consistent are the CV values for each peak? I think that would be convincing that the results seen in Fig 2 & 3 are real rather than noise.

Level of Interest

Please indicate how interesting you found the manuscript: Choose an item.

Quality of Written English

Please indicate the quality of language in the manuscript: Choose an item.

Declaration of Competing Interests

Please complete a declaration of competing interests, considering the following questions:

- Have you in the past five years received reimbursements, fees, funding, or salary from an organisation that may in any way gain or lose financially from the publication of this manuscript, either now or in the future?
- Do you hold any stocks or shares in an organisation that may in any way gain or lose financially from the publication of this manuscript, either now or in the future?
- Do you hold or are you currently applying for any patents relating to the content of the manuscript?
- Have you received reimbursements, fees, funding, or salary from an organization that holds or has applied for patents relating to the content of the manuscript?
- Do you have any other financial competing interests?
- Do you have any non-financial competing interests in relation to this paper?

If you can answer no to all of the above, write 'I declare that I have no competing interests' below. If your reply is yes to any, please give details below.

I am co-founder, member of the Board of Directors, on the SAB, equity holder, and paid consultant for Eclipse BioInnovations, a genomics company developing RNA genomics kits and services. I do not believe that Eclipse will benefit or lose financially from publication of this manuscript.

I agree to the open peer review policy of the journal. I understand that my name will be included on my report to the authors and, if the manuscript is accepted for publication, my named report including any attachments I upload will be posted on the website along with the authors' responses. I agree for my report to be made available under an Open Access Creative Commons CC-BY license (http://creativecommons.org/licenses/by/4.0/). I understand that any comments which I do not wish to be included in my named report can be included as confidential comments to the editors, which will not be published.

Choose an item.

To further support our reviewers, we have joined with Publons, where you can gain additional credit to further highlight your hard work (see: https://publons.com/journal/530/gigascience). On publication of this paper, your review will be automatically added to Publons, you can then choose whether or not to claim your Publons credit. I understand this statement.

Yes Choose an item.
