# Supplementary material for: StoatyDive: Evaluation and classification of peak profiles for sequencing data
Source: Gigascience. 2021 Jun 18;10(6):giab045. doi: 10.1093/gigascience/giab045 (PMC8212874; doi:10.1093/gigascience/giab045)
Supplement: giab045_Reviewer_2_Report_Original_Submission — Nejc Haberman -- 8/25/2020 Reviewed [file giab045_reviewer_2_report_original_submission.pdf]

Reviewer Report

Title: StoatyDive: Evaluation and Classification of Peak Profiles for Sequencing Data

Version: Original Submission **Date:** 8/25/2020

Reviewer name: Nejc Haberman

Reviewer Comments to Author:

Review
Journal: GigaScience
Manuscript ID: GIGA-D-20-00218
Title: StoatyDive: Evaluation and Classification of Peak Profiles for Sequencing Data
Category: Technical Note
The manuscript by Florian Heyl and Rolf Backofen presents StoatyDive, a new peak calling tool to identify binding sites from sequencing data such as CLIP-seq. The paper shows that the StoatyDive can identify different classes of binding sites, based on their peak shape profile. This information can be very useful to study binding specificity of RNA-binding proteins (RBPs). This tool can also be used as a quality control measure to identify specific and unspecific binding sites to filter out false positive bindings based on their peak shape profiles. The paper presents several interesting analyses, including SLBP protein, which shows interesting binding patterns but also a low reproducibility between the replicates. Overall, the manuscript is well written, the method is of broad utility, and all the analyses are well documented and their code is available online. However, as listed below, the manuscript has several shortcomings.
1. comment: I am surprised that the authors focused their analyses on the SLBP protein and not on the more well studied RBP. However, the authors did find interesting observations for the SLBP protein by analysing eCLIP samples in K562 cell lines, including low reproducibility between the replicates, which haven't been fully investigated in this manuscript. I think it would be important to address these observation in a more systematic manner:
* Have you checked how much the Coefficient of Variation (CV) changes between other replicates in other eCLIP samples, including eCLIP HepG2 samples? It would also be important to include CV results for all other samples that you analysed in this study (RBM22, U2AF2, PTBP1…). Do other eCLIP samples also show similar differences between the replicates or is the SLBP protein one of the exceptions? Is this variation between replicates more specific for the eCLIP protocol or is it also common in other CLIP-seq methods such as iCLIP, irCLIP or PAR-CLIP?
* How many peaks identified in replicate one overlap with replicate two?
* Is the library size similar between the replicates and have you done any more detailed quality controls between the replicates (number of uniquely mapped reads, sequencing errors etc.)? Maybe a poor quality or low coverage could explain the difference.
* Would it be possible that one replicate has more cytoplasmic binding sites than the other? One way of testing this would be to check how many peaks that you identify are in intronic regions vs exonic regions, for each replicate separately. Also, how does the CVs and shape profile of the peaks change between exonic and intronic peaks? Maybe this sample has a higher binding specificity in the nucleus

than in cytoplasm or vice versa.
2. comment: "It is important to note that peaks shaped like plateaus might be false positives. These peaks most likely corresponded to PCR duplicates that were not real binding sites. We have removed PCR duplicates during the pre-processing of the read library, but some duplicates might still be in the data. Sequencing errors in the unique molecular identifiers (UMI) are a common reason."
* Are there any changes in shape profiles between intronic and exonic peaks? To filter out false positive peaks, you could use ENCODE RNA-seq data for the same cell line and analyse peaks and their shapes in expressed transcripts. How many False positives are present in non-expressed transcripts and do they show different motifs and different shapes of their peaks?
3. comment: "We investigated also the sequence motifs of sharp and broad peak profiles of the protein RBFOX2 (eCLIP data from the study by Van Nostrand et al. [5]), because it has the conserved sequence motif TGCATG, which is enriched in the RBPFOX2's binding sites [13, 14, 15]. We have found the conserved motif only in sharp peaks (Table 3), but the broader profiles also had an enriched motif. It is worth investigating if RBFOX2 has some unspecific binding preferences with that specific motif. The results for RBFOX2 again demonstrated that different peak shape groups result in different sequence motifs."
* A recent paper by Bridget E. Begg et al. identified secondary binding motifs from their RBFOX2 CLIP experiments (Begg et al. 2020). These motifs are specific for intronic and 3'UTR regions (Bridget E. Begg et al., Figure 3). Could the authors also identify these motifs by separating their peak shape profiles into intronic and 3'UTR region?
4. comment: "All peaks were therefore extended or shrank to a length of 77 nucleotides. This was based on the observation that the third quartile of all peaks (from all proteins) was 77 nucleotides long."
* The 77 nucleotides window size for all RBPs could be too broad to identify sharp peaks for proteins with specific bindings. Anob M. Chakrabarti et al. has shown in their review (Chakrabarti et al. 2018), that the window size parameters can modify the sensitivity and specificity of the data and it needs to be adapted for each RBP. For example, PTBP1 can have multiple binding sites just next to each other, where the broad window size would detect all the protein binding sites as one large binding site instead of multiple sharp ones. Could the authors demonstrate and comment on at least one example of how the window size can affects peak finding specificity and their shape profiles?
5. comment: "So we investigated the number of peaks that fall into introns (90% overlap) for the proteins that are involved in the splicing process. The proteins PTBP1 (85%), RBM22 (62%), TARDBP (79%), and HNRNPM (87%) had more than 50% of peaks in introns, which detected the assumption of split peaks. However, the proteins U2AF1 (17%), and U2AF2 (15%) had more peaks in exon regions, where the possibility of split peaks might still occur. We found for U2AF1 only 27 peaks (0.72%) and for U2AF2 5 peaks (0.40%) that are potential split peaks, again defecting the assumption of a technical artefact in the peak set of splicing factors. Thus, the sharpness of the peaks of U2AF1 and U2AF2 was potentially not the result of the peak calling."
* The authors examined RBPs that are involved in the splicing process, including U2AF1 and UAF2, which recognises 3' splice sites and recruits the spliceosome. The authors claim that the majority of the identified peaks from both proteins fall into the exonic region. This statement contrasts with previous studies including my preliminary analysis (see Figure ??? below). The figure shows a normalised coverage of cDNA-starts for the same eCLIP U2AF1 and U2AF2 K562 and HepG2 samples relative to the

3' splice site region, showing a dominant enrichment of the intronic region for both proteins which also agrees with the previous CLIP studies (Zarnack et al. 2013; Sutandy et al. 2018; Briese et al. 2019). The authors should examine their peak analyses in these results further to identify the reason for such a difference.

References

Begg, BE, M Jens, PY Wang, CM Minor, and CB Burge. 2020. "Concentration-Dependent Splicing Is Enabled by Rbfox Motifs of Intermediate Affinity.". Nat Struct Mol Biol.

Chakrabarti, Anob M., Nejc Haberman, Arne Praznik, Nicholas M. Luscombe, and Jernej Ule. 2018. "Data Science Issues in Studying ProteinRNA Interactions with CLIP Technologies". Annual Review of Biomedical Data Science 1 (1): 235-61. https://doi.org/10.1146/annurev-biodatasci-080917-013525.

Zarnack, Kathi, Julian KÃ¶nig, Mojca Tajnik, IÃ±igo Martincorena, Sebastian Eustermann, Isabelle StÃ©vant, Alejandro Reyes, Simon Anders, Nicholas M. Luscombe, and Jernej Ule. 2013. "Direct Competition between HnRNP C and U2AF65 Protects the Transcriptome from the Exonization of Alu Elements". Cell 152 (3): 453-66. https://doi.org/10.1016/j.cell.2012.12.023.

Sutandy, F.X. Reymond, Stefanie Ebersberger, Lu Huang, Anke Busch, Maximilian Bach, Hyun-Seo Kang, JÃ¶rg Fallmann, et al. 2018. "In Vitro ICLIP-Based Modeling Uncovers How the Splicing Factor U2AF2 Relies on Regulation by Cofactors". Genome Research 28 (5): 699-713. https://doi.org/10.1101/gr.229757.117.

Briese, M, N Haberman, CR Sibley, R Faraway, AS Elser, AM Chakrabarti, Z Wang, et al. 2019. "A Systems View of Spliceosomal Assembly and Branchpoints with ICLIP.". Nat Struct Mol Biol 26: 930-40.

Level of Interest

Please indicate how interesting you found the manuscript: Choose an item.

Quality of Written English

Please indicate the quality of language in the manuscript: Choose an item.

Declaration of Competing Interests

Please complete a declaration of competing interests, considering the following questions:

- Have you in the past five years received reimbursements, fees, funding, or salary from an organisation that may in any way gain or lose financially from the publication of this manuscript, either now or in the future?
- Do you hold any stocks or shares in an organisation that may in any way gain or lose financially from the publication of this manuscript, either now or in the future?
- Do you hold or are you currently applying for any patents relating to the content of the manuscript?

- Have you received reimbursements, fees, funding, or salary from an organization that holds or has applied for patents relating to the content of the manuscript?
- Do you have any other financial competing interests?
- Do you have any non-financial competing interests in relation to this paper?

If you can answer no to all of the above, write 'I declare that I have no competing interests' below. If your reply is yes to any, please give details below.

I declare that I have no competing interests

I agree to the open peer review policy of the journal. I understand that my name will be included on my report to the authors and, if the manuscript is accepted for publication, my named report including any attachments I upload will be posted on the website along with the authors' responses. I agree for my report to be made available under an Open Access Creative Commons CC-BY license (http://creativecommons.org/licenses/by/4.0/). I understand that any comments which I do not wish to be included in my named report can be included as confidential comments to the editors, which will not be published.

Choose an item.

To further support our reviewers, we have joined with Publons, where you can gain additional credit to further highlight your hard work (see: https://publons.com/journal/530/gigascience). On publication of this paper, your review will be automatically added to Publons, you can then choose whether or not to claim your Publons credit. I understand this statement.

Yes Choose an item.
