# Supplementary material for: StoatyDive: Evaluation and classification of peak profiles for sequencing data
Source: Gigascience. 2021 Jun 18;10(6):giab045. doi: 10.1093/gigascience/giab045 (PMC8212874; doi:10.1093/gigascience/giab045)
Supplement: giab045_Reviewer_2_Report_Revision_1 — Nejc Haberman -- 12/15/2020 Reviewed [file giab045_reviewer_2_report_revision_1.pdf]

Reviewer Report

Title: StoatyDive: Evaluation and Classification of Peak Profiles for Sequencing Data

Version: Revision 1 **Date:** 12/15/2020

Reviewer name: Nejc Haberman

Reviewer Comments to Author:

The resubmitted manuscript by Florian Heyl and Rolf Backofen shows a good improvement. The authors addressed all the reviewer's comments in detail and restructured the manuscript accordingly. There are still a lot of interesting observations that would need further investigation but as the authors already mentioned, it would be beyond the scope of this paper. Overall, the manuscript is well written, and the method would be an interesting tool for the CLIP community. There are just few minor comments/suggestions, which are listed below:

1. Adding a library complexity distribution (number of peaks or a number of uniquely mapped reads) from each sample into the final CV distribution figures (Figure 2, Supplements_1.) would be very helpful to interpret the results.
2. Same for the Figure 3, if the authors could add a number of exonic and intronic peaks from each sample or the ratio.
3. Page 6: "Furthermore, the proteins HNRNPM, U2AF2, and PTBP1 have all more than 2 RRMs (3, 3, and 4, respectively), which led to the assumption that an increasing number of RRMs results in more sharper peaks. Figure 6b shows more clearly that proteins with more than one RRM tend to have more sharper peaks." - The authors assumption could be misleading without supporting literature or further analyses since there are not enough RBPs in their analysis to be confident enough. It would be better if authors could discuss this as one of the possibilities in the discussion section. There are also specific RBPs which are known to interact with RBP complexes, which could also affect the shape of the binding profile.

Level of Interest

Please indicate how interesting you found the manuscript: Choose an item.

Quality of Written English

Please indicate the quality of language in the manuscript: Choose an item.

Declaration of Competing Interests

Please complete a declaration of competing interests, considering the following questions:

- Have you in the past five years received reimbursements, fees, funding, or salary from an organisation that may in any way gain or lose financially from the publication of this manuscript, either now or in the future?
- Do you hold any stocks or shares in an organisation that may in any way gain or lose financially from the publication of this manuscript, either now or in the future?
- Do you hold or are you currently applying for any patents relating to the content of the manuscript?
- Have you received reimbursements, fees, funding, or salary from an organization that holds or has applied for patents relating to the content of the manuscript?
- Do you have any other financial competing interests?
- Do you have any non-financial competing interests in relation to this paper?

If you can answer no to all of the above, write 'I declare that I have no competing interests' below. If your reply is yes to any, please give details below.

I declare that I have no competing interests

I agree to the open peer review policy of the journal. I understand that my name will be included on my report to the authors and, if the manuscript is accepted for publication, my named report including any attachments I upload will be posted on the website along with the authors' responses. I agree for my report to be made available under an Open Access Creative Commons CC-BY license (http://creativecommons.org/licenses/by/4.0/). I understand that any comments which I do not wish to be included in my named report can be included as confidential comments to the editors, which will not be published.

Choose an item.

To further support our reviewers, we have joined with Publons, where you can gain additional credit to further highlight your hard work (see: https://publons.com/journal/530/gigascience). On publication of this paper, your review will be automatically added to Publons, you can then choose whether or not to claim your Publons credit. I understand this statement.

Yes Choose an item.
