# Supplementary material for: StoatyDive: Evaluation and classification of peak profiles for sequencing data
Source: Gigascience. 2021 Jun 18;10(6):giab045. doi: 10.1093/gigascience/giab045 (PMC8212874; doi:10.1093/gigascience/giab045)
Supplement: giab045_Reviewer_3_Report_Original_Submission — William Lai -- 8/28/2020 Reviewed [file giab045_reviewer_3_report_original_submission.pdf]

Reviewer Report

Title: StoatyDive: Evaluation and Classification of Peak Profiles for Sequencing Data

Version: Original Submission **Date:** 8/28/2020

Reviewer name: William Lai

Reviewer Comments to Author:

Paper describes novel method of CLIP-seq peak classification by identifying unique sequence tag distributions embedded in the data. Authors use this algorithm on existing data (SLBP, RBFOX2, etc.) to identify a variety of patterns that are suspected to be related to biological function. Methodology seems consistent and well documented. Proper controls appear to have been included and to the best of my knowledge, the appropriate statistical tests and analyses appear to have been performed.
I note three issues with the manuscript in its current form:
* Figure 2 indicates that replicate 1 of SLBP CLIP-seq is indistinguishable from the negative control from the perspective of the coefficient of variation. However, this replicate is then used throughout the analyses of Figure 3 and Tables 1 & 2. If Replicate 1 did work experimentally, it would contradict the authors' proposal that evaluating the coefficient of variations across peak-profiles (Figure 2) is a valuable method of examining CLIP-seq quality control.
* Figure 3 shows the distinct patterns present across two replicates of SLBP CLIP-seq. It is unclear how unique these patterns are to SLBP CLIP-seq or if they are an artifact of CLIP-seq assay. It would be important to show the average tag distribution of the control experiment at sample peaks (separated by distinct peak profile) in order to demonstrate that the discovered peak profiles exist only within sample-targeted CLIP-seq. * * The underlying RNA sequence of the peaks should also be investigated with regards to whether mappability (or a specific A/U/G/C content) is responsible for producing broad vs narrow peaks. Are broad peaks a function of less mappable reads producing unnatural peaks? It would important to demonstrate that StoatyDive is not just modeling the underlying sequence mappability at the region.

Level of Interest

Please indicate how interesting you found the manuscript: Choose an item.

Quality of Written English

Please indicate the quality of language in the manuscript: Choose an item.

Declaration of Competing Interests

Please complete a declaration of competing interests, considering the following questions:

- Have you in the past five years received reimbursements, fees, funding, or salary from an organisation that may in any way gain or lose financially from the publication of this manuscript, either now or in the future?
- Do you hold any stocks or shares in an organisation that may in any way gain or lose financially from the publication of this manuscript, either now or in the future?
- Do you hold or are you currently applying for any patents relating to the content of the manuscript?
- Have you received reimbursements, fees, funding, or salary from an organization that holds or has applied for patents relating to the content of the manuscript?
- Do you have any other financial competing interests?
- Do you have any non-financial competing interests in relation to this paper?

If you can answer no to all of the above, write 'I declare that I have no competing interests' below. If your reply is yes to any, please give details below.

I declare that I have no competing interests

I agree to the open peer review policy of the journal. I understand that my name will be included on my report to the authors and, if the manuscript is accepted for publication, my named report including any attachments I upload will be posted on the website along with the authors' responses. I agree for my report to be made available under an Open Access Creative Commons CC-BY license (http://creativecommons.org/licenses/by/4.0/). I understand that any comments which I do not wish to be included in my named report can be included as confidential comments to the editors, which will not be published.

Choose an item.

To further support our reviewers, we have joined with Publons, where you can gain additional credit to further highlight your hard work (see: https://publons.com/journal/530/gigascience). On publication of this paper, your review will be automatically added to Publons, you can then choose whether or not to claim your Publons credit. I understand this statement.

Yes Choose an item.
